# The Still Bay and Howiesons Poort at Sibudu and Blombos: Understanding Middle Stone Age Technologies

**DOI:** 10.1371/journal.pone.0131127

**Published:** 2015-07-10

**Authors:** Sylvain Soriano, Paola Villa, Anne Delagnes, Ilaria Degano, Luca Pollarolo, Jeannette J. Lucejko, Christopher Henshilwood, Lyn Wadley

**Affiliations:** 1 ArScAn, AnTET, Université Paris Ouest, CNRS, 92023 Nanterre, France; 2 University of Colorado Museum, Boulder, Colorado 80309–0265, United States of America; 3 School of Geography, Archaeology and Environmental Studies, University of the Witwatersrand, Johannesburg 2050, South Africa; 4 CNRS-PACEA, Université de Bordeaux, 33615 Pessac, France; 5 Evolutionary Studies Institute, University of the Witwatersrand, Johannesburg 2050, South Africa; 6 Dipartimento di Chimica and Chimica Industriale, Università di Pisa, 56126 Pisa, Italy; 7 Laboratoire Archéologie et Peuplement de l’Afrique, Anthropology Unit, Department of Genetics and Evolution, University of Geneva, 1211 Geneva 4, Switzerland; 8 Institute for Archaeology, History, Culture and Religious Studies, University of Bergen, 5007 Bergen, Norway; University of Oxford, UNITED KINGDOM

## Abstract

The classification of archaeological assemblages in the Middle Stone Age of South Africa in terms of diversity and temporal continuity has significant implications with respect to recent cultural evolutionary models which propose either gradual accumulation or discontinuous, episodic processes for the emergence and diffusion of cultural traits. We present the results of a systematic technological and typological analysis of the Still Bay assemblages from Sibudu and Blombos. A similar approach is used in the analysis of the Howiesons Poort (HP) assemblages from Sibudu seen in comparison with broadly contemporaneous assemblages from Rose Cottage and Klasies River Cave 1A. Using our own and published data from other sites we report on the diversity between stone artifact assemblages and discuss to what extent they can be grouped into homogeneous lithic sets. The gradual evolution of debitage techniques within the Howiesons Poort sequence with a progressive abandonment of the HP technological style argues against the saltational model for its disappearance while the technological differences between the Sibudu and Blombos Still Bay artifacts considerably weaken an interpretation of similarities between the assemblages and their grouping into the same cultural unit. Limited sampling of a fragmented record may explain why simple models of cultural evolution do not seem to apply to a complex reality.

## Introduction

In South Africa two Late Pleistocene industries, the Still Bay (SB) and Howiesons Poort (HP) have attracted a lot of attention because of their complexity which combine distinctive lithic markers with innovative bone and stone technologies and symbolic and social practices. These assemblages have played a central role in publications of the last fifteen years about the evolution of modern human behavior based on evidence first reported from the Howiesons Poort levels of Klasies River and then from the earlier Still Bay levels of Blombos Cave. A comprehensive account of the historical background, novel technologies and production of symbolic artifacts of these two phases can be found in [1–6].

Until very recently the generally accepted view of these two facies of the late Middle Stone Age was that they were very dynamic, innovative and homogeneous industries which lasted less than 10,000 years each, between c. 77 and 59 ka, with a temporal hiatus of some millennia between the two phases [7–9]. A shorter duration of the Still Bay, ca. 1000 years, was proposed [8]. However recently reported dates from the Diepkloof site (South Africa) are significantly complicating our views on cultural change in the region. According to these new dates [4,10] the SB and HP industries had a much longer duration than previously envisaged [8] comparable to those of broadly contemporaneous Middle Paleolithic industries in Europe, which also show clear spatio-temporal distributions [11–13]. It seems that systematic technological and typological analyses are necessary to clarify the nature and relations of assemblages assigned to the same lithic tradition yet apparently separated by large spans of time and not homogeneous in space.

**Fig 1:**
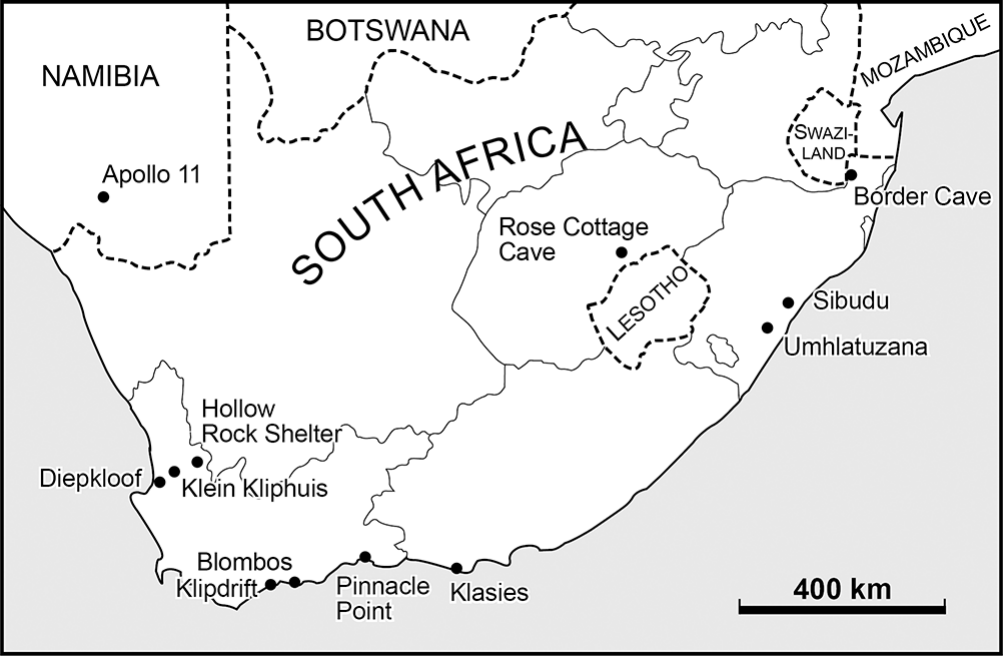
Geographic map with location of sites mentioned in the text.

In this paper we present research on the form, technology and function of SB and HP stone artifacts at the two sites of Sibudu and Blombos (Fig 1). Using our own and published data from other sites (mainly Rose Cottage and Klasies River Cave 1A), we report on the heterogeneity within and between the two phases and we discuss to what extent they can be defined as homogeneous sets [14–16]. The whole question of technological traits and patterns in both industries has not been fully dealt with in previous publications, yet they provide useful criteria for comparisons, as they are less affected by activity variation or unfavorable condition for preservation of organic artifacts. This is the case of a HP site like Rose Cottage, where charcoal was preserved but there was no bone [17] and of Hollow Rock Shelter, a SB site, where only stone artifacts remain [18]. Consistent technological criteria which occur repetitively help in classifying surface assemblages and in distinguish stratigraphic mixing from cultural continuity. In the study of reduction sequences we give importance to bifacial reduction, core reduction, and selection of blanks for tool production. Proportional occurrences of different tool classes are largely determined by dominant activities [19] and we view them as less useful than blank production and modes of retouch in interassemblage comparison. In brief, we view our paper as an attempt at a precise characterization of some important Late Pleistocene assemblages and a contribution to the general problem of cultural continuity or discontinuity in the Middle Stone Age of South Africa. The paper organization is described in the next section.

## Materials and Methods

### Permits

Sibudu: Permits were obtained from Amafa KwaZulu-Natal Heritage Agency in accordance with the KwaZuku-Natal Heritage Act of 2008. The excavation permit number is 007/09. The permit holder is Prof. Lyn Wadley. The Sibudu collections are housed in the Acacia unit of the Evolutionary Studies Institute at the University of the Witwatersrand, Johannesburg, South Africa.

Blombos: Excavation permits were issued by the Heritage Western Cape under section 35(4) of the National Heritage Resources Act no 25. The permit numbers are 2007-03-003 and 2011/09/001. The permit holder is Prof. Christopher Henshilwood. The Blombos Still Bay materials are housed in the Pre-Colonial Archaeology Department, Iziko Museum of South Africa, Cape Town, South Africa.

### Sorting and sampling

To facilitate interassemblage comparisons, we followed the sorting procedures used by us in the analysis of other South African MSA assemblages [20–23]. We select all cores, core fragments, tools, tool fragments and all blade and blade fragments regardless of size. Retouched pieces and cores are assigned individual catalogue numbers. Complete or broken flakes preserving the platform > 2 cm are also selected; for quartz the cutoff point was 1 cm. In the case of the Sibudu Still Bay we lowered the limit to 1 cm for all raw materials, due to the abundance of small-sized retouch and shaping flakes associated with the production of Still Bay bifacial points. Our sorting procedures exclude flake fragments (broken flakes without the platform), flakes < 2 or 1 cm and chunks from technological analysis; however the small debris is bagged by large categories and remains available for specific studies. This selection procedure has several advantages: a) it smoothes differences in sorting precision and screen size between excavations, b) it greatly accelerates the analysis of flaking methods by sorting out the small debris and flake fragments, and c) it sets an explicit cut-off point for calculating assemblage composition. Our sample for the Sibudu HP and the Still Bay comes mainly from four adjacent square meters (B5 and B6 excavated in 2002–2004 and C5, C6 excavated between 2007 and 2008) but to enlarge our Still Bay sample we added 19 bifacial pieces from the 2009 excavation which include a few pieces from squares B4 and C4.

### Paper organization

This paper includes three main sections: the Still Bay of Sibudu; the Still Bay of Blombos; the Howiesons Poort. 1. The Still Bay of Sibudu was, until now, known only from preliminary descriptions [24–26]. Our analysis is systematic and treats tools and debitage. 2. The analysis of the Blombos Still Bay relies on previous technological analyses of the bifacial points [27,28]. It includes now a sample of all points and retouched pieces excavated up to 2011. The study of the very large debitage sample (approximately 20,000 pieces) could not be conducted for this paper because the Iziko Museum of Cape Town has been closed for renovation for the past 3 years and the collections were not available for study. A program of sorting and coding of the debitage is beginning through loans and will be carried out in 2015 and the coming year. 3. Several technological analyses of Howiesons Poort assemblages have been published in recent years [2,3,20,21,29]; thus in our analysis of the Sibudu HP we adopted a comparative perspective. We discuss raw material transport distances, blade manufacture, retouched tool production and use, impact scars, new evidence for hafting adhesives and technological changes through time. Our comparisons are based on data from HP assemblages at Rose Cottage and Klasies of which we have direct knowledge [20,21] and to a minor degree on publications of other sites.

### Methods

The study of lithic assemblages is based on quantitative and qualitative analyses including metrical, technological and typological attributes combined with a particular attention to the sequence of manufacture and reworking of Still Bay points at Sibudu, in comparison with the Still Bay points of Blombos. Details of the site context and the stratigraphic sequences of Sibudu and Blombos are discussed by section; detailed analytical protocols are provided in the Supporting Information. Still Bay and Howiesons Poort primary data used here are available both in the body of the paper and in Supporting Information. Supporting Information for the Still Bay at Sibudu and Blombos is provided in S1 File, S2 File and S3 File. Supporting Information for the Howiesons Poort is provided in S4 File, S5 File and S6 File.

## Results

### The Still Bay of Sibudu

#### Sibudu Cave. The site and the sequence

**Fig 2:**
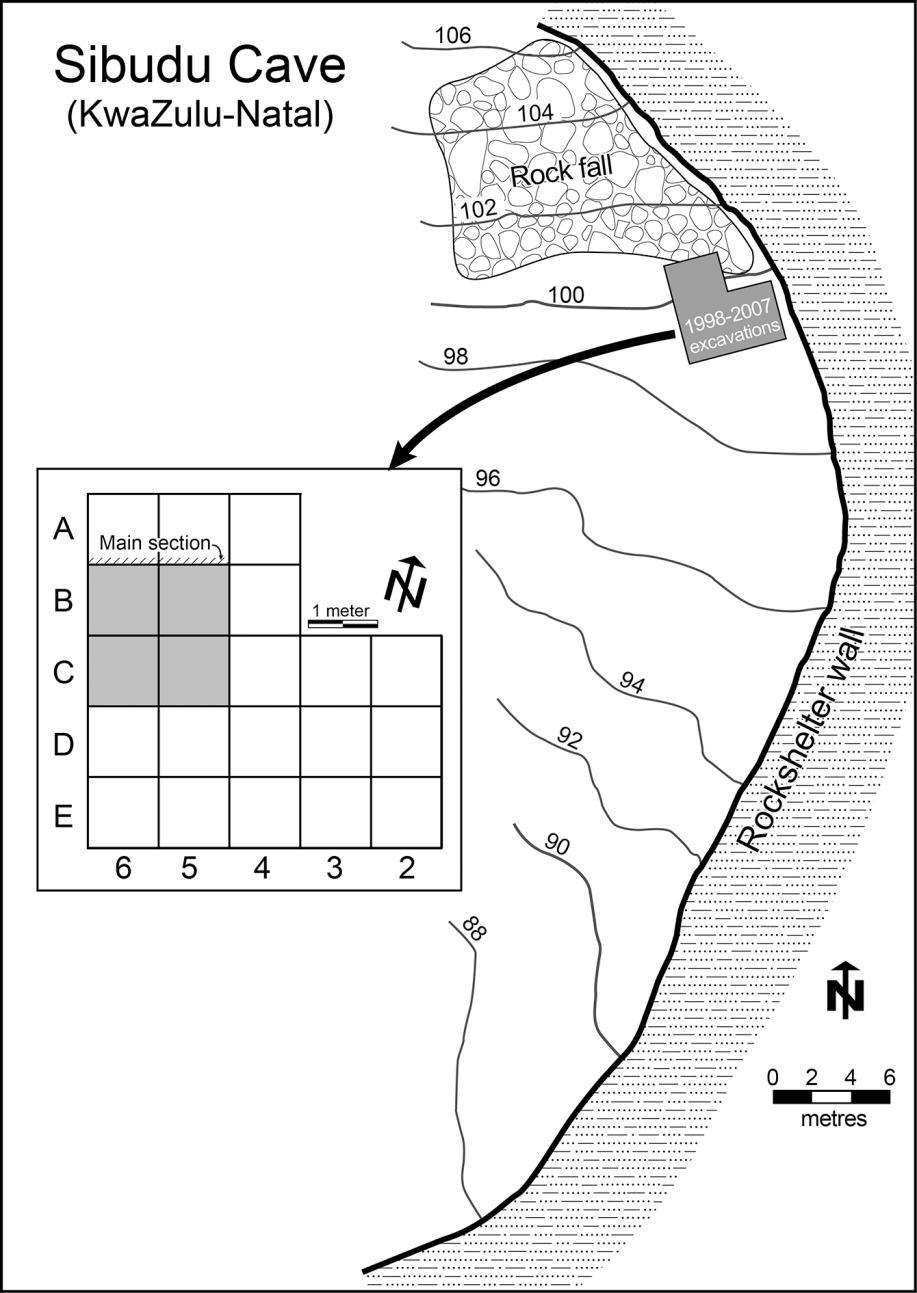
Sibudu: Plan of the site with elevations in meters above sea level, the excavation grid and location of main section. The Still Bay and Howiesons Poort materials studied in this paper come from the four square meters shaded grey (modified after [30]).

**Fig 3:**
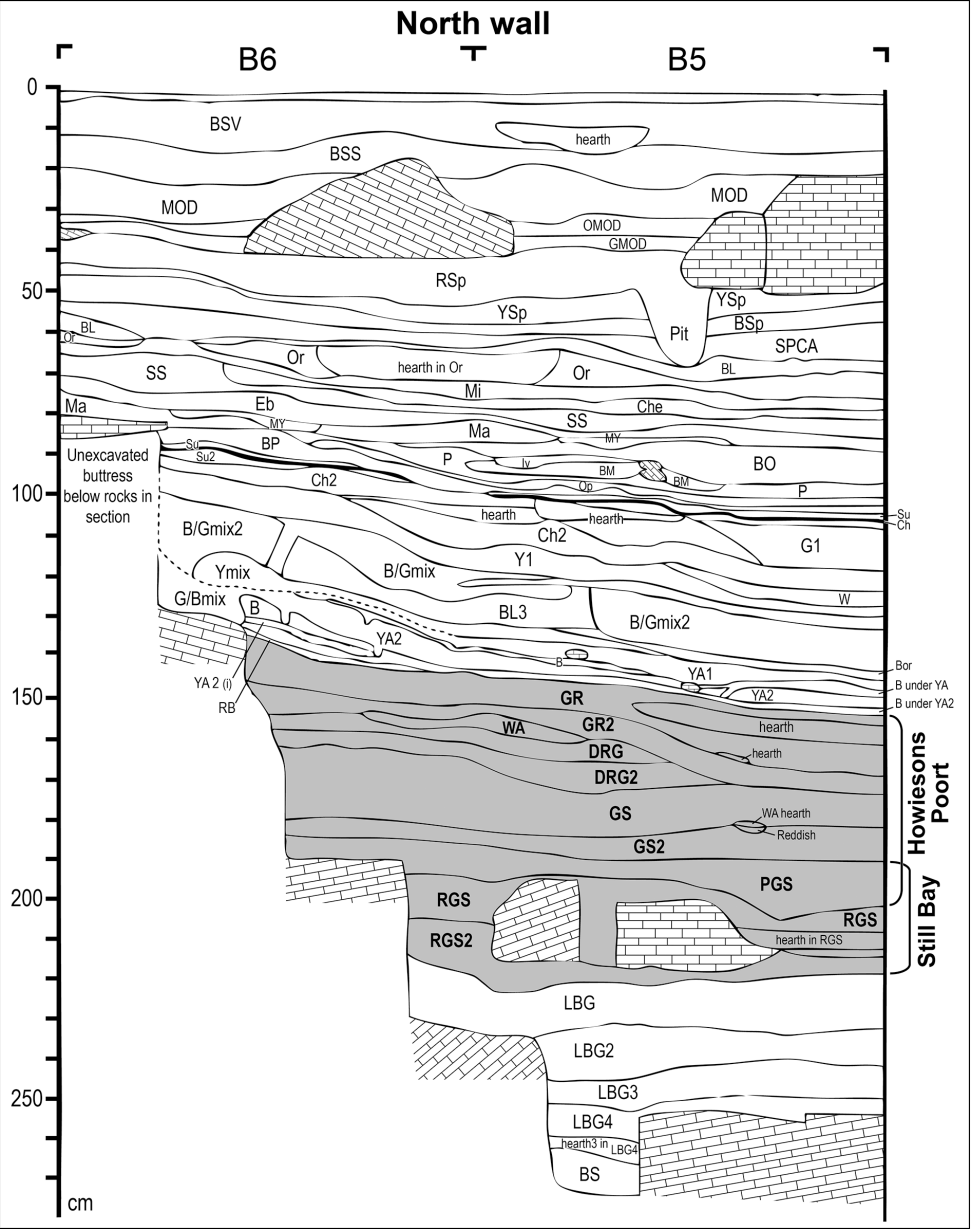
Sibudu: Stratigraphy of section in squares B6 and B5. The SB and HP layers are shaded in grey (modified after [25]).

Sibudu (historically called Sibudu Cave; ID no. 2931CA; 29°31′21.5″S, 31°05′09.2″E) is actually a large rock shelter formed in the sandstones and shales of the Natal Group, on a cliff above the Tongati River (also spelled uThongathi) in KwaZulu-Natal. It is located about 40 km from Durban and 15 km from the Indian Ocean. The shelter is 55 m long and about 18 m in breadth, sloping from north to south. The present excavations began in 1998 under the direction of Lyn Wadley (Fig 2). It has a 2.7 m deep MSA sequence spanning from ca. 77,000 to 38,000 years ago (Fig 3), from the pre-Still Bay to final MSA assemblages [30–35]. The deposit is excavated in 50 cm quadrants (a,b,c,d) within each meter square; material is labelled, for example, B5a, B5b, B5c, B5d. Quadrant a is always the NE-facing corner of each square.

The deepest stratigraphic layers are BS, LBG 4, LBG3, LBG2 and LBG, reached only at the base of L. Wadley’s excavation above a rock fall but current excavations by N. Conard developed below this rock fall. They contain an assemblage called pre-Still Bay, as yet unpublished. On top of LBG is RGS, a loose, reddish-grey sand, artificially split in two sublevels (RGS2 and RGS) as is usual at the excavation when natural layers are thicker than 10 cm. These contain the Still Bay artifacts. The Howiesons Poort occupations first occur in layer PGS, a loose, pinkish-grey sand, with a few rock spalls, directly above RGS. Lying on this are GS2 and GS (Grey Sand), GR 2 and GR (Grey Rocky). DRG (and its subdivision DRG2) is a small lens that occurs only in the western part of the excavation. These are the Howiesons Poort layers. There are no sterile layers between the Still Bay and the Howiesons Poort but a chronological hiatus is suggested by the OSL dates. Layer RGS has an age of 70.5 ± 2.4 ka while layer PGS is dated to 64.7 ± 2.3 ka ([8] *contra* [36]).

#### Stratigraphic problems at the contact of SB and HP layers

According to Wadley [29] rock falls must have occurred before and during the deposition of layer PGS causing stratigraphic disturbances. Goldberg et al. [35] note that the Howiesons Poort and lower layers are more massive than the laminated post-Howiesons Poort deposits which contain intact combustion features. The HP deposits are also composed essentially of compacted combustion products, but most of these are no longer in place. Extensive trampling had the effect of homogenizing the units. During the excavation Wadley observed chaotic alignment of artifacts and bone in some places in the contact layers between the HP and the SB, a sure sign of disturbance. Thus HP segments and SB bifacial pieces occur concurrently in PGS and in RGS (Table 1). The 17 bifacial pieces in PGS are of Still Bay morphology and made of dolerite, hornfels and quartzite (Fig D in S1 File). They cannot be confused with the quartz bifacial pieces recovered from HP layers, which are statistically different in length and breadth from the Still Bay points of layer RGS [37]. In PGS, GS and GR blades, cores and backed pieces are like those reported at Rose Cottage and at Klasies River Cave 1A [20,21]. However 11 backed pieces and 9 HP style blades occur in RGS indicating vertical displacement of pieces downward while upward displacement is indicated by the 17 PGS bifacial pieces (Table 1).

**Table 1:** Stratigraphic distribution of SB and HP lithic markers^1^.

Layers	Blade and bladelet cores	Blades with HP features in Still Bay layers	Bifacial pieces of Still Bay morphology	Backed Pieces
PGS	22	not applicable	17	120
RGS	0	4 (16)	46	9
RGS2	0	5 (35)	14	2

^1^Eleven backed pieces and nine HP style blades, identified by comparison with the HP debitage in the upper layers GS and GR and the HP debitage of Rose Cottage and Klasies River Cave 1A [20,21] are present in SB layers. The numbers in parenthesis is the total number of blades in the SB layers. DRG is a small assemblage with 14 backed pieces and is not included here.

Table 1 shows that there are intrusive elements in layer PGS. This mixing affects all four squares in our sample. The backed tools are very distinctive and we can confidently assign the PGS backed pieces, the blade cores and the blades to the HP. However we excluded from analysis all retouched pieces other than backed in PGS because we cannot assign tools such as scaled pieces and denticulates to one of the two assemblages. The PGS bifacial points have been assigned to the SB.

#### The Still Bay assemblages from layers RGS/RGS2 of Sibudu

*Assemblage composition*: The assemblage composition is provided in Table 2. Since they show no significant technical or typological differences, levels RGS and RGS2 are treated as a single layer. The 17 bifacial points of PGS are included in the total of RGS.

**Table 2:** Sibudu. Still Bay assemblage composition.

Categories	N
Bifacial pieces and fragments	77
Tools on flakes (including unifacial points)	68
Flakes from bifacial shaping	2169
Debitage flakes and blades (excluding HP blades)	45
Cores and core fragments	4
Total	2363

**Fig 4:**
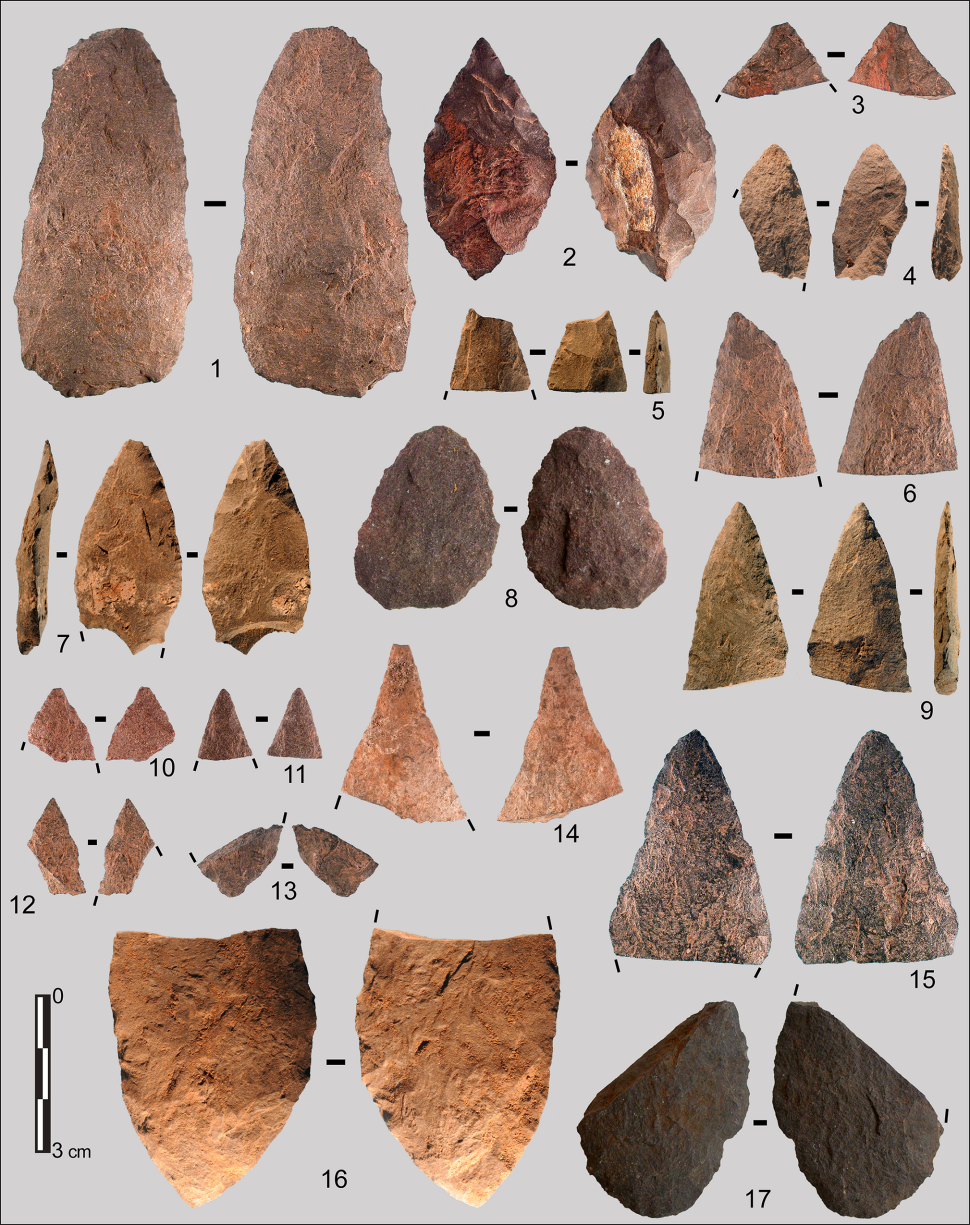
Sibudu, Still Bay: Bifacial pieces and fragments of bifacial pieces. 1, 4–7, 9, 12–17: dolerite; 2, 3: hornfels; 8, 10, 11: quartzite. Layer, square, cat. number: (1) RGS, B6a, PV2; (2) RGS, B6a, 11; (3) RGS, B6a, 28; (4) RGS, C5d, L22; (5) RGS, C5a, L20; (6) RGS2, B5d, 16; (7) RGS2, B4b, L10; (8) PGS, C6c, P5; (9) RGS, B4a, L6; (10) RGS, B6a, 20; (11) RGS, B5c, 23; (12) RGS, B6a, 29; (13) RGS, B5b, 34; (14) HinRGS, B5a, 21; (15) RGS, B5c, 17; (16) RGS, C5c, L12; (17) PGS, C6c, P1.

The SB assemblage is overwhelmingly dominated by shaping byproducts. Bifacially shaped implements, mainly points, were clearly the primary objectives of lithic production (Fig 4)(Figs A-D in S1 File). Very few cores (actually undiagnostic cores or core fragments) were recovered in the SB. Some flakes (N = 4) and blades/elongated flakes (N = 41)(Table A in S2 File) that could not result from bifacial shaping were also identified (Fig E in S1 File). These blades and elongated flakes are rather short, highly variable in shape, mostly with large platforms. They were produced on single platform cores, sometimes partially prepared with cresting. Platforms were infrequently prepared: faceting occurs in 18.5% of the cases (5/27) and internal hard hammer percussion was used. In contrast HP blades have a high proportion of trimming of the core edge (51%) and were made by marginal percussion. The independent production of flakes and blades in the Sibudu Still Bay is minor and has nothing in common with HP highly standardized blade production [20,21]. Thus the following sections will focus on bifacial implements.

*Raw material procurement*: Dolerite, hornfels, quartz and quartzite were available to the Sibudu craftsmen in an area less than 20 km from the site, in primary or derived position. The nearest dolerite source, 200 m away from the site, is a Jurassic dike but many outcrops of dolerite sills are available in a close radius. However sourcing of dolerite artifacts from Sibudu is almost impossible due to chemical similarities across large regions [38]. Survey of the present Tongati riverbank deposits by H. Kempson showed that quartz, quartzite and dolerite cobbles are available but no hornfels was found. The closest primary outcrops of hornfels actually identified is located within 15 km of Sibudu. In the Sibudu SB layers dolerite, hornfels and to a lesser extent, quartz (milky and crystal quartz) and quartzite were collected and used. Other materials (chert, volcanic tuff, sandstone) are rare. A small number of shaping byproducts bear cortical or natural surfaces (Table B in S2 File) and for both dolerite and hornfels different types of cortex were observed (fresh cortex, alluvial cortex or natural surfaces) suggesting that primary and secondary outcrops were exploited.

Dolerite is predominant within SB shaping flakes but it is less frequent in bifacial pieces than in shaping flakes (Table 3). Hornfels is equally represented within flakes and bifacial pieces whereas frequency of quartzite shaping flake is low by comparison with bifacial pieces. Differential transport of artifacts in a finished or semi-finished state may explain this discrepancy (cf. section on “Production and discard”).

**Table 3:** Sibudu Still Bay. Frequencies of raw material types in shaping flakes and in bifacial pieces.

Layer	Dolerite %	Hornfels %	Quartzite %	Sandstone %	Quartz %	Other %	Indet %
RGS, RGS2 shaping flakes (N = 2169)	63.7	27.2	4.1	2.2	1.7	0.2	0.9
PGS, RGS, RGS2 bifacial pieces (N = 77)	49.4	27.3	22.1	0	1.3	0	0

*Bifacial shaping*: Most, but not all, of the bifacial material from Sibudu SB are foliate points (Fig 4)(Figs A-D in S1 File). Three pieces are bifacial tools that do not present the characteristics of bifacial points (thinness, small size) and cannot be confused with broken reworked points. Two of these are thick bifacial tools with distal and/or lateral cutting edges (Fig C: 1, 4 in S1 File and Fig F: 1, 3 in S1 File). The third, with a transverse cortical base, is also a bifacial tool with a lateral convex scraper-like cutting edge (Fig C: 3 in S1 File and Fig F: 2 in S1 File).

The assemblage is clearly dominated by fragments (8.4%) and especially by distal fragments (tips and distal fragments > 3cm) which constitute more than half of the fragments (Table 4). The length distribution shows that the major part of the assemblage consists of objects less than 35 mm in length (Fig G in S1 File).

**Table 4:** Whole and broken Still Bay points and bifacial pieces.

Counts of Still Bay points and bifacial pieces		
**Fragment types** ^1^	N	%
Tip (<3 cm)	31	
Distal (> 3 cm)	5	
Distal-middle	6	
Midsection	4	
Proximal-middle	6	
Base	10	
Lateral fragment	3	
**Subtotal of fragments**	**65**	**84.4**
Complete or almost complete	5	
Missing the tip only	1	
**Subtotal of complete or almost complete**	6	**7.8**
Tip flakes	6	**7.8**
**Total**	**77**	**100**

^1^Fragment types are as in [27].

The points are quite variable in shape and size. Maximum width can be determined only for 20 pieces and ranges from 14 to 40 mm without a patterned distribution. The shape of the base varies (Table C in S2 File) but broad bases (Fig 4: 17) and wide arched bases (Fig 4: 2, 16) are common. The tip shape is mostly V-shaped but subtle variations are obvious. The tip can be perfectly pointed (Fig 4: 12) or slightly rounded (Fig 4: 4) and on some tips, one or both edges can be concave (Fig 4: 3, 10, 14).

The classification of bifacial pieces according to phases of manufacture shows that with rare exceptions they were most often abandoned at the stage of finished product or as recycled (Table 5).

**Table 5:** Phases of manufacture of the Sibudu Still Bay points^1^.

Phases of manufacture	N
1. Initial shaping	1
2. Advanced shaping	1
3. Finished products	40
4. Recycled, modified	12
Unattributed	4
Total	58

^1^The classification of bifacial pieces according to their phase of manufacture follows [27]. Pieces of the 2009 excavation (N = 19) are not considered here.

In these stages, the knapping scars of the earlier phase of bifacial shaping are often obliterated by the later knapping scars (sharpening, resharpening and recycling / reworking). For these reasons, it is difficult to describe in detail the manufacturing sequence if we use only the pieces themselves. To understand the shaping process, we studied the shaping flakes. Debitage is underrepresented in this assemblage so almost all of the flakes are byproducts of bifacial shaping. We distinguish three types of flakes that reflect the principal changes of the state of the shaped tool, from the unworked raw material volume to the finished tool, passing through the roughout and preform phases. The three phases are: initial blank shaping, advanced shaping and final shaping [26]. The three flake types are illustrated in Figs H-J in S1 File and their constituting attributes are listed in Table D in S2 File. Further details on shaping flake classification can be found in the S3 File.

#### Initial shaping (Type 1 flakes)

Type 1 shaping flakes are few (9.7%, Fig H in S1 File, Table E in S2 File) in agreement with the absence of bifacial roughout (only one possible quartzite basal fragment; Fig B: 22 in S1 File). The rarity of completely cortical shaping flakes (1.34% of the total) suggests that the initial shaping was done outside the site and that roughouts were brought in. The frequency of Type 1 flakes with cortex on the dorsal face (38.7%, Table F in S2 File) or on the platform (31.5%, Table G in S2 File) shows that important portions of natural surfaces remained on the roughouts. In certain cases the shaping began on flakes with some natural surface. A portion of the ventral face of a flake was recognized on the wide platform of one of these flakes (Fig H: 2 in S1 File). These Type 1 flakes are bigger than those of the following phases (Table H in S2 File) but also of more variable dimension because the shaped surfaces are still irregular at this stage. Wide platforms unprepared (i.e. plain, cortical/natural), dominate (62.2%; Table I in S2 File), and the overhang is never abraded (Table J in S2 File) indicating internal percussion with a hard hammerstone. Even if shaping began outside the site, low frequency of initial shaping flakes (relative to advanced and final shaping flakes; Table E in S2 File) suggest that this first phase of shaping was rather short. Probably the artisan selected volumes of raw material close to the final shape of the biface.

#### Advanced and final shaping (Type 2 and 3)

Type 2 flakes are the most abundant (61.8%; Table E in S2 File) and share some features with Type 3 flakes (final shaping flakes). Their average size is identical (Table H in S2 File) and the cortical residues are equally rare (Tables F and G in S2 File). The presence of portions of natural surfaces on some distal fragments of hornfels bifacial pieces indicates that the shaping was of limited extent when the expected final volume was close to the original volume of raw material.

The final shaping flakes (Type 3) were more frequently and more intensively prepared: almost 40% show an abrasion of the platform edge (Table J in S2 File) and 47.7% have a faceted platform (Table G in S2 File). The fully marginal percussion gesture, in addition to preparation, induced a high frequency of narrow linear platforms (81.5%; Table I in S2 File) on these final shaping flakes. Type 2 flakes with curved profile are 38.5%, (Table K in S2 File) much less than those of Type 3 (67.8%). It means that in the last shaping stage the bifacial pieces have acquired, at least partially, an asymmetric section of convex-plan type (or its variants) and that the shaping / sharpening takes place especially on the convex face.

Such a structure and hierarchy between faces (Fig K in S1 File) is usual on bifacial tools because it allows several resharpenings without any change or alteration of the structure [39,40]. This is also why scrapers are generally retouched on the dorsal surface of the flake rather than on ventral. Analysis of bifacial pieces themselves shows however that this hierarchical organization is not present along all edges (Table L in S2 File). Indeed, almost all distal fragments have this kind of hierarchical organization, not present on proximal fragments (Fig L in S1 File). In other words, one observes a clear opposition between proximal parts (symmetrical lenticular cross sections, faces not organized hierarchically, irregular edge delineation in profile) and distal parts (asymmetrical cross sections, faces hierarchically arranged, regular “scraper like” cutting edges) of the bifacial points.

*Bifacial points*: *long-lived tools through resharpening and reworking*: Many observations suggest that the bifacial points at Sibudu were maintained to ensure long-term use. Detailed analysis of removal organization and chronology was done even on the smallest bifacial fragments, with the help of diacritic diagrams [41] proving that bifacial points were reduced through intensive resharpening and reworking.

**Fig 5:**
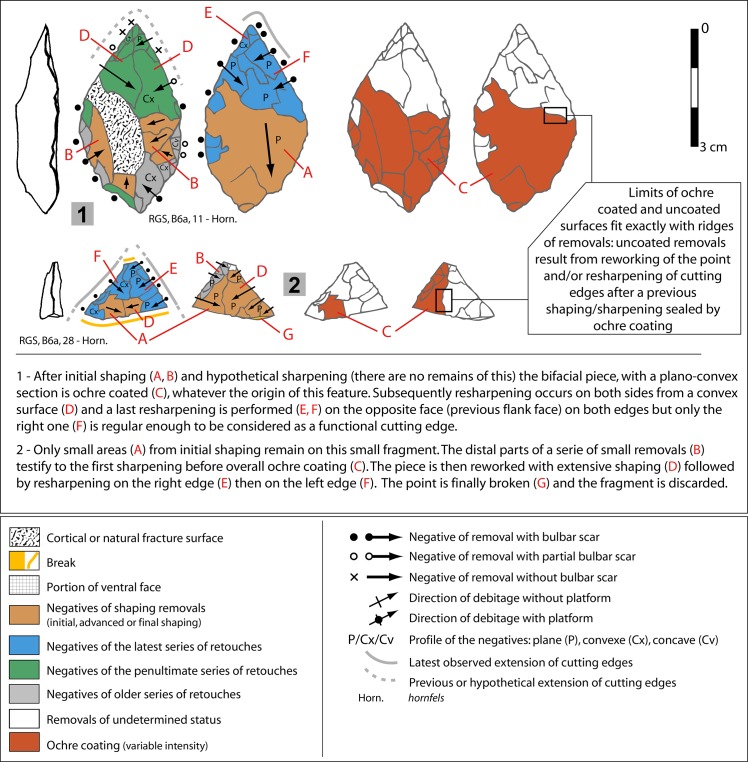
Sibudu, Still Bay: Bifacial pieces with ochre coating. Ochre coating was observed on two bifacial pieces, one complete and a fragment. These pieces where knapped before and after ochre was applied. This feature helped in reconstructing the shaping sequence, by showing the succession and superposition of removals in the resharpening of bifacial points. Photos of both pieces are in (Fig 4: 2 and 3).

Two pieces clearly show that shaping and sharpening were discontinuous through time (Fig 5) in agreement with alternation of sharpening and use. These bifacial pieces were first shaped, sharpened and an ochre coating was later applied on knapping scars (the origin of this feature is unknown). Resumption of shaping and/or resharpening afterwards is confirmed by ochre free knapping scars. These tools must have been utilized between the knapping phases.

**Fig 6:**
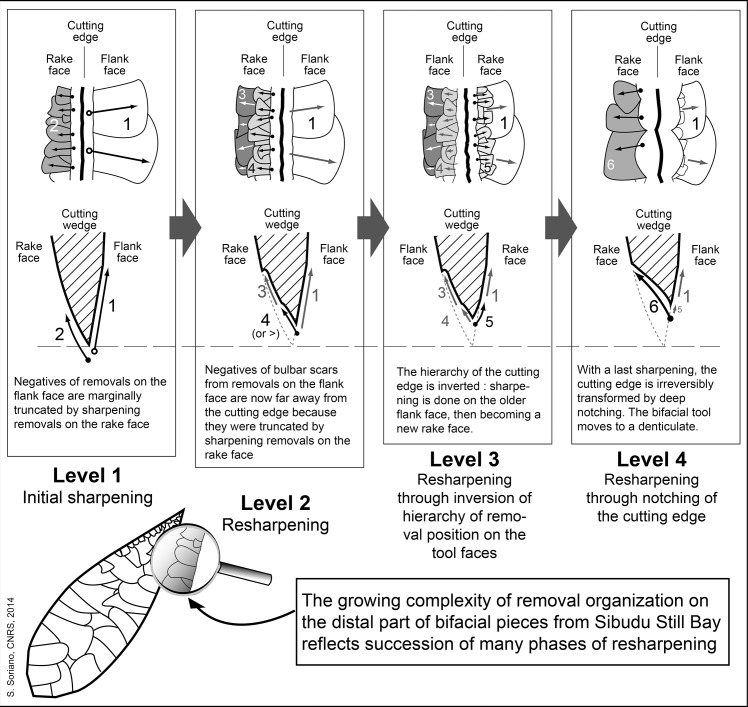
Phases of resharpening in the Sibudu Still Bay points.

Important variations in complexity of removal organization on the distal part of bifacial pieces from Sibudu Still Bay were observed (Fig M in S1 File). These features are patterned in a four level reduction sequence that reflects succession of several phases of resharpening (Fig 6). In fact, successions of sharpening and resharpening of a stone cutting edge in agreement with the geometry of cutting tools (S3 File) induce a growing complexity of knapping scars that can be organized in a *chaîne opératoire* framework. Discarded tips of bifacial pieces suggest that breaking occurred at different moments of this maintenance process.

The high frequency of small bifacial fragments, especially tips, is best explained by tool reworking. On two large fragments the fracture is also clearly associated with attempts to rework the tool. Both show a large removal adjacent to their fracture that created a sharp break in the edge delineation: on (Fig 4: 9), note a large removal adjacent to the fracture on left side, central view, and on (Fig A: 2 in S1 File), note large removal adjacent to the fracture on right side, right view. The breakage of these pieces was a consequence of these deep removals (as one can observe experimentally, [42]). Reworking of the bifacial foliate points sometimes occurred between two stages of resharpening (Fig N in S1 File).

The last level of resharpening is much less represented and was infrequently used. (Fig O in S1 File). Such a process of final sharpening of bifacial tools has been described in Acheulian [43,44] and Mousterian contexts [45]. Some broken bifacial pieces have been retouched with a hard hammer, using percussion that occurs away from the edge of the tool, changing it to a denticulate.

In some cases, the degree of resharpening is difficult to estimate from the completeness of removals on the rake face because their length is diminishing each time a retouch flake is extracted from the flank face. The shorter they are (compared to their width), the more the point was resharpened. In some cases, we can notice that removals on the rake face are highly reduced (Fig N: 2E in S1 File), suggesting the succession of at least two or three episodes of resharpening. It was mostly restricted to the tip, but to go further in the resharpening process, an extended reworking of the tool was necessary.

**Fig 7:**
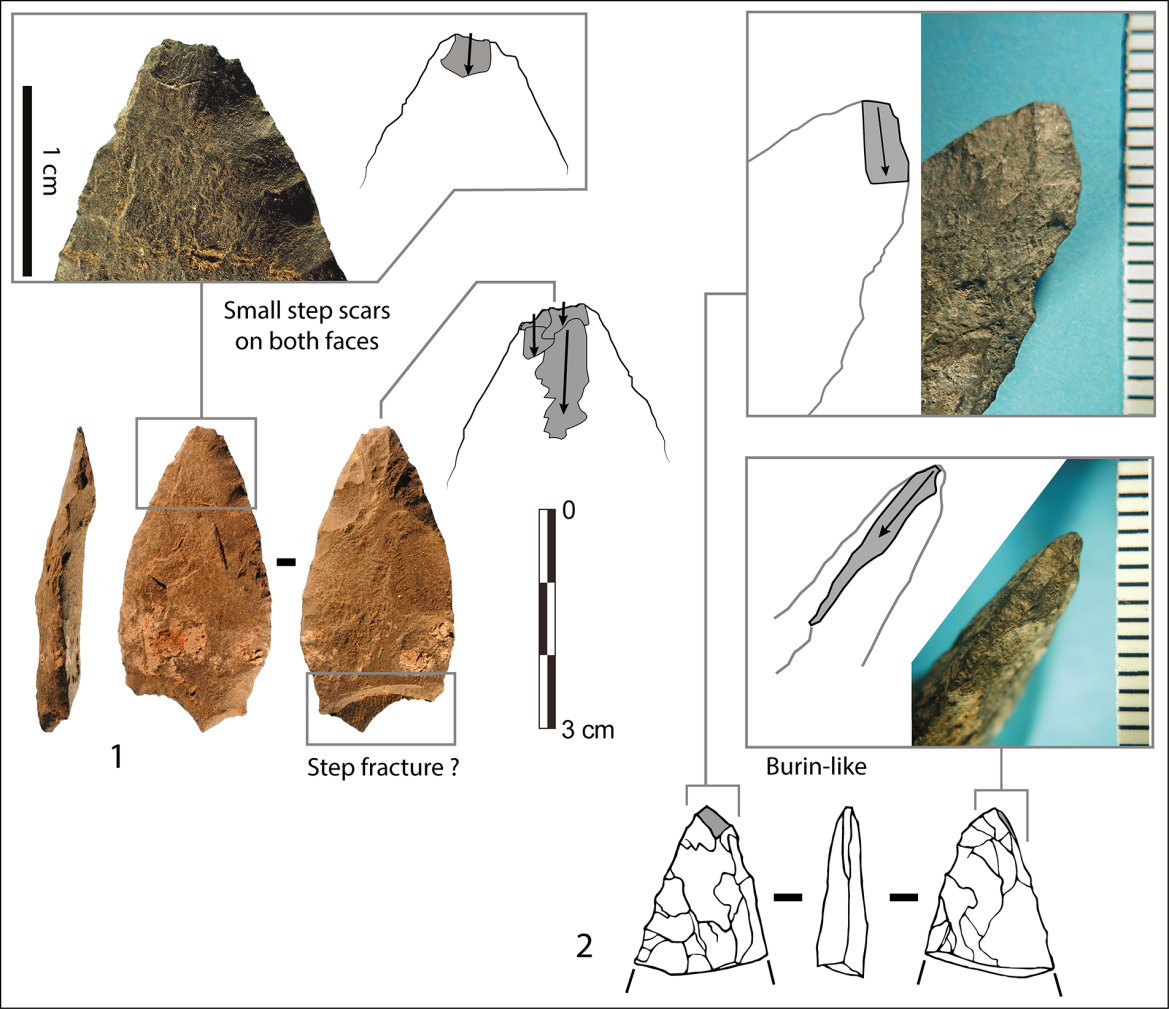
Impact scars on Sibudu points. Layer, square, cat. num., raw material (1) RGS2 B4b no. 10, dolerite; (2) PGS B6a P 15, dolerite.

*Possible functions of the Sibudu bifacial points*: The asymmetrical and hierarchical structure of retouched edges of these points is characteristic of a cutting edge ([46,47]; S3 File) and designed to allow multiple resharpening. Our analysis demonstrates that the bifacial shaping at Sibudu is organized to produce pointed cutting tools, but we cannot prove that each bifacial point was used that way. Retouch restricted to the third distal part of the points is consistent with hafting; however we do not have direct evidence of hafting, with the exception of possible hafting microwear (Fig P in S1 File). The extent of retouched cutting edges appears identical on both edges of the points, suggesting that they may have been hafted axially. These features do not preclude utilization as tips of throwing or thrusting spears. In fact, a few diagnostic impact scars have been observed on Sibudu SB points (Fig 7). Our observations match evidence from previous work [24].

*Knapping technique in the Sibudu Still Bay*: It has been recently proposed that pressure retouch was used at Blombos for the final shaping of about half of phase 3 bifacial pieces made on heat-treated silcrete [28]. Examples from the Upper Paleolithic of Western Europe show that pressure flaking was not necessary for the final shaping of bifacial or unifacial foliate pieces [48,49]. Pressure retouch was only identified for final shaping of some laurel leaf points from the Solutrean [50], sometimes associated with heat treatment [51]. Heat treatment improves the knapping quality of some, but not all, rocks used in making tools. Heat treatment does not improve the knapping quality of crystal quartz [41] and is unlikely to improve the quality of igneous and contact metamorphic rocks, such as dolerite and hornfels used at Sibudu, that have already been heat-treated to very high temperatures by nature. Moreover several technical features suggest that, differently from Blombos, percussion, not pressure, was used for the final shaping of the Sibudu Still Bay points.

Knapping accidents characteristic of the use of percussion are present. Siret fractures occur on 1.5% of initial shaping flakes and 1% of advanced shaping flakes. This is direct evidence of the use of a hammerstone at least for the first phases of the shaping. A small proportion of final shaping flakes (1.3%) has platforms with oversized lips (Fig J: 11, 13 in S1 File) which indicates that the fracture initiated behind the edge where the force was applied (Fig Q in S1 File). In this kind of fracture on a thin edge [52] the flake itself becomes a fragment of the shaped piece with a platform as large as a portion of the dorsal face. Experimentally, this type of accident can be obtained when a marginal percussion (organic or stone hammer) is badly oriented on the thin edge of a shaped piece and/or when the preparation (faceting, abrasion) was oversized. The experimental work of Pelcin [53] shows that flakes with lipped platforms can be produced with a hard hammer by increasing the platform thickness on a core with a low exterior platform angle.

The occasional use of nodules of ochre as hammerstone for the shaping of SB bifacial pieces at Sibudu has been documented [26]. We have also observed a few cases of a double bulb of percussion corresponding to two simultaneous impacts both on advanced and final shaping flakes (Fig I: 5 in S1 File; Fig J: 8 in S1 File). This feature is specific to hard hammer, possibly to a soft stone hammer.

Percussion was also used for the final shaping and the sharpening of bifacial points as suggested by the presence of tip flakes. Tip flakes are overshot retouch flakes at the apex of points (Fig M: 1, 7 and 9 in S1 File). Unlike Villa et al. [27] we now consider that at Sibudu and Blombos these tip flakes result from knapping accidents and not from the intentional removal of the bifacial tip. The extent of their ventral face and their volume indicate the use of percussion since they imply a quantity of energy incompatible with the use of pressure retouch. This is also the case for the 7 tip flakes at Blombos (three of phase 2 and four of phase 3). The repetitive pattern observed in the orientation of fractures on distal fragments of bifacial pieces also indicate that the sharpening / resharpening was made with a percussion technique rather than a pressure technique (Fig R in S1 File **)**. Finally, large platforms observed on final shaping flakes (Fig J in S1 File) could not be obtained regularly with the pressure technique because with this technique the energy is delivered with a compressor through a very narrow surface and narrow platforms are expected.

In conclusion, several lines of evidence point to the percussion technique (first internal then marginal [20]) being used from the initial to the final shaping and sharpening of bifacial SB points at Sibudu.

*Production*, *use and discard of bifacial pieces through time and space*: The mobility of bifacial tools is a major aspect of ongoing discussions about SB toolkit, economical organization and land use [54]. The question of segmentation of bifacial tools production, use and discard through time and space for Sibudu SB has to be examined.

All types of expected shaping by-products have been identified (initial, advanced and final shaping flakes) thus suggesting that knapping occurred on the site. However, less than 10% of the shaping flakes are coming from initial stage of manufacture (Table E in S2 File). This suggest that in many cases shaping began out of the site; what was brought to the site were preforms rather than unmodified or tested volumes of raw material. This interpretation is in agreement with the low frequency of initial and advanced shaping flakes with cortex (Table F in S2 File).

The number of shaping flakes raises the question of the in situ production of bifacial pieces. Is the number of flakes in agreement with the number of bifacial pieces? The ratio of shaping flakes to points is 40, that is 2169 flakes >10 mm to 54 bifacial pieces. Too few bifacial pieces are complete to allow counting of the number of flake scars >10mm on their faces. Recent experiments on manufacture of Solutrean bifacial points do not provide data to answer this question [50], but Newcomer [55] obtained almost 50 shaping flakes per handaxe, including flakes from the initial shaping. So the ratio of shaping flakes to biface we observe at Sibudu seems coherent. Nevertheless, most of the Sibudu bifaces are small fragments, mainly tips. This suggests that the complementary larger fragments have been moved to an unexcavated part of the site or away from the site, before or after retooling. Conversely, biface blanks or preforms produced elsewhere were introduced to the site, utilized and maintained. This is especially the case for quartzite/sandstone, with 22% of bifaces (mostly distal fragments), but only 6.3% of shaping flakes. Numerous quartzite/sandstone pieces were utilized and broken at Sibudu but very few quartzite/sandstone bifaces were made here. Frequencies of final shaping flakes of dolerite (24.3%) and hornfels (39.6%) are also different (Table 6). It seems that the final steps of shaping (sharpening, resharpening and maintenance) of hornfels bifacial points were more frequently performed than on dolerite points implying that dolerite edges had a longer use-life than those of hornfels.

**Table 6:** Sibudu Still Bay. Frequencies of shaping flake types in dolerite and hornfels.

Layers	Shaping flakes		
**RGS, RGS2**	Initial %	Advanced %	Final %
Dolerite (N = 1381)	9.8	65.8	24.3
Hornfels (N = 591)	4.4	56.0	39.6

We conclude that at Sibudu most of the bifacial tools were broken during utilization, sharpening or resharpening, resulting in numerous discarded tips and tip flakes (Fig 4)(Table 4). The largest tip-missing fragments were reworked (Fig N in S1 File) then discarded or moved away.

#### The Sibudu Still Bay: conclusions

Our technological analysis has allowed us: (a) to attest that stone knapping was almost completely oriented toward the production of bifacial foliate points of Still Bay type while debitage is less important.; (b) to reconstruct the production sequence of these points; (c) to prove that the points were produced by direct percussion by hard hammer, followed by thinning and retouch by soft stone hammer; (d) to show that an overwhelming majority of the points are finished forms, (e) to suggest that the organization of removals and structure of these points are designed for a primary use as cutting devices in agreement with the structure of modern cutting tools; (f) to argue that a long resharpening process was applied to these tools to ensure their long-life use; (g) to propose that most of the points were broken during their use or during their maintenance; (h) to show that these points were also used as tips of hunting weapons. In this case the point would have been hafted on a long spear shaft, while the points used as knives must have been hafted on short handles. Both our (e) and (h) proposals are consistent with a preliminary residue analysis [24].

#### The Still Bay of Blombos Cave

**Fig 8:**
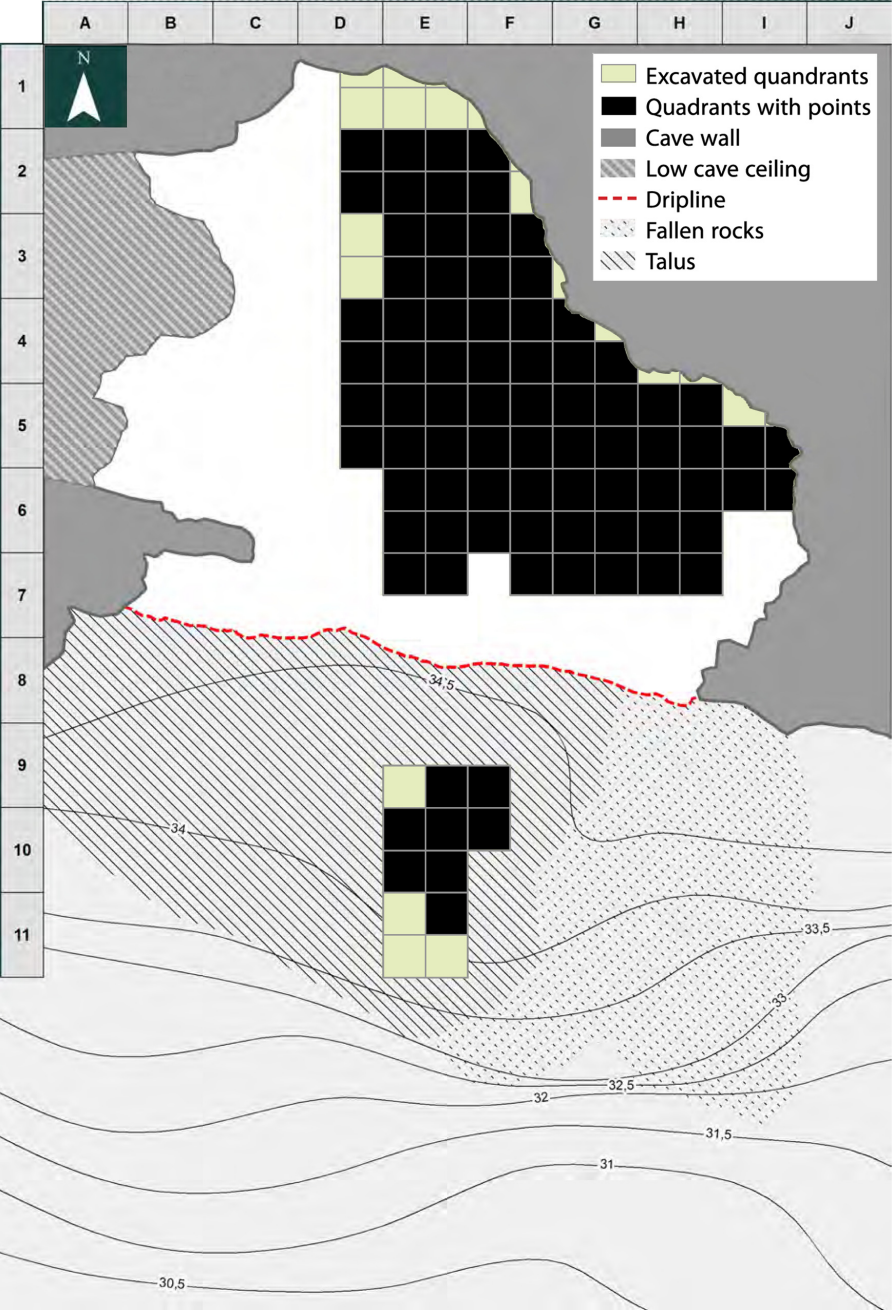
Blombos site plan and spatial distribution of Still Bay points. Each excavated square meter is subdivided in four quadrants (of 50x50 cm). Modified with permission after [58].

Excavations at Blombos Cave (ID no. 32405; 34° 24′ 51″ S, 21° 13′ 04″ E) began in the early 1990s, regular excavations have been going since 1997. The site stratigraphy is about 3 m thick. The MSA levels are divided in 4 phases (from top): M1, upper M2, lower M2 and M3. Above the M1 phase there is a level of sterile eolian sand dated by OSL to 69 ± 5 and 70 ± 5 overlain by Later Stone Age levels dated by ^14^C to 2000–2900 years uncalibrated BP [7,56,57]. The M1 and upper M2 layers are assigned to the Still Bay and are dated between 75 and 72 ka [1]. The Still Bay layers (CA to CF) have been excavated over an area of 23.5 square meters (Fig 8); their total thickness varies from 50 cm on the South section [58] to about 40 cm on the West wall.

#### The sample

The assemblage composition provided in Tables 7 and 8 includes all the Still Bay points and all the retouched pieces, excavated up to and including 2011. There is preliminary information on 45 cores but the debitage which includes several thousand pieces remains to be studied (see Paper organization). Silcrete is the dominant raw material and there may have been two sources: from deposits 30 km north of Blombos and from river valleys which exit into the ocean 20 km east and west of the site [27].

**Table 7:** Blombos Still Bay points and other bifacial pieces.

Blombos Still Bay layers (CA-CF and subunits)	
	**N**
All Still Bay bifacial pieces and fragments	515
Still Bay bifacial points and fragments, by phases of manufacture^1^	489
Bifacial points of non-Still Bay morphology	17
Bifaces (too thick, large or irregular to be classified as points)	3
Indeterminate bifacial fragments (not from points)	9

^1^ Four tip flakes of phase 3 and three tip flakes of phase 2 are included. The number of points is higher than the one published in [27] because we include here all other points found in the 2009–2010 and 2011 excavations.

**Table 8:** Fragments by phases of manufacture^1^ at Blombos and Sibudu.

	Blombos					Sibudu
	Phase 1	Phase 2	Phase 3	Phase 4	Total	Total
Fragment type^2^	N	N	N	N	N	N
Tip (< 3 cm)	**3**	64	56	1	124	31
Distal (>3 cm)	6	13	16	1	36	5
Distal-middle	5	20	11	1	37	6
Midsection	5	14	4	0	23	4
Proximal-middle	5	19	2	1	27	6
Base	11	64	11	1	87	10
Lateral fragment	5	9	2	1	17	3
Tip flakes	0	3	4	0	7	6
***Total fragments***	40	206	106	6	**358**	**71**
Complete or almost complete	23	32	11	12	78	5
Missing the tip only	1	6	4	0	11	1
**Total points**	64	244	121	18	**447**	**77**

^1^ Fragment types as in [27]. Phase 1: initial shaping. Phase 2: advanced shaping. Phase 3: finished products. Phase 4: recycled, modified.

^2^ Non-orientable fragments are excluded.

#### The Blombos Still Bay points

**Fig 9:**
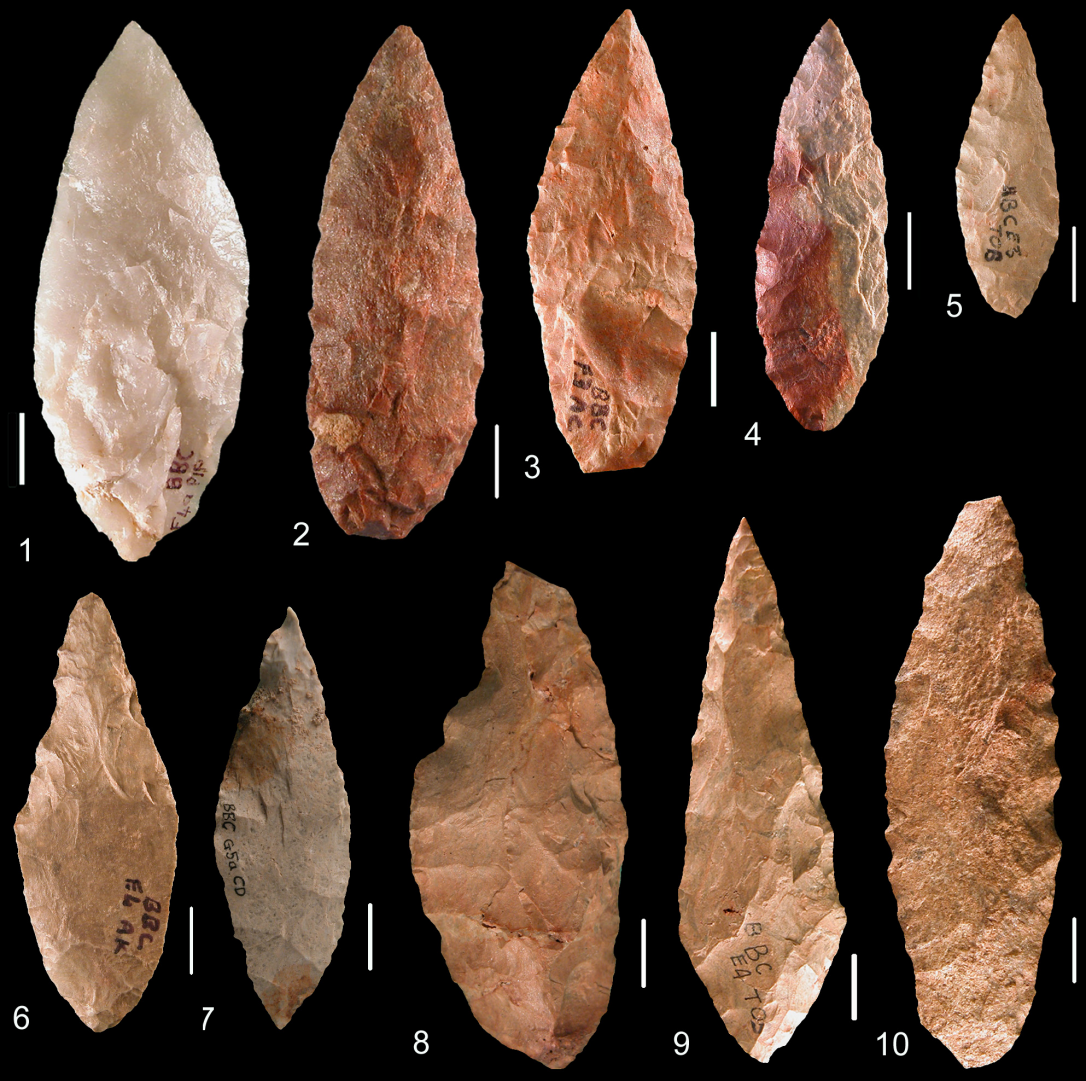
Blombos Still Bay bifacial points. (1–5) Phase 3. (6–10) Phase 4. All of silcrete except no. 1 of quartz. (1) PVN 63 E5b BZ; (2) PVN 7 D4b CD; (3) Museum3 F3 AC; (4) P54 E6a CC; (5) Museum1 E3 TOB; (6) PVN 65 E4 AK; (7) P 71 G5a CD; (8) PVN 140 E3 TOB; (9) PVN 68 E4 TOB; (10) PVN 71 E4 PIP. Scale bars = 1 cm.

**Fig 10:**
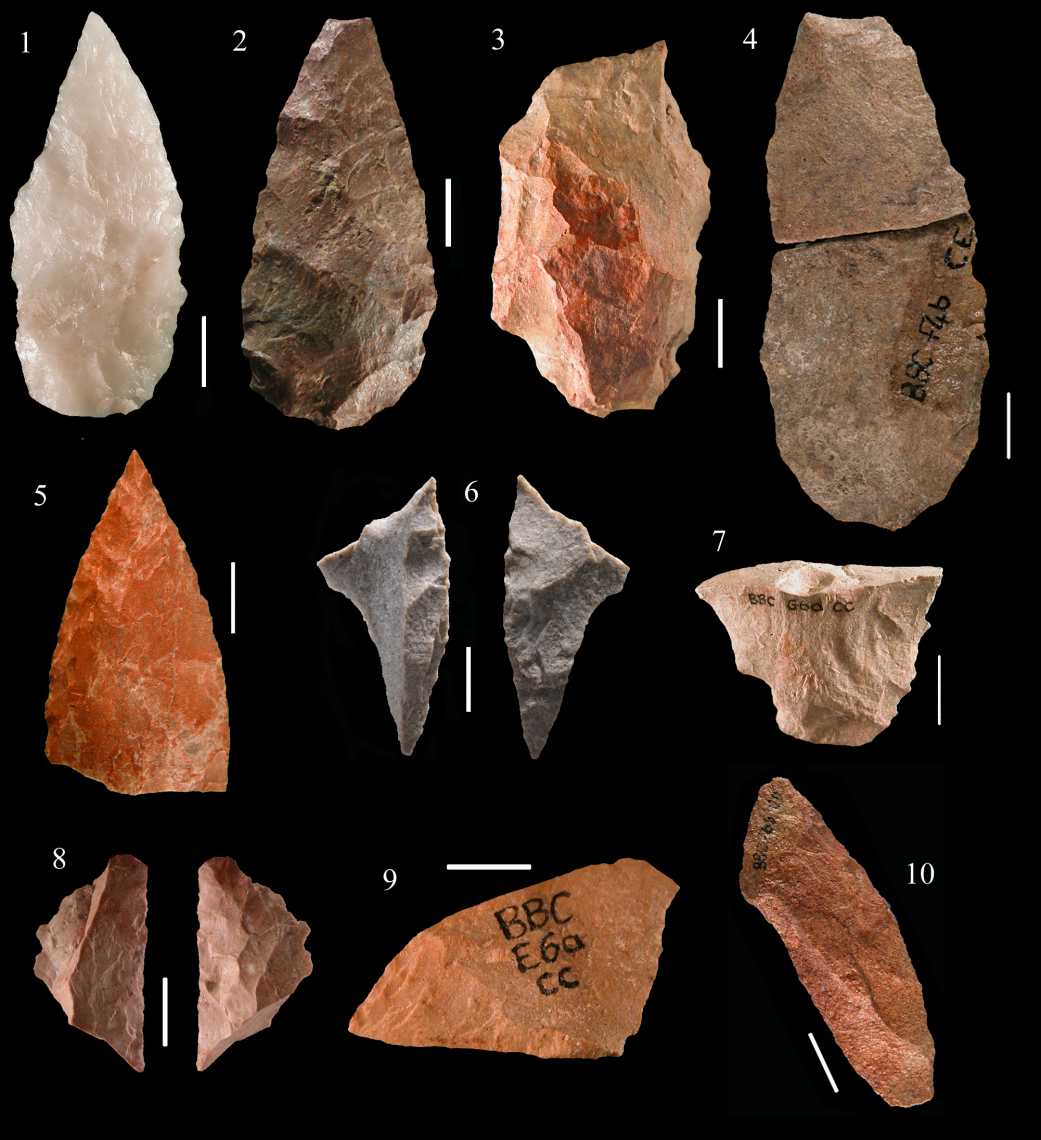
Blombos bifacial points and fragments showing a variety of morphologies. (1–2) PVN 67 and PVN 72, levels PIP and CRE) phase 3 points with a broad base. (3) P 40, CB phase 1; (4) PVN 6, a refit (from layers CE and CD, phase 2b; (5) P68 CDB, phase 3 distal fragment; (6) Lateral fragment, CA 147, phase 3. (7) broken base, P53 CC phase 2a. (8) Tip flake CA 2076 phase 3. (9) Tip flake CA PVN 11 phase 2. (10) Lateral fragment with part of the tip, CD P67, phase 3. This twisted fracture is sometime called “perverse fracture” [27]. Scale bars = 1 cm.

**Fig 11:**
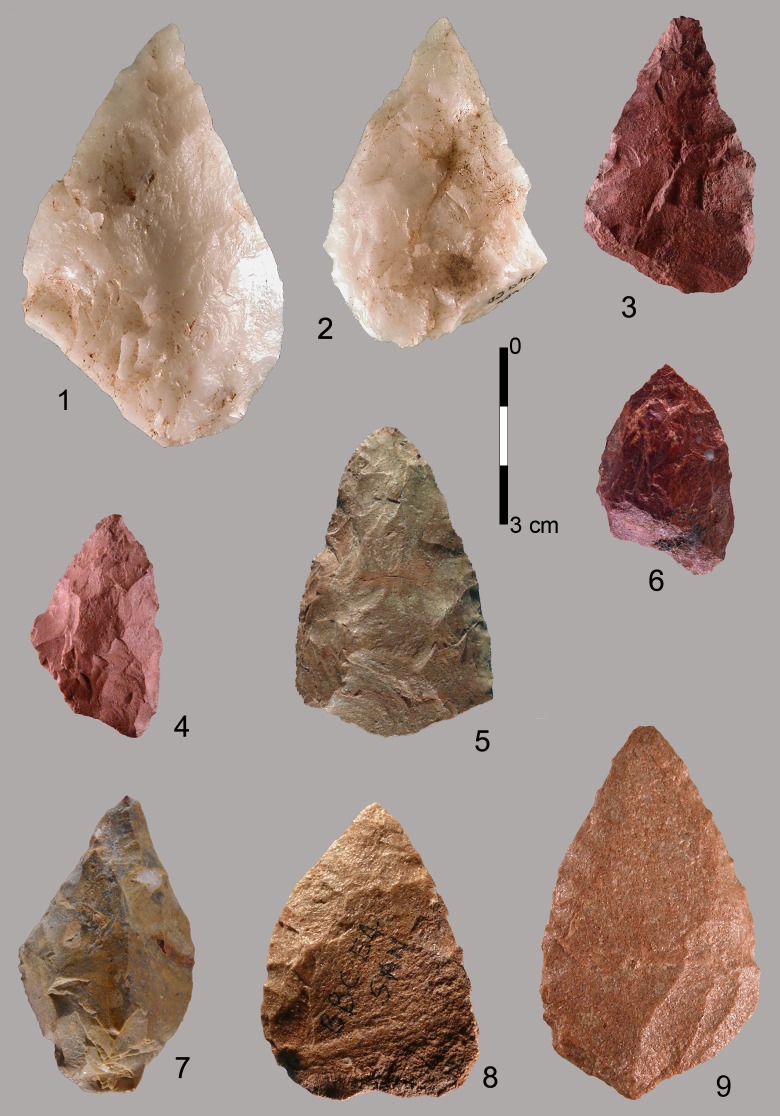
Blombos bifacial points of non-Still Bay morphology. All of silcrete except 1 and 2 of quartz. These points are characterized by an oblique, asymmetrical thick base (1, 2 3, 4, 6) or a very broad or irregular base (5, 7). Two points have only partial and short bifacial retouch (8,9).

At Blombos the typical Still Bay point morphology is characterized by a pointed or narrow elliptical base often with a truncated end and a V-shaped point with straight or curved sides (Fig 9). Broad bases (Fig 10: 1–2) and wide-arched bases are much less common (about 25% of the cases; [27]). At Sibudu instead broad and wide-arched bases are more common while the truncated end is unknown. Points of non-Still Bay morphology are illustrated in (Fig 11).

#### Phases of manufacture of Still Bay points

The four phases of manufacture of the Blombos points and kinds of breakage, described in detail in [27] are provided in Table 8 and compared with the Sibudu points.

Paleoindian bifacial points were also produced in particular reduction sequences and were broken in various stages of manufacture, including during the final phases of pressure flaking along the margins [59]. Reworking of broken projectile point fragment was a common practice at some sites such as the Horner and Agate Basin sites; points became proportionally shorter as a result of reworking [60,61]. Types of manufacturing breaks and rates of production failures have been reported from a number of Folsom, Clovis, later Paleoindian and Archaic sites (e.g. [59]). Johnson [62] reports production rejects of about 84% at a workshop site; thus the high proportions of fragments from Blombos (80%) in all phases of manufacture is not unexpected.

Reworking of the Blombos points by hard hammer scars with some changes in outline (Fig 9: 6–10) is limited to very few cases (18 of 447, i.e. 4.0%). The length of complete points of phase 3 and 4 is practically identical (Fig T in S1 File). This suggests that the phase 3 points represent the desired end product and that pieces were only rarely modified and resharpened.

At Blombos 79% of the bifacial points (phases 1 to 3) were abandoned in the course of production while at Sibudu these unfinished pieces represent less than 5%. Large roughouts present at Blombos (Fig 10: 3–4) are unknown at Sibudu. Nevertheless, the abundance of shaping flakes at Sibudu shows that point making was also a primary activity at the site. The scarcity of broken bifacial pieces from phases 1 and 2 is puzzling but the sample of points comes mainly from 3 square meters (B5, B6 and C5) compared to 21 square meters at Blombos. Uneven intrasite spatial distribution may be an explanation for this discrepancy.

Pressure retouch is one of the most significant signatures of Still Bay points manufacture at Blombos [28]. Our analysis shows that at Sibudu only direct percussion with a soft stone hammer was used for final shaping and retouch of bifacial points.

#### Axial hafting and impact scars at Blombos

Evidence for axial hafting is provided by three kinds of evidence [27]. The first case is a phase 4 point where the distal part has been completely reworked by hard hammer (Fig 9: 6) suggesting that resharpening was done on a point still in the haft. The second case of axial hafting is indicated by a phase 3 point that is patinated in the distal part exposed to direct atmospheric conditions but unpatinated in the proximal part (possibly due to a haft that deteriorated through time). The third kind of evidence is provided by impact scars on the base of two phase 3 points (a step fracture and two burin-like scars; Fig U in S1 File). This kind of break by countershock in the haft occurs on Paleoindian bifacial point and has been reproduced experimentally on replicas of Paleoindian and Solutrean shouldered points [63].

Impact scars on the Blombos points have been identified based on comparisons with 160 Paleoindian points from four bison kill sites and five kill and residential sites in Colorado and Wyoming and 55 replicates of Solutrean shouldered and unifacial points mounted as spear-heads or arrow-heads and shot into adult cattle, from the experimental series of Geneste and Plisson [64]. The frequency of impact scars at Blombos (13.4%, i.e. 11 of 82 phase 3 points, complete or with preserved distal ends) is comparable to those of other MSA and Middle Paleolithic sites [63,65].

The asymmetry observed on the Sibudu points does not occur on the Blombos points; this suggests that the Blombos points were not designed as knives although we do not exclude their secondary use as knives, if hafted on short handles.

There is no doubt that point manufacture was a primary activity at the site and that the production sequence began using large blanks brought to the site. There is, however, ample evidence that the site was not just a workshop. A variety of activities took place at the site as indicated by large numbers of beads made from *Nassarius kraussianus* shells, bone tools and hundreds of pieces of ochre including seven engraved pieces [5,6,57,66–70], numerous combustion features [58], and many lithic tools other than points (Table 9; Fig V in S1 File). Three deciduous upper molars and one deciduous upper incisor come from different Still Bay layers (CB/CC, CC, CD and CF; [71]); thus both children and adults lived at the site.

**Table 9:** Formal tools other than bifacial pieces at Blombos and Sibudu.

Formal tools other than bifacial pieces	Blombos Still Bay layers^1^		Sibudu RGS and RGS2	
	N	%	N	%
Unifacial points	13	3.5	2	3.8
End scrapers	51	13.6	1	1.9
Side scrapers	77	20.5	4	7.5
Convergent and déjeté scrapers	13	3.5	2	3.8
Scaled pieces	1	0.3	9	17.0
Notches and denticulates	32	8.5	2	3.8
Retouched and utilized pieces	126	33.6	11	20.8
Other tools	3	0.8	2	3.8
Tool fragments	59	15.7	20	37.7
**Total**	**375**	**100.0**	**53**	**100.0**

^1^At Blombos end scrapers includes 21 circular scrapers and 30 end scrapers on thin flakes. Side scrapers include 9 bifacial scrapers; retouched and utilized pieces are on flakes (113) on blades (5) and on chunks (8).

#### Formal tools other than bifacial pieces

Table 9 provides frequencies of tools other than points at Blombos in comparison with Sibudu.

**Fig 12:**
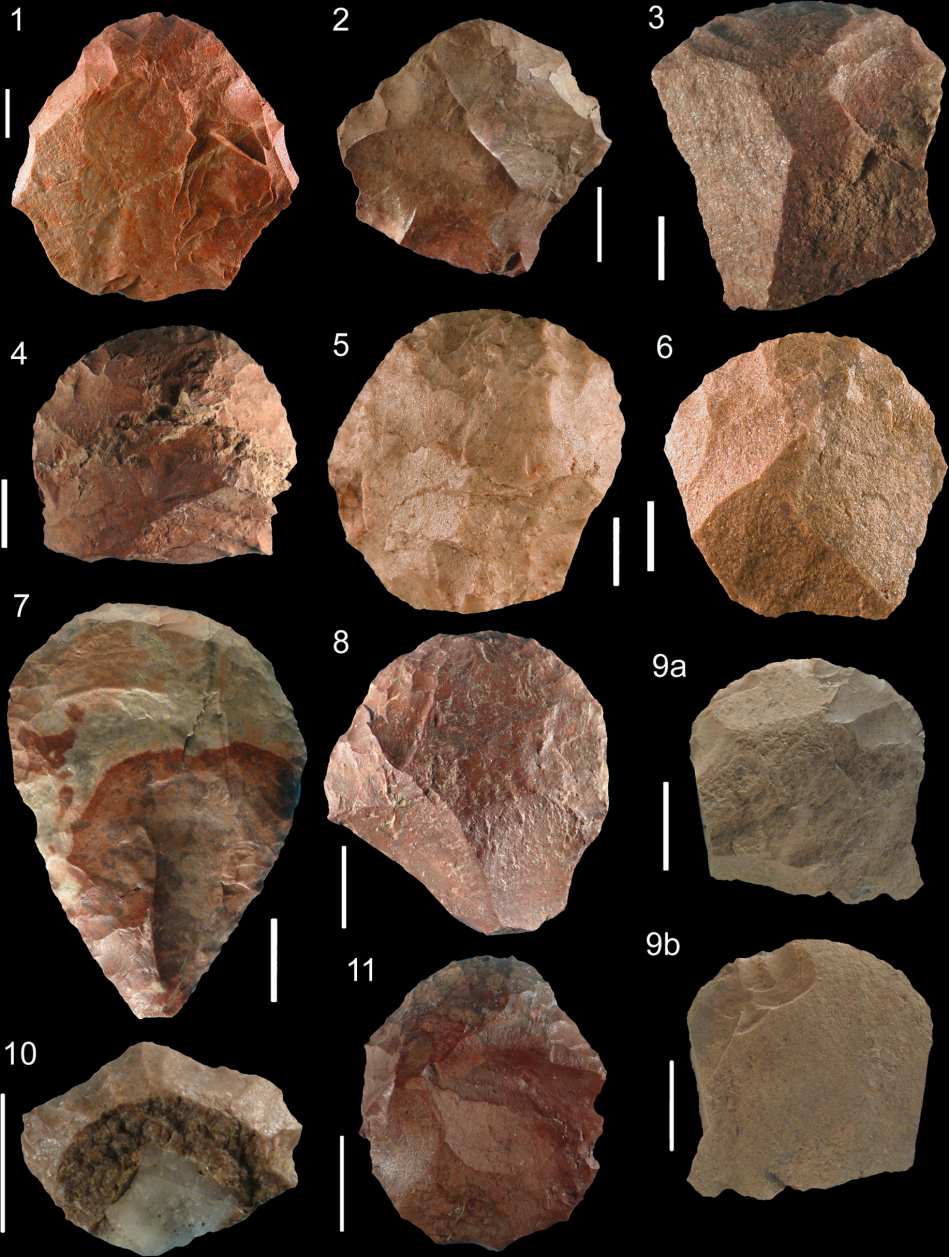
Blombos Still Bay, circular scrapers. All of silcrete. (1, 3, 4, 5) CC nos. 1, 32, 47, 49. (2) CAR no. 6. (6) CD no. 39. (7) CFA-CDB no. 8. (8) CE no. 17; (9a, b) CAB no. 53; (10) CB no. 18. (11) CD-CE no. 56. Scale bars = 1 cm.

There are typological differences in small tools between the two assemblages. Scaled pieces, relatively frequent at Sibudu (17%; Fig S in S1 File) are very rare at Blombos whereas end scrapers and especially circular scrapers, very characteristic of the Blombos toolkit (Fig 12) are much less represented at Sibudu.

#### Cores

Preliminary analysis of 45 cores indicates that cores were not prepared and have short sequences of removals for the production of flakes and some laminar flakes (Fig W in S1 File). The debitage has not yet been studied (see Paper organization) thus we do not know the proportions of shaping versus debitage flakes (i.e. flakes from cores). However the number of cores clearly shows the presence of an independent sequence of reduction. In contrast, at Sibudu, debitage is a minor component of the assemblage and most of retouched tools are manufactured on shaping by-products.

### Comparison of the Still Bay at Sibudu and Blombos

The SB assemblage from Sibudu is peculiar because its lithic industry is almost entirely produced through bifacial shaping while debitage is less important. McCall and Thomas [54] have emphasized the “*increasingly popular interpretation of SB points as specialized weapons*”. In fact, our technological analysis of SB points from Sibudu identify impact scars, but demonstrate that these tools were primarily designed as cutting devices optimized for long-life use through resharpening cycles.

Throughout this analysis we have stressed differences between the Sibudu and Blombos assemblages, that may be related to the kind of occupation (differences in the phase representation and in the resharpening and modification of pieces), differences in raw material (which may explain the non-use of pressure at Sibudu), variations in the details of biface morphology, differences in the use of bifacial pieces, and differences in kinds of retouched tool types. Some of the differences may be related to the distance which separates Sibudu from Blombos, about 1110 km in a straight line. Yet the distances between Klasies and Sibudu (about 850 km) or Klasies and Border Cave (1160 km) or Klasies and Rose Cottage (650 km) are also quite large yet the similarities between the Howiesons Poort assemblages from these sites are much greater (cf. section “The Howiesons Poort”).

At the same time there are also clear similarities between the two Still Bay assemblages from Blombos and Sibudu in the forms of products. Personal ornaments in the form of perforated marine shells (*Nassarius kraussianus*) occur at Blombos [5,68,69]; a few possible beads of marine shells (*Afrolittorina africana*) have also been found at Sibudu [72]. The use of ochre coating of artifacts at Sibudu finds a clear parallel in the presence of ochre coating on the natural surface of two cores, on a phase 1 point and on a dorsal scar of a utilized flake (Fig X in S1 File).

Are these similarities and the similar dates of these and other, albeit less well preserved Still Bay assemblages such as Hollow Rock Shelter [73] and Apollo 11 [74] sufficient criteria to cluster these assemblages into the same lithic tradition [1]? Diepkloof poses a problem. At Diepkloof OSL and TL dates of a stratigraphic unit with a Still Bay assemblage have yielded a significantly older age, 109 ± 10 ka [10] than those previously published for several SB sites [8]. The relatively small lithic assemblage with 73 bifacial pieces comes from two square meters and five subunits approximately 20 cm thick. It has been identified as Still Bay [3,4,75] and is overlain by Howiesons Poort units with a long chronology, from c. 109/105 ka to 52 ± 5 ka. To date the apparent lack of temporal continuity between the Diepkloof Still Bay and the Still Bay of Blombos and Sibudu coupled with differences in technology between Sibudu and Blombos hinder the clustering of these assemblages into homogeneous sets. The debate about the validity of the OSL ages of Diepkloof and Blombos continues to this day [10,36,76,77] and the dating enigma is not yet resolved. We should however make clear that the temporal discontinuity between the Diepkloof SB and similarly named assemblages in the southern and western region of South Africa (Blombos, Sibudu) is supported by the occurrences in the Diepkloof post-Still Bay strata of a group of Early Howiesons Poort assemblages and of a MSA-Jack unit (total thickness of deposits = 45 cm) which have no equivalent in the apparently younger sequences of Sibudu, Klasies River Cave 1A and Rose Cottage.

### The Howiesons Poort

#### Introduction

Our analysis of the HP assemblages at Sibudu focuses on patterns that occur repetitively and are consistently present at other sites, for the purpose of comparisons. Our comparisons are based on data from HP assemblages at Rose Cottage and Klasies of which we have direct knowledge [20,21] and to a minor degree on publications of other sites. We will not discuss the early phases of the HP documented at Diepkloof and concentrate instead on what is often called the “classic” HP dated to MIS 4, after 70 ka. This phase is represented at Diepkloof by the last phases of the Intermediate and Late Howiesons Poort, with mean ages of 65 ±8 and 52 ± 5 ka [10]. At Klipdrift Shelter the HP deposits span the period 65.5 ± 4.8 to 59.4 ± 4.6 ka; similar ages were obtained at Sibudu, with PGS dated to 64.7 ± 1.9 and GR at 61.7 ± 1.5 ka [2,29]. At Klasies River Cave 1A layer 20 (near the base of the sequence) has a OSL date of 64.1 ± 2.6 [8]. At Rose Cottage OSL dates go from 66 ± 4 ka for the base of the HP sequence to 59 ± 4 ka at the top [8,20].

#### Raw material procurement

The conventional view that the HP was associated with long distances of raw material transport [78] has been shown to be incorrect. Raw material procurement, described in the section for the Sibudu Still Bay, is essentially local; dolerite, hornfels, quartz and quartzite were available from an area less than 20 km from the site and were obtained from primary and secondary sources. At Rose Cottage the dominant raw material in HP and post-HP times is opaline, which occurs in the Caledon river gravels 8–10 km from the site, in the form of rolled angular blocks [20,79]. At Klasies River quartz (interpreted as non-local by [80]) and quartzite are also local [21,81]. The sources of silcrete are not known; however the presence of rolled cortex on several silcrete cores and retouched pieces suggests beaches (which are adjacent to the site) or river gravels. At Diepkloof high-quality silcrete comes from sources > 20 km from the site [3]. At Border Cave the main raw material in the HP and younger periods is rhyolite which is the local bedrock. Another, less abundant, raw material is chalcedony which occur as vesicles in the rhyolite and these seem to be most common 40 km from the site [23]. These raw material transfer distances are not significantly different between sites nor are they different from those recorded from Middle Paleolithic sites [13,82].

#### Is the knapping technique used by the HP craftsmen at Sibudu the same as at Rose Cottage and Klasies?

The HP is defined by a debitage almost exclusively oriented to the production of straight blades with thin platform, to be used as such or transformed into backed pieces. The method of blade manufacture at Rose Cottage and Klasies River Cave 1A have been described in detail [20,21]; they are very similar despite strong differences in raw materials. The exclusive use of marginal percussion with soft stone hammer for the production of blades is typical of those two sites, and it has also been described for the Intermediate Howiesons Poort of Diepkloof [3,83].

**Fig 13:**
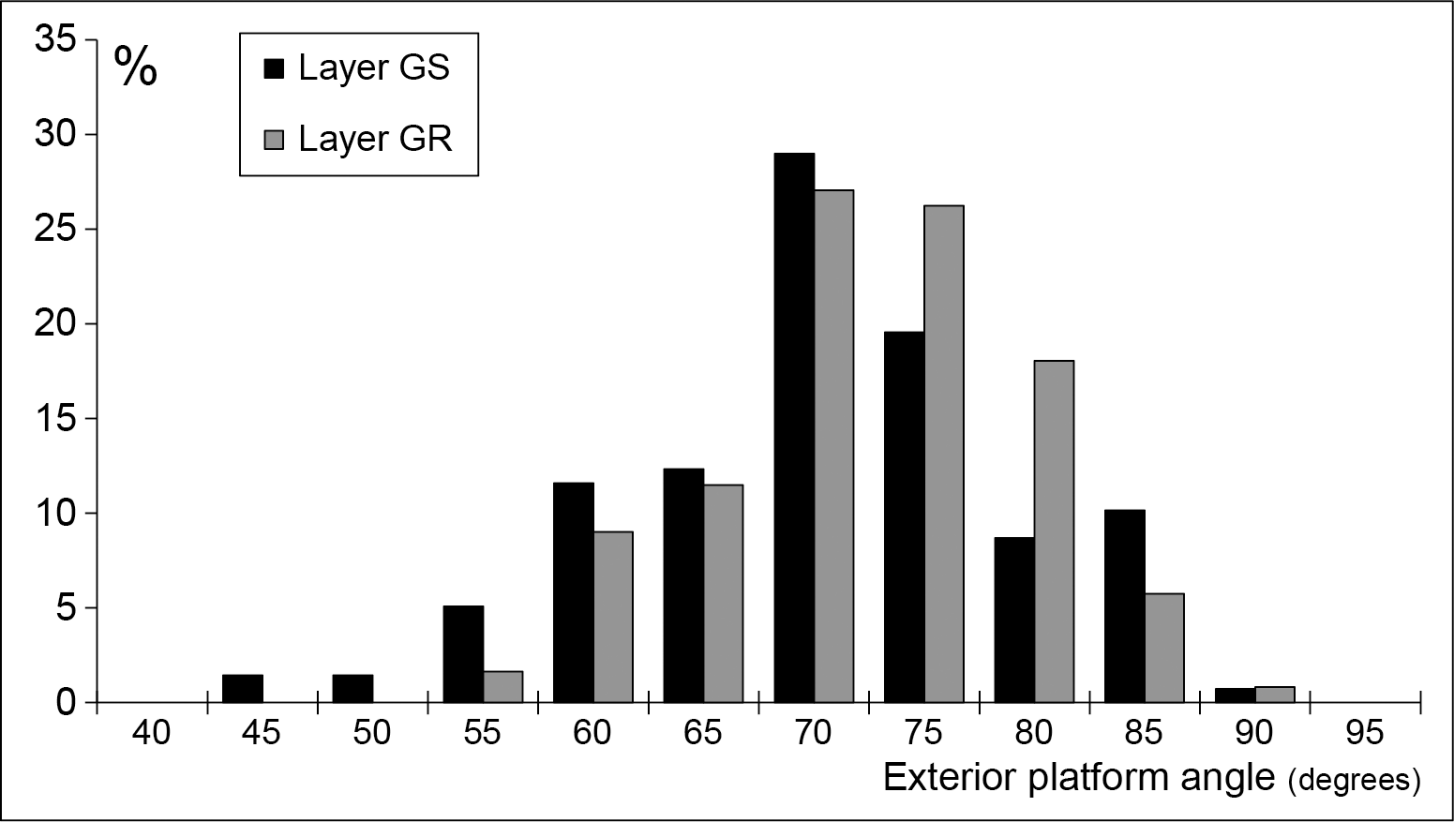
Sibudu HP. Frequency distribution of exterior platform angle on blades in layers GS and GR. All raw materials except quartz.

At Sibudu the evidence appears more complex. According to Pelegrin [84] the optimal values of exterior platform angles for soft stone hammers used in marginal percussion range from 75 to 85°. Then a distribution with a peak around 80° is expected for archaeological samples of blades produced with this knapping technique as we have observed on opaline HP blades from Rose Cottage Cave [20]. At Sibudu, the exterior platform angle distribution is different, centered around 70–75° in GS and GR (Fig 13) and the pattern is almost the same for dolerite and hornfels (Fig A in S4 File).

**Fig 14:**
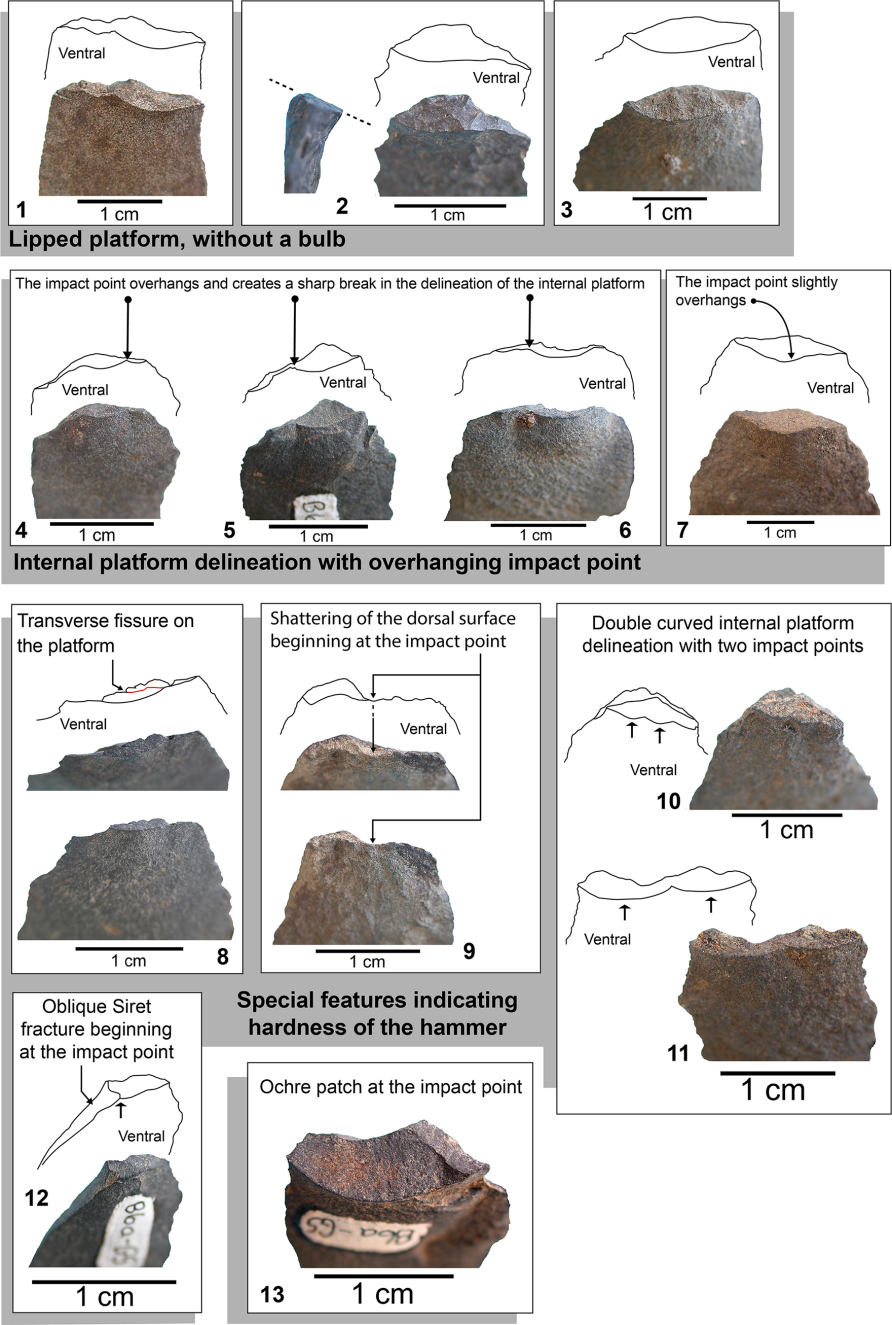
Sibudu HP: Examples of blade platforms showing features resulting from use of a stone percussor.

Does this mean that at Sibudu HP knappers used soft organic (i.e. wood) hammer which is usually associated with lipped platforms and exterior platform angle ranging from low (50^°^) to rather high (80^°^) values [84]? The frequency of lipped platform without a bulb reaching 34% in GR (Fig 14: 1–3)(Table A in S5 File), almost twice the frequency reported for Klasies Lower HP [21] where the use of a soft stone hammer has been demonstrated, would seem to support this hypothesis.

**Fig 15:**
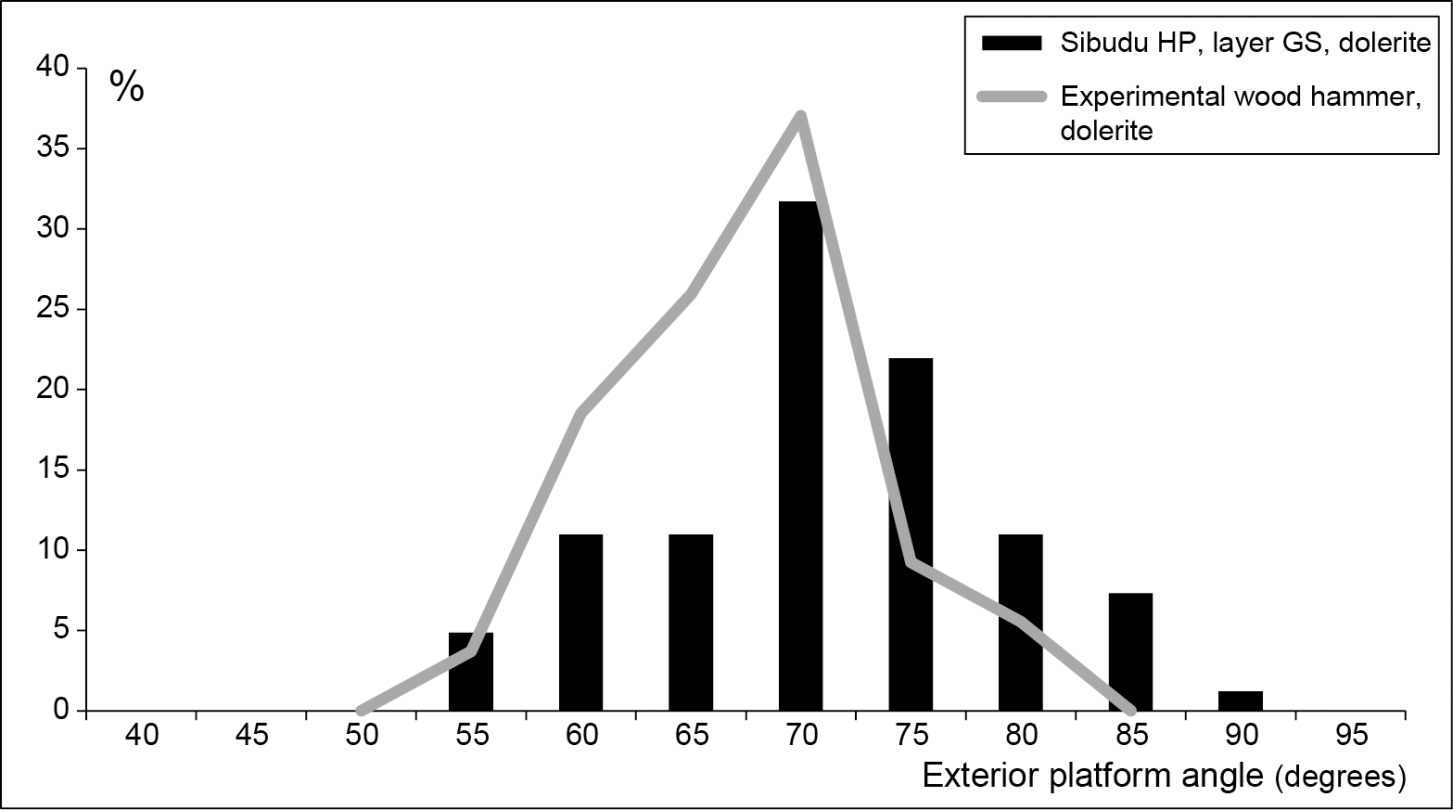
Sibudu HP: Frequency distribution of exterior platform angles for dolerite blades in layer GS (N = 82) and for an experimental sample of dolerite blades made with a wood hammer (N = 54).

However, the distribution of exterior platform angles we obtained for an experimental sample of dolerite blades (N = 55) made by marginal percussion with a wood hammer is different from the Sibudu HP distribution (Fig 15). Peaks are the same in both distributions (70°) but the lower (60–65°) and higher (75–80°) angles have opposite values. Moreover, the highest angle values (85–90°) in the GS sample are absent in the experimental record, as expected with an organic hammer [84].

As at Rose Cottage Cave and Klasies HP, features resulting from use of a stone percussor are noticeable on blade platforms at Sibudu. There are platforms (GS: 7.9%; GR: 11.2%) with a sharp break of their internal delineation (Fig 14: 4–6) indicating that the contact with the hammer was narrow and rather hard. There are also few but undisputable platforms with special features associated with the hardness of the hammer: transverse fissure of the platform (Fig 14: 8), shattering of the dorsal surface at the impact point (Fig 14: 9), double impact point (Fig 14: 10–11) and oblique Siret fracture beginning at the impact point (Fig 14: 12). A blade platform with an ochre patch in layer GS (Fig 14: 13) is indirect evidence of an ochre nodule used as a soft stone hammer [26].

In conclusion, the distribution of exterior platform angles and technical features on blade platforms indicate use of a soft stone hammer for blade making at Sibudu. The high frequency of lipped platforms on blades (Fig 14: 1–3) may be explained by the raw material specificity, especially the dolerite because this igneous rock is softer than flint but more tenacious, so bending initiation of the fracture occurs more commonly than hertzian initiation [52]. Additional experiments (production of dolerite blades with a soft stone hammer) are needed to conclude definitively that a wood hammer was used. At any rate the morphology of blades is much more determined by the knapping gesture-marginal percussion striking near the edge of the platform or internal percussion [20]- than the material of the hammer [85].

#### Are the Sibudu blades and bladelets produced with the same *chaîne opératoire* described for the Klasies HP?

The overall morphology of the Sibudu blades and bladelets (Figs B-C in S4 File) is very similar to those of Klasies [21] and the same pattern is observed in the frequencies of blade width (Fig D in S4 File). The distribution is continuous, there is no width boundary between blades and bladelets.

**Fig 16:**
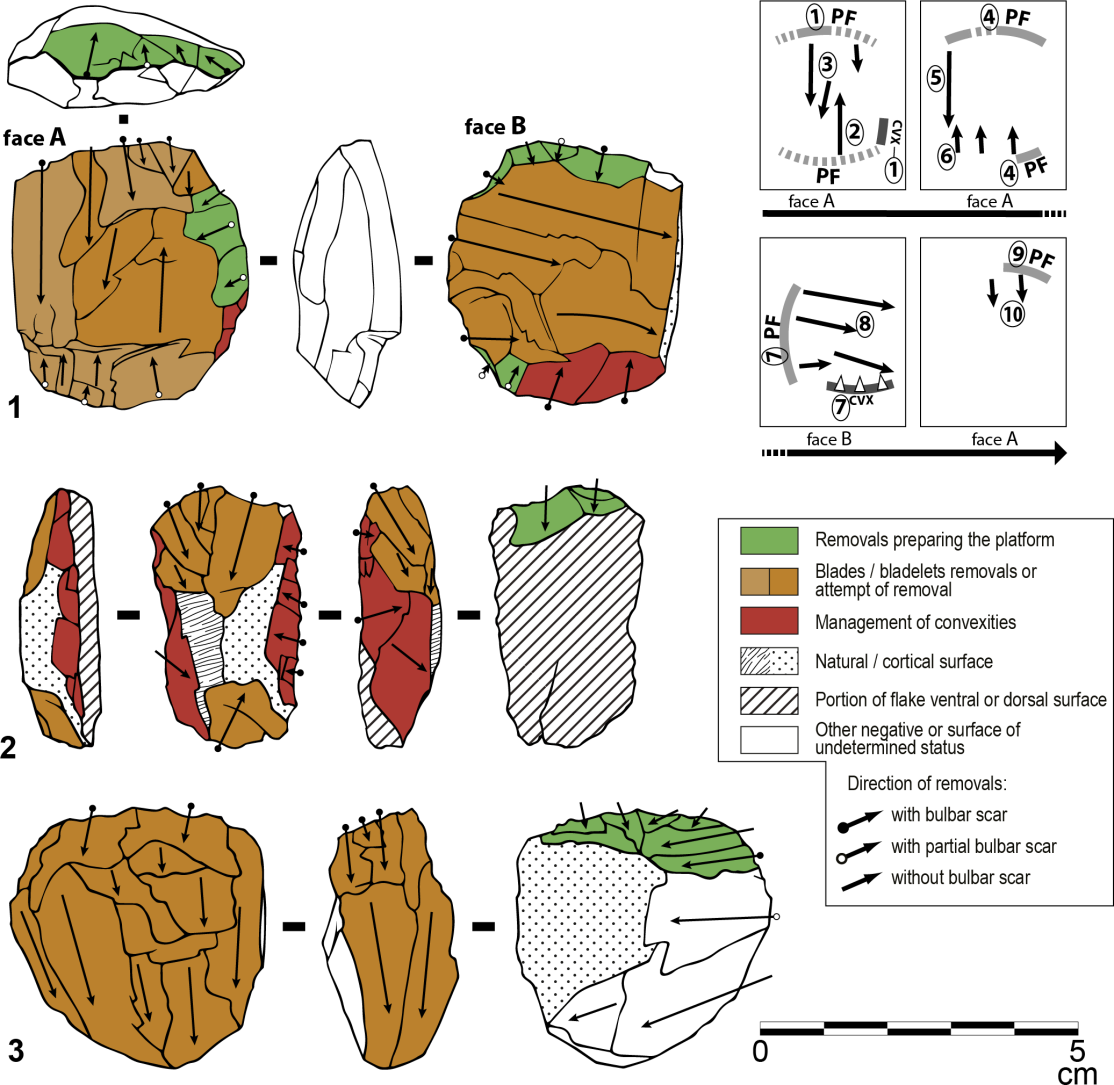
Sibudu, HP. Blade cores. (1) Core with two opposed surfaces exploited successively. The flaking chronology allows to distinguish remnants of lateral convexities management and preparation of striking platforms (layer PGS, hornfels). (2) Core on flake discarded after failure of the bladelet production. Lateral convexities were prepared by highly oblique removals coming from the ventral face of the flake (layer HI inPGS, dolerite). (3) Core discarded after a long sequence of unidirectional blade/bladelet production. The exploitation ultimately turns toward the narrow face of the volume (layer PGS, dolerite).

**Fig 17:**
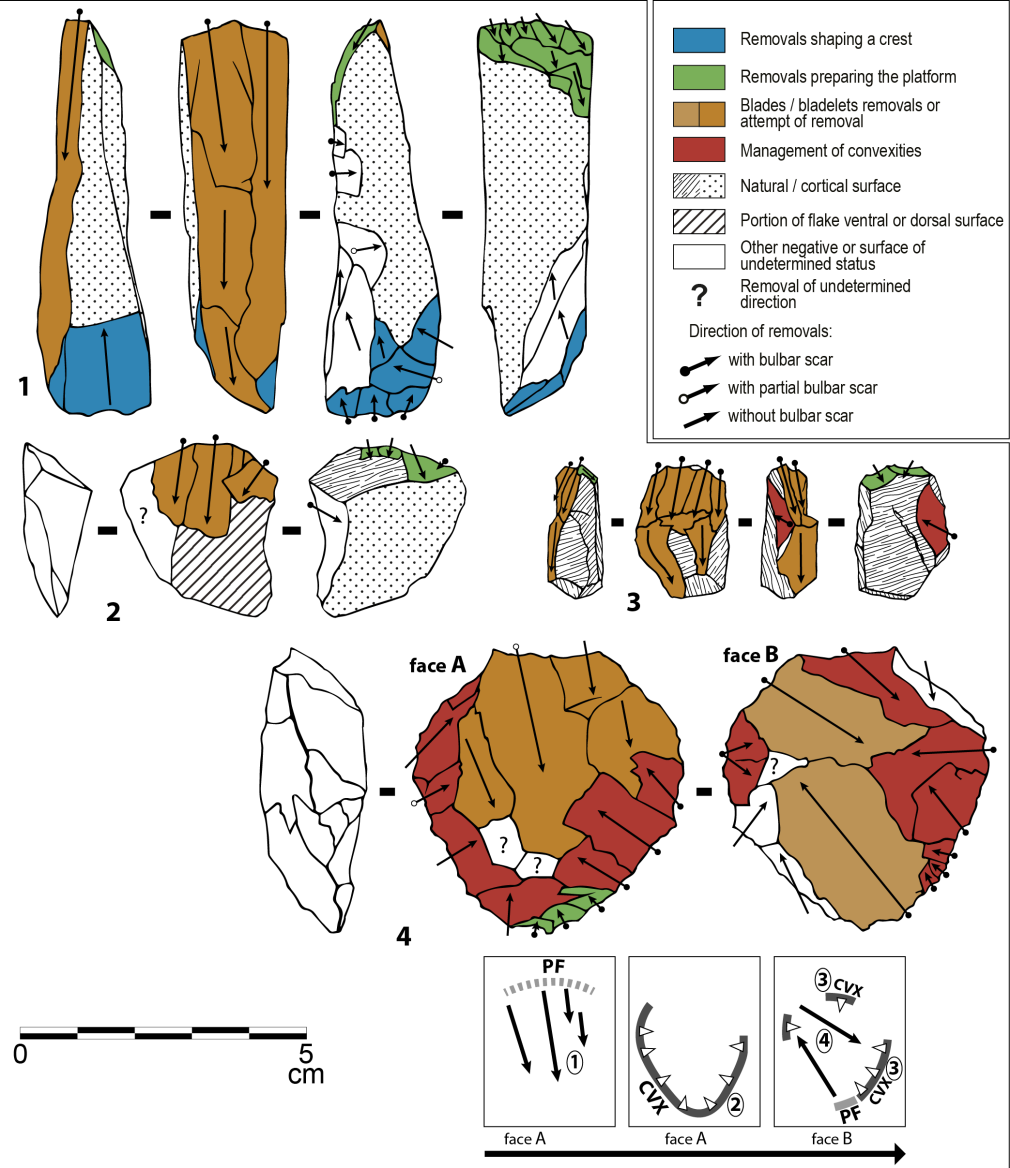
Sibudu, HP. Blade cores. (1) Prismatic core with basal remains of a frontal crest (layer PGS, hornfels). (2) Core on flake discarded after a short attempt to produce bladelets on ventral face of the core without any preparation (layer Hd inGR, sandstone). (3) Bladelet core on a crystal quartz with hinged removals. Natural convexities of the crystal were used to extract the first bladelets (Layer PGS, crystal quartz). (4) Core with two opposed surfaces exploited successively. Lateral and distal convexities were extensively managed on face A, but this was not followed by blade production. After a preparation on face B, only flakes were produced (layer HI inPGS, dolerite).

As at Klasies and Rose Cottage Cave, most of the cores have two opposed surfaces and the debitage developed preferentially on the largest face of the volume (Fig 16: 1–3)(Fig 17: 2–4) and turns sometimes toward the narrowest face (Fig 16: 3). Prismatic cores are less frequent (Fig 17:1). The coexistence of heavily reduced cores (Fig 16: 1) and cores prepared on flakes and exploited through a shortened *chaîne opératoire* is obvious (Fig 16: 2; Fig 17: 2). At Sibudu as at Klasies and Rose Cottage, blades with bidirectional scars are rare (<4%; Fig B: 12 in S4 File). A single platform was used whereas alternation from one to the opposed platform was uncommon or occurred only at the final stage of the exploitation. The frequency of crested blades is weak (2–3% of blades; Fig C: 2 in S4 File), as that of blades underlying a crested blade (Fig B: 11 in S4 File and Fig C: 5, 16 in S4 File). A similar or lower proportion was observed at Rose Cottage Cave [20] and Klasies [21]. Exceptionally, distal remains of a frontal crest extending up to the base of the core could be noticed (Fig 17: 1). At Klasies, production of blades on the largest face of the core volume is sometimes associated with the preparation and the maintenance of lateral and distal convexities by means of strongly secant removals orthogonal to the blade direction [21]. This process was also in use at Sibudu HP, resulting in few, but typical blades with orthogonal negatives on one side (Fig B: 4 in S4 File; Fig C: 4, 14 and 17 in S4 File). It was observed on cores discarded after short sequence of production (Fig 16: 2) and also on reduced cores where an almost complete refreshing of convexities was performed (Fig 17: 4). Sometime a single edge of the debitage surface was prepared that way (Fig 16: 1 A and B). However, in many cases the debitage surface was not prepared and the initial blade extraction followed natural convexities. We conclude that blade production in the HP from Sibudu was very close to the one we described at Klasies. The same package of technical knowledge was shared by these craftsmen.

#### The Howiesons Poort tools

Formal tools are dominated by backed pieces. At Sibudu their frequency varies from 71.8 in PGS to 65.2 in GR (Table 11). These high values are what can be expected in a “classic” HP, such as layers EMD and MAS of Rose Cottage where backed pieces represent 68.4% and 60.0% of the retouched pieces, with a strong decline in the upper HP levels (ETH and SUZ). Layer 20 and 10 at Klasies River (excavations by Singer and Wymer) also have very high frequencies of backed pieces: 67.6% and 83.1%. Keep in mind that the Klasies values are higher because tool fragments and utilized flakes were not counted nor catalogued in the Singer and Wymer’s 1982 monograph [80].

In contrast Deacon’s sample of the HP Lower has only 38.4% of backed pieces. It could be argued that his excavation area was limited to only 1 square meter (Table 10). However at Klipdrift Shelter the frequency of backed pieces (segments, other backed tools and oblique truncations) is also quite low, 39.8% (70/176) for a larger excavation area. At Diepkloof the frequencies of backed pieces are also low, reaching 48.2% (98/203) only in the Late HP. At Border Cave, where the HP is dated between 74 ± 4 and 56 ±2 ka [86], the frequency is 33.0% (61/124). Other kinds of differences between sites can be observed: differences in backed piece morphology, e.g. partly backed pieces are predominant at Rose Cottage but much less common at Sibudu and Klasies; *pièces esquillées* are well represented at Sibudu and Diepkloof, but much less so at Klasies and Rose Cottage. This intersite variability in proportions of tool classes is probably due to contingent factors such as the nature of occupation and temporal and geographic separation.

**Table 10:** Size of excavation areas for the HP samples discussed in this paper.

HP excavation area	Square meters
Sibudu	4
Rose Cottage (a)	6
Klasies River main site Cave 1A Singer and Wymer’s excavations	6
Klasies River main site Cave 1A, Deacon’s excavations	1
Diepkloof	2
Klipdrift Shelter	6.75

**Table 11:** Frequencies of formal tools in the HP levels of Sibudu, Klasies River Cave 1A and Rose Cottage.

	Sibudu					Klasies Cave 1A		Rose Cottage	
	GR		GS		PGS	Deacon's excavation HP Lower		All HP levels	
Types of tools^1^	N	%	N	%	N	N	%	N	%
Backed pieces	75	65.2	86	72.9	120	48	38.4	177	56.2
Burins	1	0.9	0	0.0	0	0	0	6	1.9
End scrapers and circular scrapers	0	0.0	0	0.0	2	4	3.2	1	0.3
Side and convergent scrapers	1	0.9	0	0.0	9	3	2.4	17	5.4
Unifacial points	0	0.0	0	0.0	1	0	0	1	0.3
Awls	0	0.0	1	0.8	1	0	0	3	1.0
Denticulates and notches	2	1.7	3	2.5	3	17	13.6	13	4.1
Scaled pieces	12	10.4	8	6.8	5	0	0	4	1.3
Retouched and utilized blades and flakes	13	11.3	13	11.0	18	40	32	89	28.3
Tool fragments and indet.	11	9.6	7	5.9	8	13	10.4	4	1.3
Total	**115**		**118**		**167**	**125**		**315**	

^1^Bifacial points are excluded from these counts to avoid confusion with the 17 Still Bay points which are intrusive in layer PGS and have been assigned to the Still Bay. Quartz bifacial points have been described by [37]. Relative frequencies of retouched pieces in PGS are also not indicated because, as explained, mixing by intrusive elements affects all four squares of our PGS sample. Backed tools are very distinctive and we can confidently assign them to the HP. But we cannot assign all other retouched pieces of PGS to either the Still Bay or the HP. Deacon’s sample of Klasies HP Upper and DRG from Sibudu are not included because the samples are quite small.

At Sibudu there are no strong differences between layers for classes of backed pieces (Table 12). No clear trend appears even when using sublevels (Fig E in S4 File). There is only a decline in the number of segments in GR compared to GS. As at Rose Cottage [20] triangles and trapezes are either absent or present in small quantities while they are slightly more common in the early phases of the HP at Klasies [21]. Segments are predominant, as at Klasies (Figs F and G in S4 File).

**Table 12:** Frequencies of classes of backed tools in GR, GS and PGS.

	PGS		GS		GR	
	N	%	N	%	N	%
Segments	43	35.8	44	51.2	25	33.3
Triangles	2	1.7	0	0.0	2	2.7
Trapezes	2	1.7	2	2.3	0	0.0
Completely backed	10	8.3	5	5.8	7	9.3
Partly backed	9	7.5	9	10.5	8	10.7
Irregular forms	3	2.5	4	4.7	3	4.0
Truncations	2	1.7	4	4.7	4	5.3
Fragments	49	40.8	18	20.9	26	34.7
**Totals**	**120**		**86**		**75**	

Backed tools are made on blades in 95 to 99% of the cases at Sibudu, Rose Cottage and Klasies Cave 1A (Table B in S5 File). Blades from the optimal phase of debitage, i.e. blades without cortex and from the central part of the debitage surface, were selected for making backed pieces in most cases (Table C in S5 File). Other kinds of tools were also made on flakes, but blades were still preferred in high frequencies (Table D in S5 File). This high degree of uniformity in the selection of blanks for tool production is even more impressive if one considers the diversity of raw materials in blank shape and knapping quality at the three sites.

The platform is removed in most cases (93, 87 and 94% of the cases in PGS, GS and GR respectively) and similar frequencies have been observed at Klasies [21]. This kind of reduction in size was probably done to facilitate hafting since the proximal part of a non-cortical blade is also its thicker part. Removal of platforms at Rose Cottage was much less common, probably to avoid reduction in size of the rather small blanks [20]. Occasionally the platform is removed by a snap fracture, left as is, perhaps intentionally to avoid penetration in the socket on impact (Fig F: 6, 9 in S4 File). The possible use of pressure retouch for making a back on blades is discussed in S6 File.

#### Raw material and size of backed pieces

**Fig 18:**
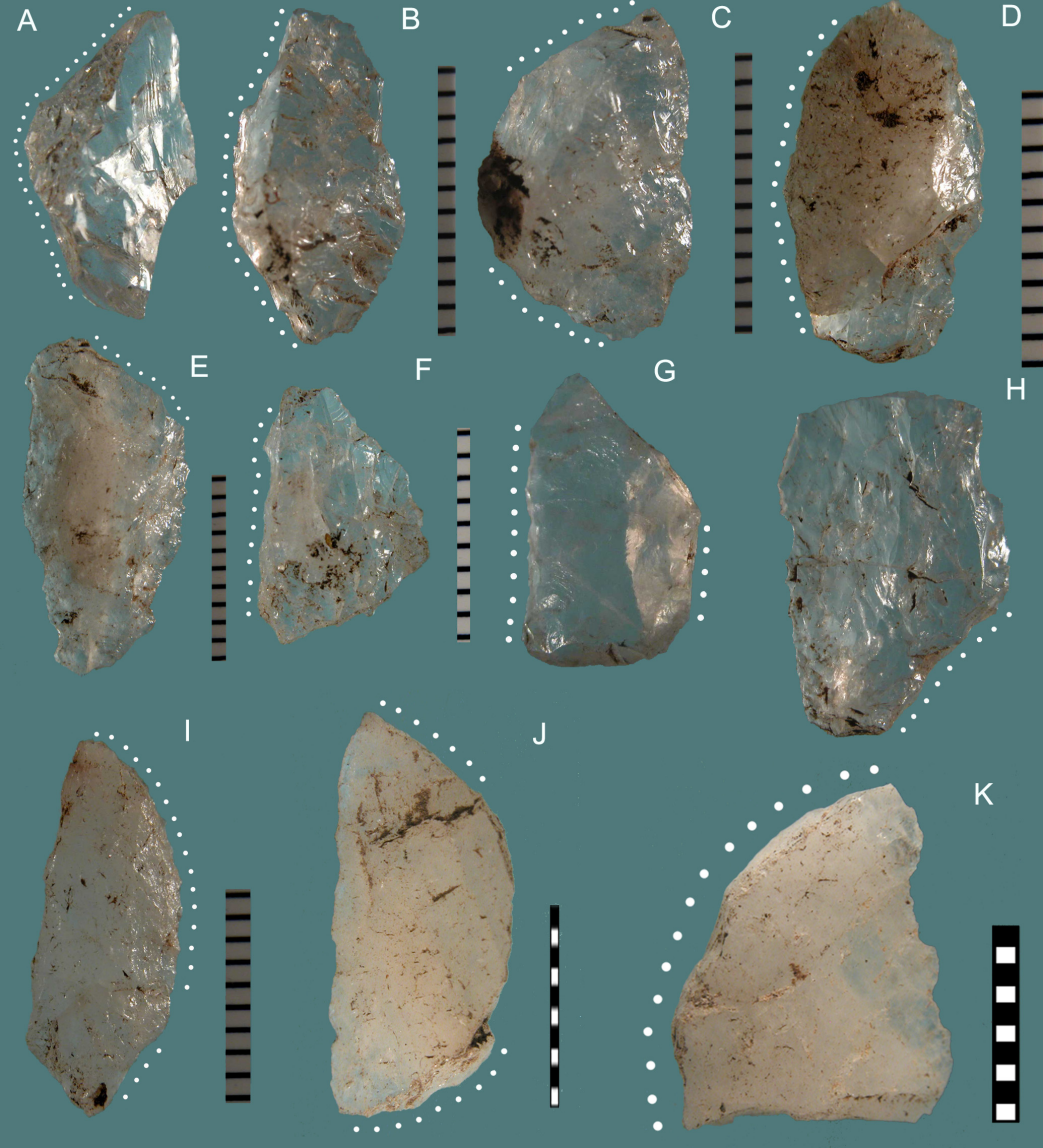
Sibudu HP: Quartz backed pieces from GS, except I and K from GSII. The white dots indicate backing abrupt retouch, all crystal quartz except I-K of milky quartz. (A, B) segments on indeterminate blank; (C) partly backed piece on a flake; (D) completely backed piece, indeterminate blank; (E) oblique truncation oon a bladelet; (F) straight back piece, irregular shape on a flake fragment; (G) double-backed piece on a bladelet; (H) oblique truncation on a bladelet; (I) segment on a bladelet; (J) partly backed piece, indeterminate blank; (K) broken segment, indeterminate blank. Scale bars in mm.

At Sibudu three kinds of raw material were used for backed pieces: hornfels, dolerite (including a fine-grained variety) and quartz (both crystal and milky quartz, [87]). Quartz (Table E in S5 File) is relatively frequent in layers GS and GS II, but it declines to only 3% in GR at the very top of the sequence (Fig I in S4 File). As at Klasies River Cave 1A there is no clear correlation between raw material and size of pieces. The length distributions of Figs J and K in S4 File do not show distinct modalities, except for quartz pieces the size of which is controlled by the size of the available blanks, small pebbles and rounded nodules generally 4–5 cm in size. This is also indicated by the small size of quartz non-backed pieces such as scaled pieces and other retouched pieces which are all smaller than 4 cm, with an average of 2.0 ± 9.3 mm (of 10 measurable pieces). In other words, their dimensions are strictly dependent on the size of the available raw material and not the result of intentional reduction in size. The diversity of forms of quartz backed pieces (Fig 18), similar to that in hornfels and dolerite, except for the double-backed piece (Fig 18: G) which has no counterpart in other raw materials. (Fig L in S4 File) illustrates the variability in size of hornfels backed pieces, as also noted by Wadley and Mohapi [88].

#### Chemical analysis of hafting adhesives

Black residues of resin or plant gum, sometime mixed with ochre, have been documented at Sibudu by optical microscopy in combination with the spatial distribution of residue on the base of stone points or on the back of backed pieces clearly suggesting that the adhesive was used for hafting [89,90]. Replicative experiments show that plant gum mixed with ochre forms adhesives that are robust and dry fast [91]. However optical microscopy alone cannot securely distinguish between plant gums (polysaccharides, e.g. from *Acacia*) and resins (typically terpenoid secretions from plants, particularly coniferous trees) [92].

One of us (I.D.) applied gas chromatography/mass spectrometry (GC/MS) to three segments from Sibudu GR C6c, GSII B6a and PGS B5a and one segment from Rose Cottage. The three segments from Sibudu had ochre lines and possible residue on the backed edge; the segment from Rose Cottage was described with residue [93]. Microsamples (less than 1 mg) were collected from the specimens and submitted to a combined analytical procedure for the identification of lipids, waxes, proteins, resinous materials and saccharides [23,94,95]. No significant molecular markers of organic materials were detected. The three backed segments form Sibudu were then dipped in extraction solvent for the first step of the combined GC/MS analysis. Two (PGS B5a and GR C6c) gave significant results (Figs M and N in S4 File). They contained a relevant amount of diterpenes, indicating the use of a conifer resin, and some lipid material. No polysaccharides (plant gum) were detected. The main components of the diterpenic fraction are acids indicating a resin from a plant belonging to the conifer family. In particular *Pinaceae*, *Podocarpaceae* and *Araucariaceae* may produce a resin containing abietadenic and pimaradienic components. Among South African plants *Afrocarpus* (syn. *Podocarpus*) *falcatus* contains relevant amount of these compounds and is a probable candidate as a source of the resin used as hafting material. Analysis of charcoal from the GS, GR and GR2 layers of Sibudu shows that *Podocarpus* spp was predominant while *Acacia* is absent [96]. The use of conifer resin for hafting (*Podocarpus elongatus*) is also indicated on a quartz backed flake dated to ca. 56 ± 10 ka in the Late HP at Diepkloof [97]. The hypothesized use of plant gum as a hafting adhesive at Sibudu requires further testing.

#### Hafting modes and impact scars

Based on microscopic analysis of residues, their spatial distributions on tools and experimental replication it has been suggested that the backed pieces, especially those of hornfels and dolerite may have been hafted diagonally as well as longitudinally [88–90,98]. Two kinds of evidence support these interpretations. Black stains or ochre stains (ochre was used as an additive to the adhesive; [89–91]) occur on the back of segments and other backed pieces. The “hafting” lines can be oblique or parallel to the working edge marking the oblique or longitudinal position of the haft (Fig O in S4 File). These hafting lines were never observed on the Klasies backed pieces; their absence may be due to lack of preservation. At Sibudu mixing resin with ochre made these lines more visible, but resin was not mixed necessarily with ochre, as was the case at Diepkloof [97].

The second line of evidence is provided by impact fractures. Our identifications of impact scars are based on direct observation and analysis of impact fractures on Gravettian backed microliths, and experimental replicates shot with a bow [99]. We have also used data from Geneste and Plisson experimental series [64] and archaeological data from 160 impact fractures on Paleoindian points from bison kill sites (cf. section on the Blombos Still Bay). In all cases scars are on artifacts of known function.

**Fig 19:**
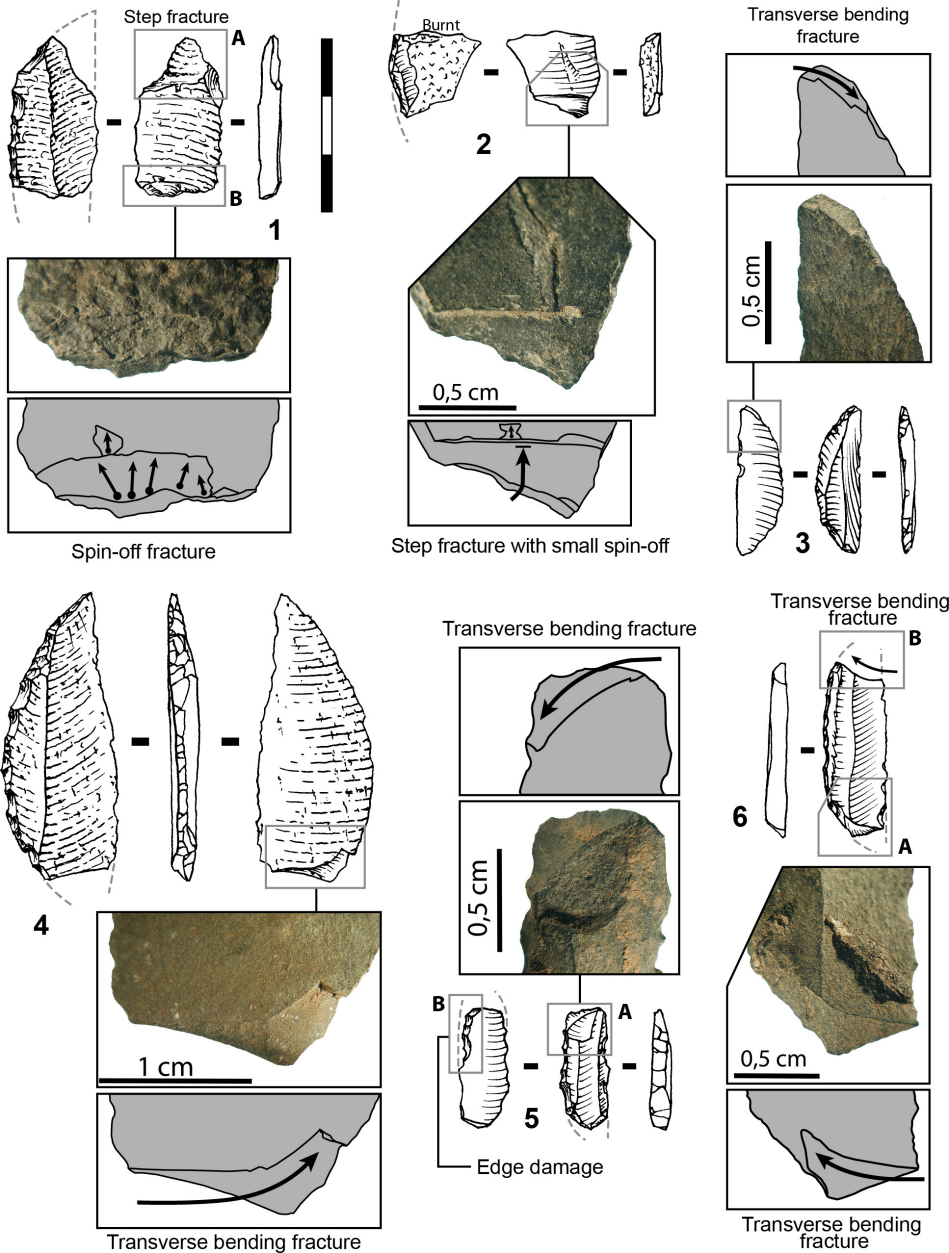
Sibudu HP: Examples of impact scars on backed pieces. (1) GRII B5a P7, burnt dolerite with a distal step fracture and spin-off fracture at the base. (2) PGS B6a 155, burnt hornfels fragment with a step fracture and small spin-off. (3) GS B5c M14, hornfels segment with a transverse fracture 3.6 mm long. (4) PGS C6b 73, fine dolerite segment with transverse fracture at the base. (5) GRII B5b B2, hornfels with a proximal transverse fracture and edge damage. (6) GS under rock B5b 27 hornfels with transverse fractures at tip and base. In all cases the fractures are superimposed on the backing.

**Fig 20:**
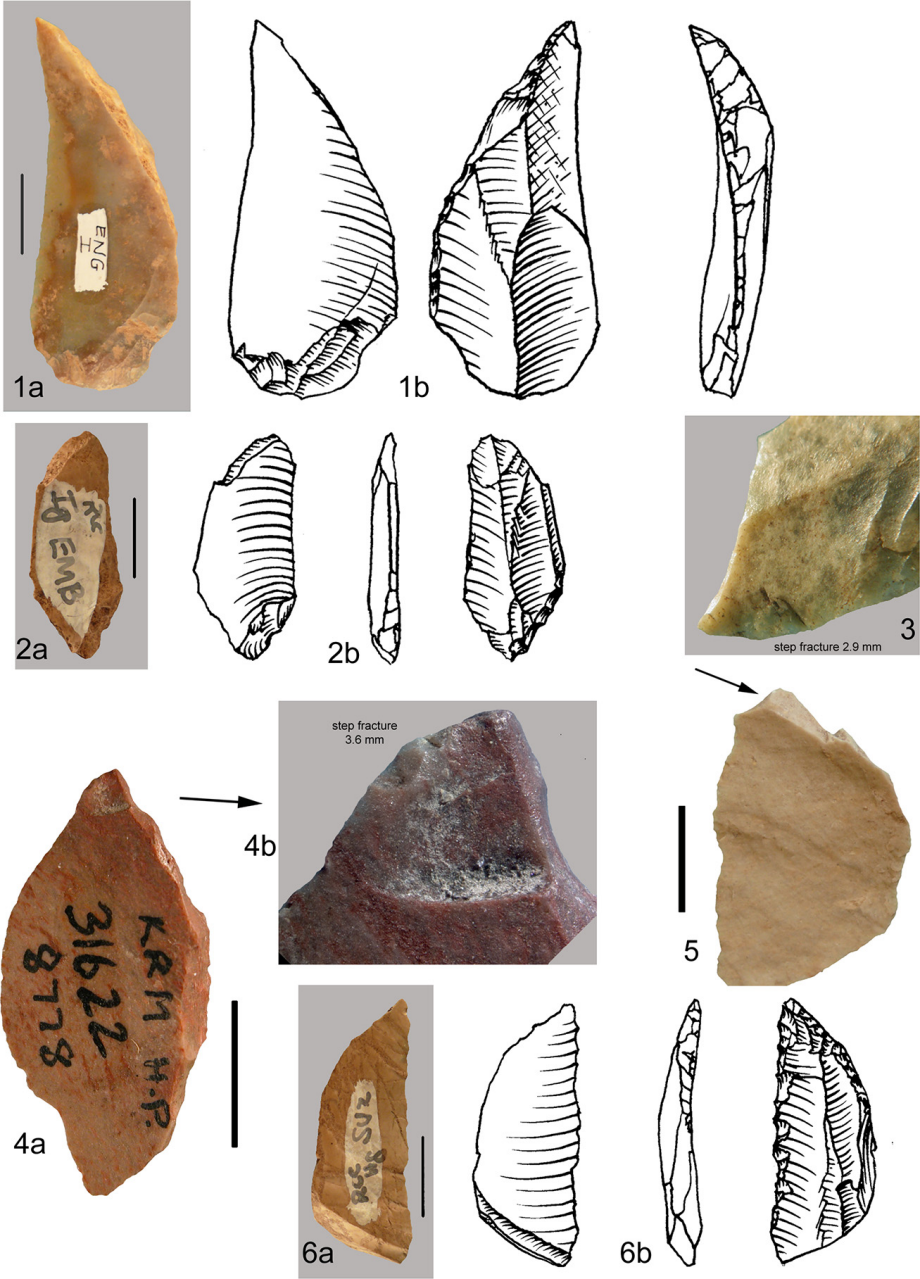
Oblique and axial impact scars on Rose Cottage and Klasies River Cave 1A backed pieces. (1a, b) Rose Cottage ENG no. 9, opaline; multiple bending fractures at the base 10.9 mm long; (2a, b). Rose Cottage EMB no.4, opaline; partly backed piece with oblique impact scar, 9 mm long; (3) Rose Cottage EMA no.2, opaline; axial step scar 2.9 mm long. (4a, b) Klasies River Cave 1A, Singer and Wymer layer 20 no. 165 silcrete, axial step fracture 3.6 mm long on ventral face; (5) Klasies River Cave 1A layer 20 no. 151 silcrete; oblique impact scar 6.0 mm long; (6a, b) Rose Cottage SUZ opaline, this is typologically a perforator; oblique impact scar 13 mm long. Scale bar = 1 cm.

With one exception (see spin-off below) the impact scars occurring on backed pieces are bending fractures, that is, they do not have a negative bulb of percussion generally resulting from force applied over a relatively small area. The smooth initiation indicates forces distributed on a larger area [100]. Diagnostic impact scars are: step-terminating fractures oriented axially (Fig 19: 1, 2) and transverse fractures removing a portion of the tip or base, oriented either perpendicularly or obliquely, in sharp angle (Fig 19: 3, 5A, 6A) or blunt angle (Fig 19: 6B) relative to the axis of the backed piece. These burin-like fractures result from a bending stress as they lack negatives of bulbar scar. Spin-off is a small secondary fracture initiating from the surface of a bending fracture and may have a negative bulb of percussion, like a cone fracture [100]. It is not common on the HP backed pieces, but it does occur (Fig 19: 1b and 2)(Fig H: 4b in S4 File). These kinds of oblique and axial impact scars occur at Rose Cottage and Klasies (Fig 20)(Table 13).

**Table 13:** Impact scars ≥ 2 mm at Sibudu and other HP sites^1^.

	Pieces with impact scars	Backed pieces	Impact scars
Layers	N	N	%
Sibudu, layer GR	4 (3)	41	9.0
Sibudu layer GS	7 (5)	46	15.2
Sibudu layer PGS	7 (3)	78	9.0
Klasies Cave 1A, layer 20	9 (7)	128	7.0
Klasies Cave 1A, layer 10	5 (3)	94	5.3
Rose Cottage, all HP levels	8 (4)	117	6.8

^1^The total number of backed pieces does not include undetermined (mainly broken) cases and straight truncations. Transverse bending fractures (in parenthesis) are included in the total number of scars.

What can we deduce from this pattern about hafting position of HP backed pieces? Position and orientation of step fractures and spin-off are unequivocally associated with axial hafting and suggest a vertical position of the stone at the tip of the shaft and can occur at tip or at the base of the backed piece.

In the experiment on Gravettian microliths (narrow backed points) one of us [99] observed that the orientation of impact fractures was strongly determined by the orientation of the longitudinal axis of the hafted microliths [65]. When hafted obliquely relative to the dynamic axis of the projectile, oblique fractures occur more frequently than perpendicular fractures. Almost the same conclusion was reached by Yaroshevich et al.: “it appears that fractures oriented obliquely to the longitudinal axis of the microlith are most common and occur on obliquely hafted points and barbs, as well as on transversal points” [101]. Thus both hafting lines and oblique fractures support the suggestion that backed pieces were hafted diagonally as well as longitudinally.

*Frequency of impact scars*. The frequency of impact scars observed by us (Table 13) is lower than those proposed by other researchers [89,102,103] for Klasies River Cave 2 (21%) and the Sibudu backed pieces (24%). Yet comparable low frequencies of impact scars occur on unifacial points of post-HP times and on the Blombos Still Bay bifacial points (Table 14). Since experiments provide high frequencies of impact scars on artifacts used for shooting animals (about 40%; [90,100]) it has been argued that low proportions of diagnostic impact fractures could mean that the points were not used as weapons or that were made for that purpose but were not used. While experiments are very useful for understanding the morphology of impact scars, it does not seem logical to expect them to provide diagnostic frequencies for archaeological samples. In most experiments points were shot into animals several times or until damage rendered the point useless. Comparison of frequencies of impact scars at archaeological sites where the point function is well-known (e.g. Late Paleolithic to Bronze Age sites in Europe and the Paleoindian Casper site) show that frequencies are high only at kill sites (such as Casper and Stellmoor) while at residential or manufacturing sites (such as Sibudu, Blombos and Rose Cottage) frequencies are lower and quite variable (Table I in S5 File).

**Table 14:** Impact scars on MSA unifacial and bifacial points.

Site	Impact scars (n)	Impact scars (%)
Sibudu layer RSP (n = 101)	9	8.9
Rose Cottage, all post-HP levels (n = 48)	4	8.3
Blombos Still Bay (n = 82 phase 3 points)	11	13.4

#### Changes in knapping techniques through time

The upper part of the HP sequence at Klasies and at Rose Cottage is marked by changes in knapping techniques. The regularity of blades decreases and there are changes in the manner of percussion from marginal to more external with an associated increase in the thickness of platforms, less elaborate platform preparation and wider blades. There is a decline in the frequencies of blades, especially marked at Rose Cottage and an increase in the production of flakes which are more often used as tool blanks [20,21].

Is this pattern of changes the same at Sibudu? As at Klasies, there is an increase in blade width at the top of the Sibudu HP sequence, the mean width of GR is statistically different from the mean width of GR2 (Table G in S5 File). The frequency distribution shows that the curve for GR is offset toward wider values (Fig D in S4 File).

A detailed study of blade platforms from layers GS and GR allows us to conclude that there are only minor differences in the knapping techniques between these two layers. Blade platforms in GS and GR are similar in thickness, but differ slightly in width (Table H in S5 File). Preparation of platforms show minimal changes between layers GS and GR (Table A in S5 File).

It seems that the HP knappers of layer GR used their soft stone hammer with a motion slightly different than in GS, inducing a more internal point of percussion. We must conclude that within the Sibudu HP changes in knapping techniques are subtle compared to the clear modification of knapping gesture described for the late HP at Rose Cottage Cave and Klasies [20,21].

#### Changes at the end of the HP

The drastic changes in tool classes between the upper HP layers at Sibudu and other sites and the post-HP layers above (Table J in S5 File) suggest a rapid disappearance of the Howiesons Poort and might seem to support a saltational process of cultural evolution characterized by major discontinuities in cultural transmission driven by changes in population size [104] or by climatic changes [105]. However the changes observed at Sibudu are in strong contrast with the more gradual changes to more flake-based lithic production in the upper levels of the HP at Klasies and at Rose Cottage. Evidence from these sites clearly indicates that the HP underwent incremental transformations [20,21]. If behaviour at Sibudu followed the same trajectory, then it seems that the final phases of the HP sequence are not represented at Sibudu.

The conventional view of HP backed pieces is that they were used as tips or barbs or cutting inserts for hunting weapons (but see [93,106]). Yet the low proportions of tools other than backed pieces at residential sites such as Sibudu and at Rose Cottage, where both hunting and domestic tools are expected (Table F in S5 File), suggest that many backed pieces were used as cutting edges, thus fulfilling functions that in other periods were executed with a greater variety of tools [20,21,107]. The idea that not all backed pieces were involved in hunting weaponry is in agreement with patterns of Sibudu HP faunas. Based on high taxonomic diversity, high frequencies of small mammals and forest-living species Lyn Wadley [108] proposed that snares and traps were used during the HP. Post-HP assemblages where unifacial points were the hunting weapon of choice have much higher proportions and a greater variety of “domestic” tools [20–22].

#### Innovation and standardization

The real innovation of the HP craftsmen at least in the phases represented at Rose Cottage, Klasies River and Sibudu, consisted of the routine production of thin blades from the wide surface of the core using marginal percussion and of the widespread use of blades, large and small, for hunting weapons and domestic tools [20,21]. However, the forms and size of backed pieces, their symmetry and the extent of edge working vary within the same HP assemblage and between older and younger HP assemblages; each piece is not a copy of another. In other words the products were not standardized, although the practice of making them was.

## Conclusions

One particular hypothesis we use in comparing assemblages through space needs a prior explanation. We have assumed that distance in geographic space can affect how similar lithic technologies are between sites; in other words, that distance is a proxy indicator for likelihood of interaction between assemblage makers. That distance affects similarities may seem obvious, or at least it is so to art historians. To give just one example Celtic coinage of the 3rd to 1st century BC imitates Greek coinage used in the Greek colony of Marseille (Southern France). Coins made in Northern France and other places distant from Marseille developed their own particular style and iconography [109].

For prehistorians it becomes a more uncertain inference since we lack data on trade, cultural exchanges and social relations between artifact makers. Thus similarities across large geographic distances would suggest close social interactions, if similarities are strong and numerous enough to exclude convergence. It is still true however that the arguments presented below are only plausible hypotheses.

Similarities in technology and morphology of backed pieces, uniformity in the selection of blanks and in proportions and kinds of impact scars, between sites spread across a vast region (Rose Cottage and Klasies are separated by more than 600 km and there are 1200 km between Klipdrift Shelter and Sibudu, in a straight line) and using different raw materials (opaline at Rose Cottage, silcrete and quartzite at Klasies, hornfels and dolerite at Sibudu) in addition to the early phases documented at Diepkloof strongly suggests that the Howiesons Poort was a relatively long-lasting system of complex behavioral traditions that may have been socially transmitted by teaching and verbal instructions. The engravings on the ostrich eggshell containers documented in the Howiesons Poort of Diepkloof [110] and Klipdrift [2] indicate the existence of a graphic tradition of communication among members of the MSA groups inhabiting the site. However connecting archaeological objects to inferences regarding cognitive abilities and language is a very controversial issue, thus our statement remains a speculation.

The preference for the production of elongated blanks, the way they were made, their selection for retouched tools and the way they were retouched are the basic elements that define the Howiesons Poort of Sibudu, Rose Cottage and Klasies River as a cultural unit with a wide geographic range, temporal duration and developmental changes through time [19]. According to [14] an "industry" is a group of related assemblages which share a large number of technological and typological features in recurrent association. This is the basic formally named cultural entity which can be defined only after analysis of a large body of materials. The Howiesons Poort is such an entity.

At present the Still Bay assemblages suggest temporal and spatial discontinuity. Technological description of well-excavated assemblages with stratigraphic integrity other than Sibudu and Blombos are preliminary (Diepkloof; [3]) or limited to Still Bay points (Hollow Rock Shelter; [73]); many sites are surface occurrences [1] or lack clear stratigraphy (Umhlatuzana; [111]) and there is clear variability in the way bifacial tools were shaped and used. What we know of these assemblages falls short of what is considered necessary by [14] to cluster them as a homogeneous set, although elements of similarity are certainly present. This seems to be a problem of sampling. Future research might provide the technological, spatial and temporal observations needed to see if the Still Bay shows continuity in technological patterns and directional change comparable to those of the classic Howiesons Poort.

## Supporting Information

S1 File Figs A-X. (PDF)

S2 File Tables A-N. (PDF)

S3 File Materials and Methods. (PDF)

S4 File Figs A-O. (PDF)

S5 File Tables A-J. (PDF)

S6 File Pressure flaking in the Howiesons Poort. (PDF)
