# Supplementary material for: The Still Bay and Howiesons Poort at Sibudu and Blombos: Understanding Middle Stone Age Technologies
Source: PLoS One. 2015 Jul 10;10(7):e0131127. doi: 10.1371/journal.pone.0131127 (PMC4498762; doi:10.1371/journal.pone.0131127)
Supplement: S1 File — (PDF) [file pone.0131127.s001.pdf]

The Still Bay and Howiesons Poort at Sibudu and Blombos: Understanding Middle Stone Age technologies

Sylvain Soriano, Paola Villa, Anne Delagnes, Ilaria Degano, Luca Pollarolo, Jeannette J. Lucejko, Christopher Henshilwood, Lyn Wadley

Supporting Information

S1 File

(Figures A – X)

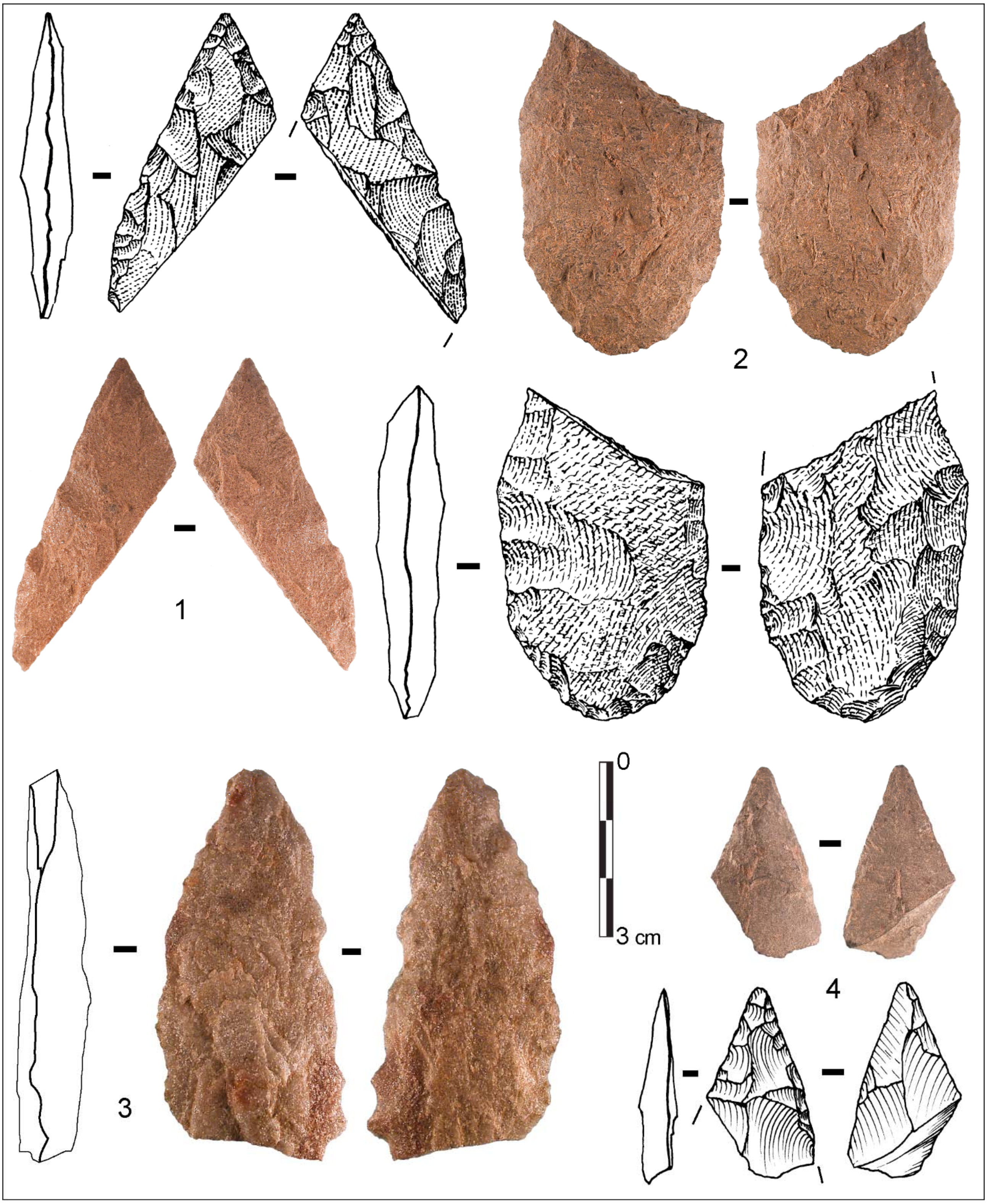

Figure A. Bifacial piece and broken bifacial points from Sibudu Still Bay, layers RGS/RGS2. Layer, square, raw material, cat. no. (1) RGS, B6a, quartzite, 18; (2) RGS, B5c, dolerite, 18; (3) RGS2, B5c, quartzite, 13; (4) RGS, B5b, hornfels, 4.

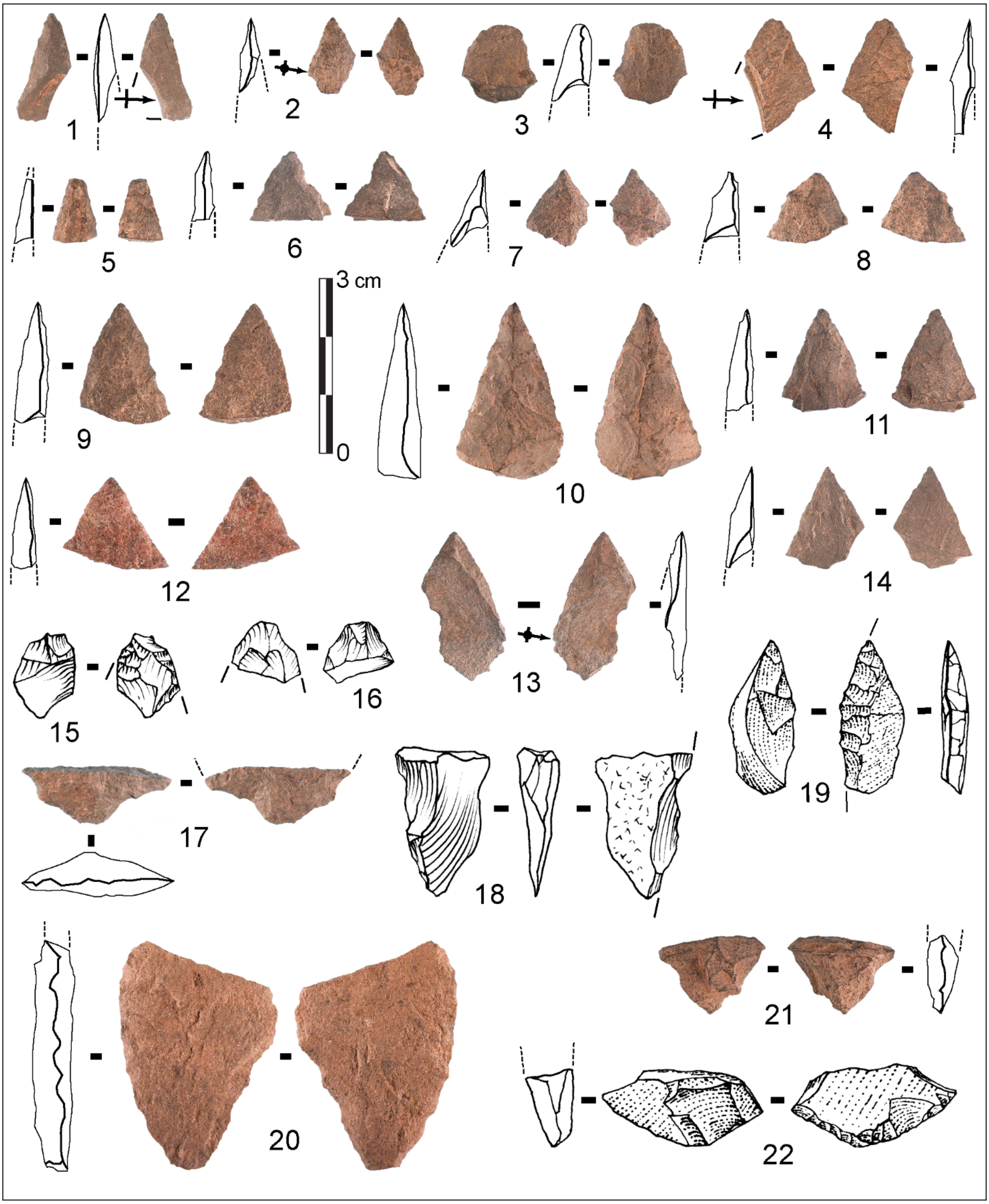

Figure B. Fragments of bifacial pieces from Sibudu Still Bay, layers RGS/RGS2. Layer, square, raw material, cat. no. (1) RGS2, B6b, hornfels, 26; (2) RGS, B6a, dolerite, 27; (3) RGS2, B5d, dolerite, 31; (4) RGS, B5d, hornfels, PV40; (5) RGS, B5c, hornfels, 25; (6) RGS, C5a, hornfels, 5; (7) RGS2, B6a, dolerite, PV56; (8) RGS, B6a, dolerite, 30; (9) RGS2, B6a, quartzite, 9; (10) RGS, B5c, hornfels, 15; (11) RGS2, B6a, dolerite, 8; (12) RGS, B5a, quartzite, 24; (13) RGS, B6a, dolerite, 19; (14) RGS2, B5b, hornfels, 6; (15) RGS2, B5c, hornfels, PV58; (16) RGS2, B6a, hornfels, PV9; (17) RGS, B5c, dolerite, PV36; (18) RGS, B5b, hornfels, burnt, PV57; (19) RGS2, B5a, quartzite, PV8; (20) RGS, B6a, quartzite, 22 (21) RGS2, B5b, hornfels, 33; (22) RGS, B5c, quartzite, PV13.

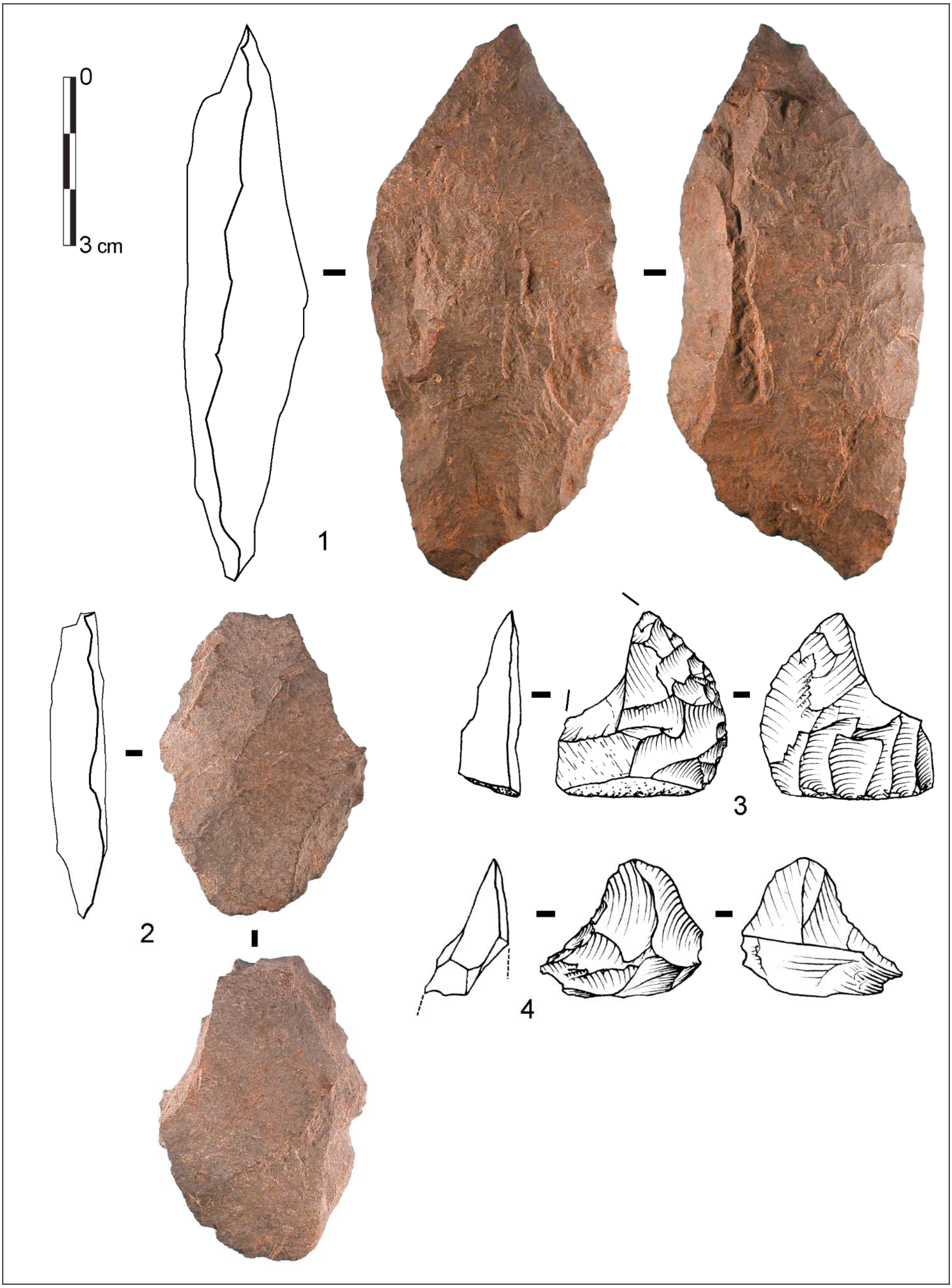

Figure C. Bifacial pieces from Sibudu Still Bay, layers RGS/RGS2. Layer, square, raw material, cat. no. (1) RGS2, B5d, dolerite, PV3; (2) RGS, B5b, hornfels, 3; (3) RGS, B5d, hornfels, PV19; (4) RGS, B5d, hornfels, PV20.

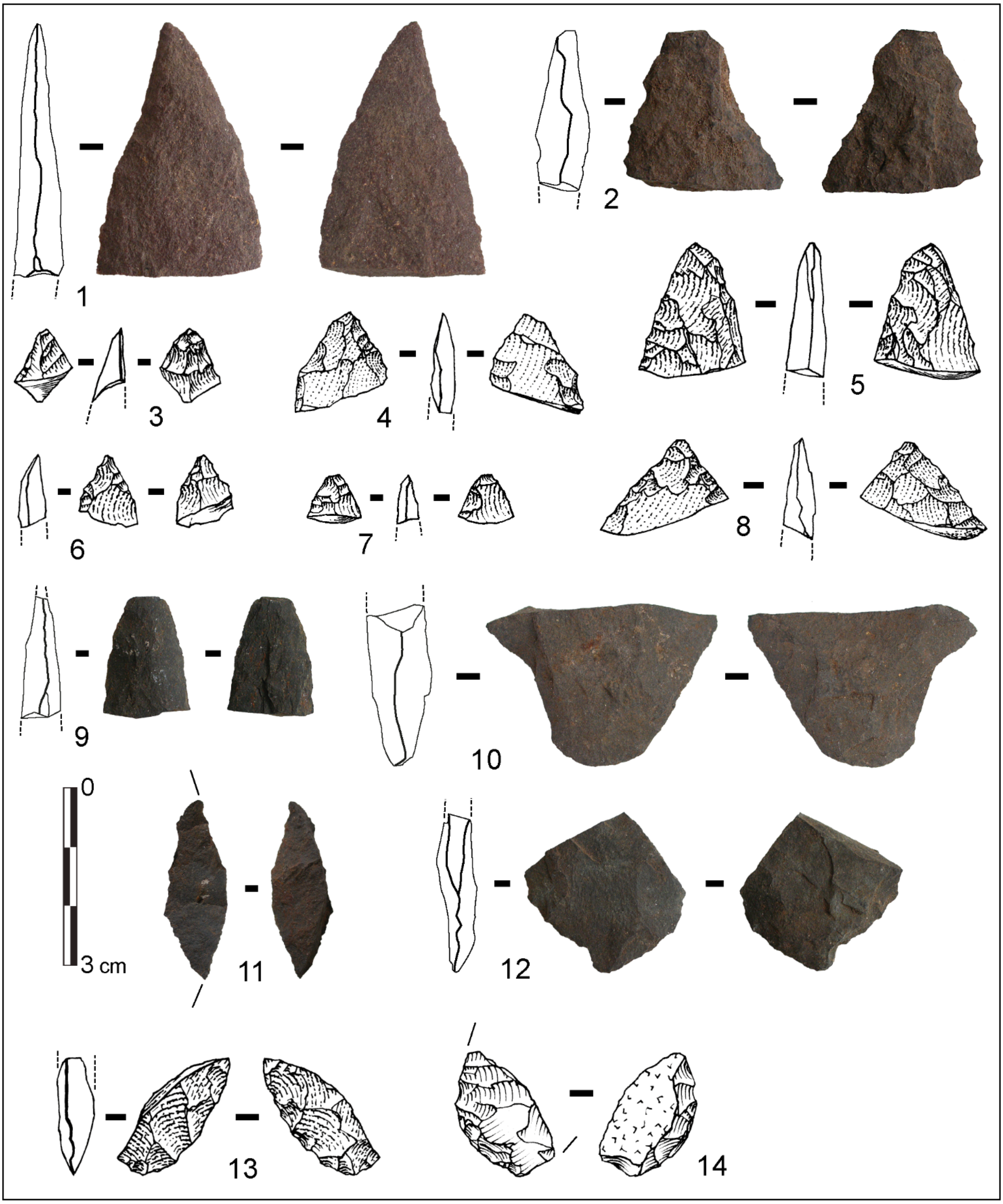

Figure D. Fragments of bifacial points from Sibudu Still Bay, layer PGS. Layer, square, raw material, cat. no. (1) PGS, C6b, quartzite, P2; (2) PGS, C5d, dolerite, P3; (3) PGS, C5a, dolerite, P9; (4) PGS, B5d, quartzite, P12; (5) PGS, B6a, dolerite, P15; (6) PGS, B5d, dolerite, P14; (7) Hb in PGS, B6b, dolerite, P17; (8) PGS, B6a, quartzite, P13; (9) PGS, C6a, hornfels, burnt, P7; (10) PGS, C6c, dolerite, P4; (11) PGS, C6a, dolerite, P8; (12) PGS, B5c, hornfels, P6; (13) PGS, B5a, dolerite, P11; (14) PGS, B5a, hornfels, burnt, P16.

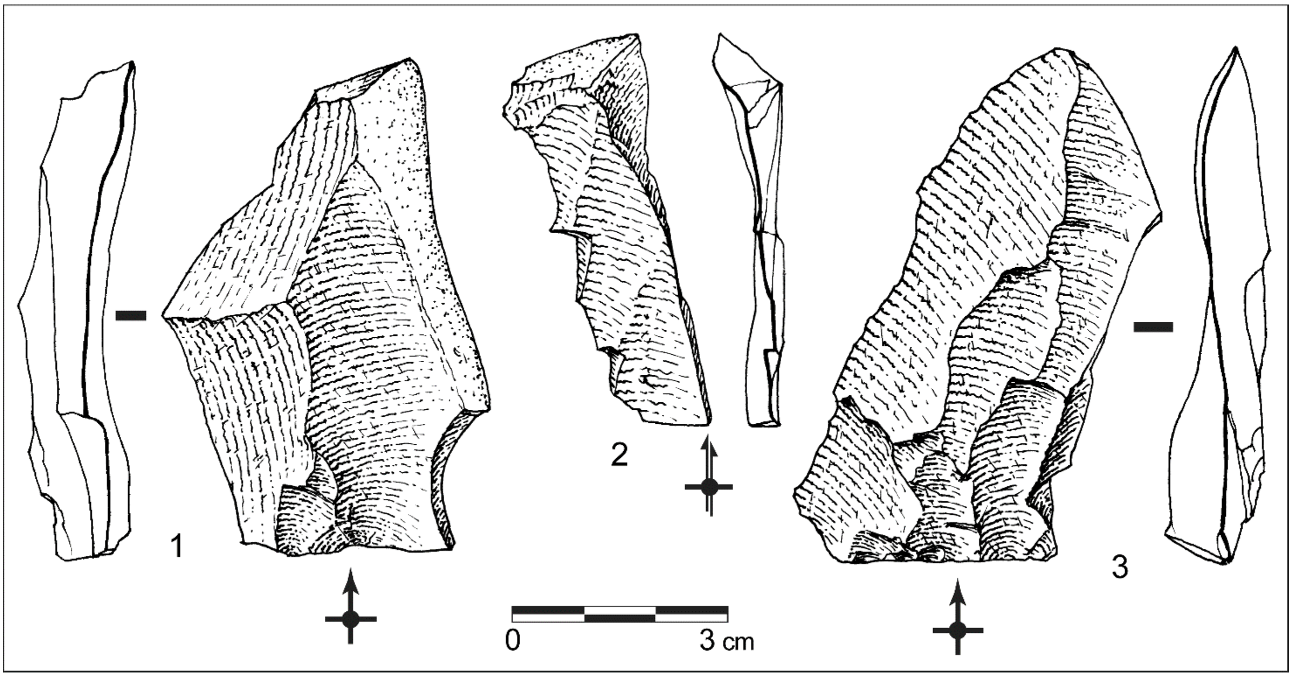

Figure E. Sibudu, Still Bay debitage. (1) Flake, (2) Blade with Siret fracture, (3) Laminar flake.

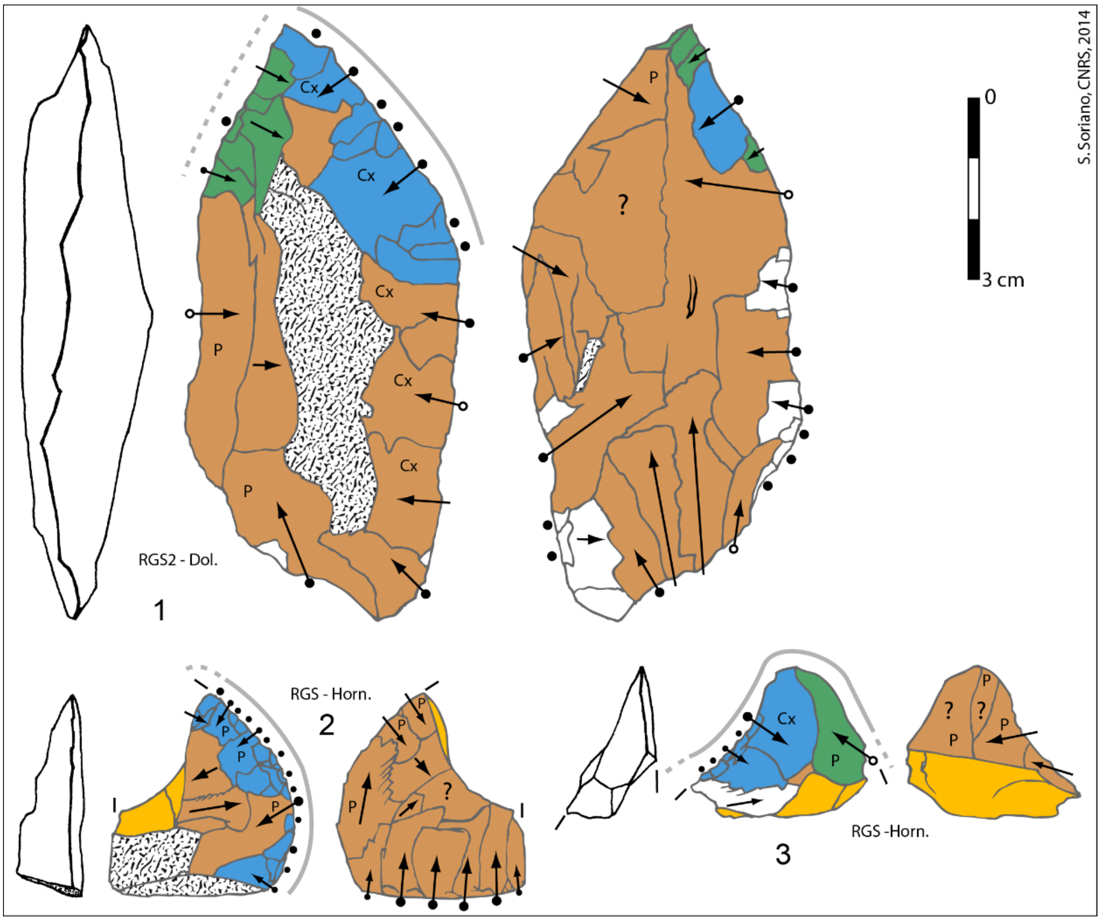

Figure F. Sibudu, Still Bay. Bifacial tools other than foliate points. (1) Bifacial piece, (2) Broken bifacial piece, (3) Distal fragment of bifacial piece.

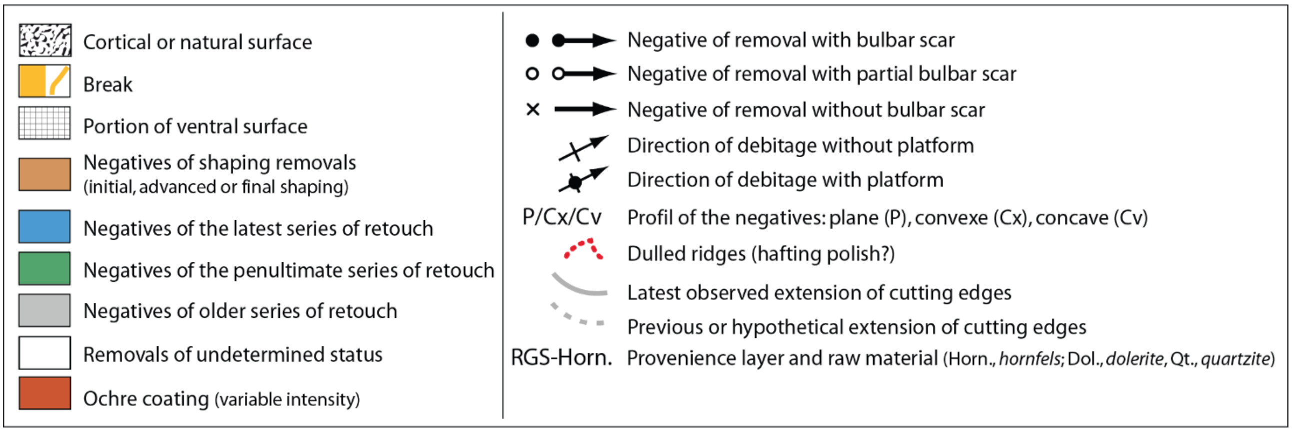

Figure F (*cont.*).

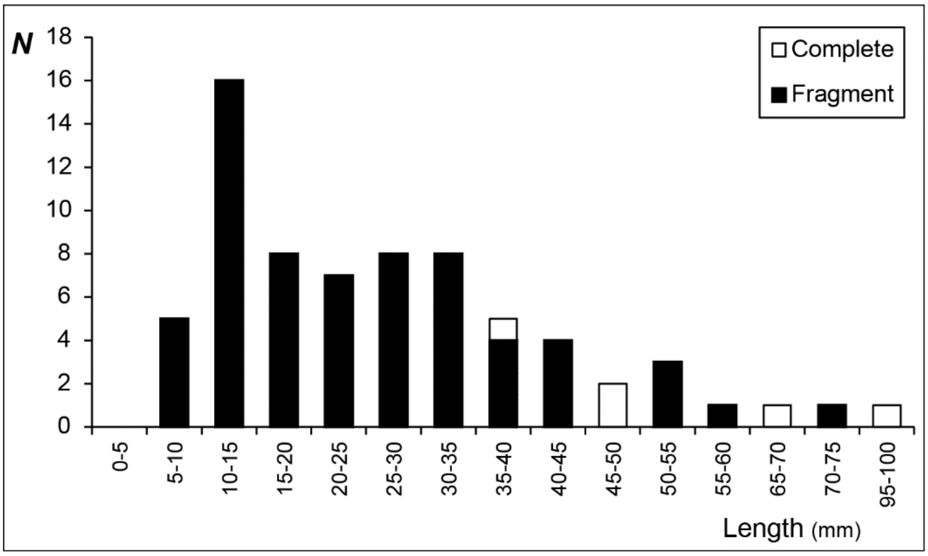

Figure G. Sibudu, Still Bay. Length distribution of bifacial pieces (complete and fragment). Tip flakes (chamfering flakes) are excluded.

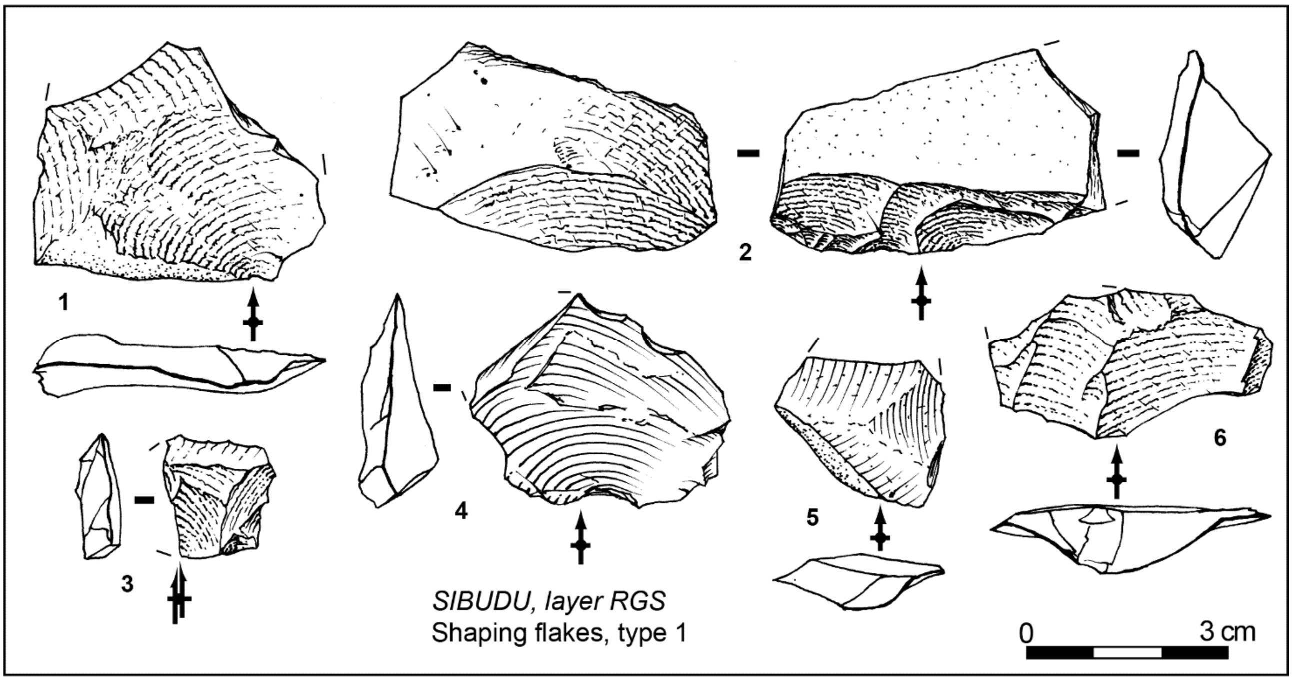

Figure H. Sibudu, layer RGS, Still Bay. Shaping flakes, type 1 (initial shaping flakes; [1]). The platform of flake n°2 is actually a portion of the ventral surface of a larger flake.

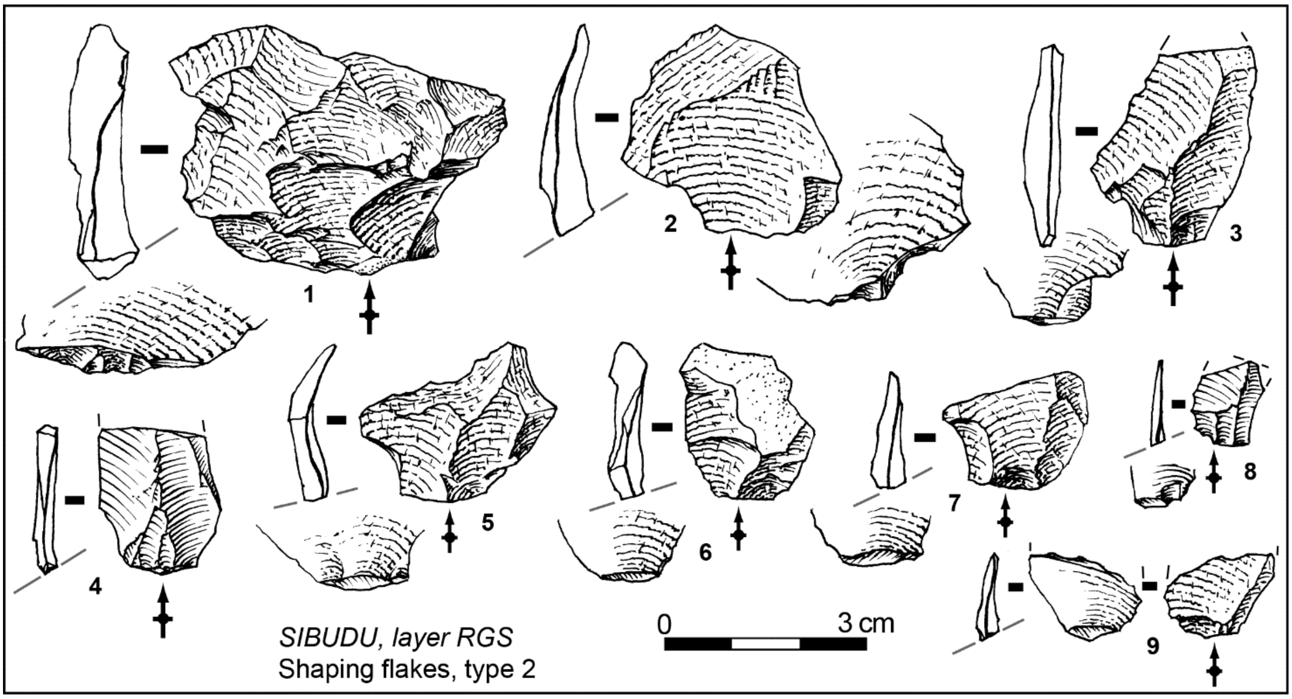

Figure I. Sibudu, layer RGS, Still Bay. Shaping flakes, type 2 (advanced shaping flake; [1]).

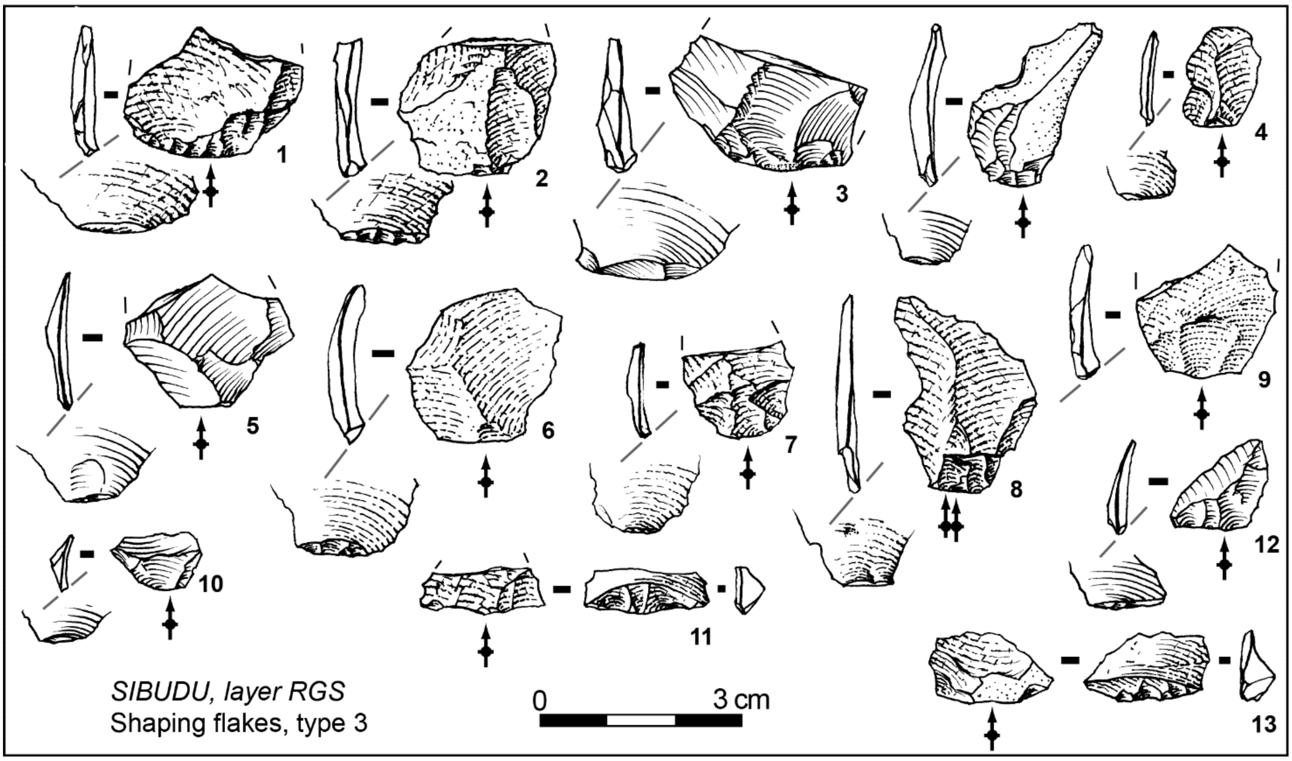

Figure J. Sibudu, layer RGS, Still Bay. Shaping flakes, type 3 (final shaping flakes; [1]).

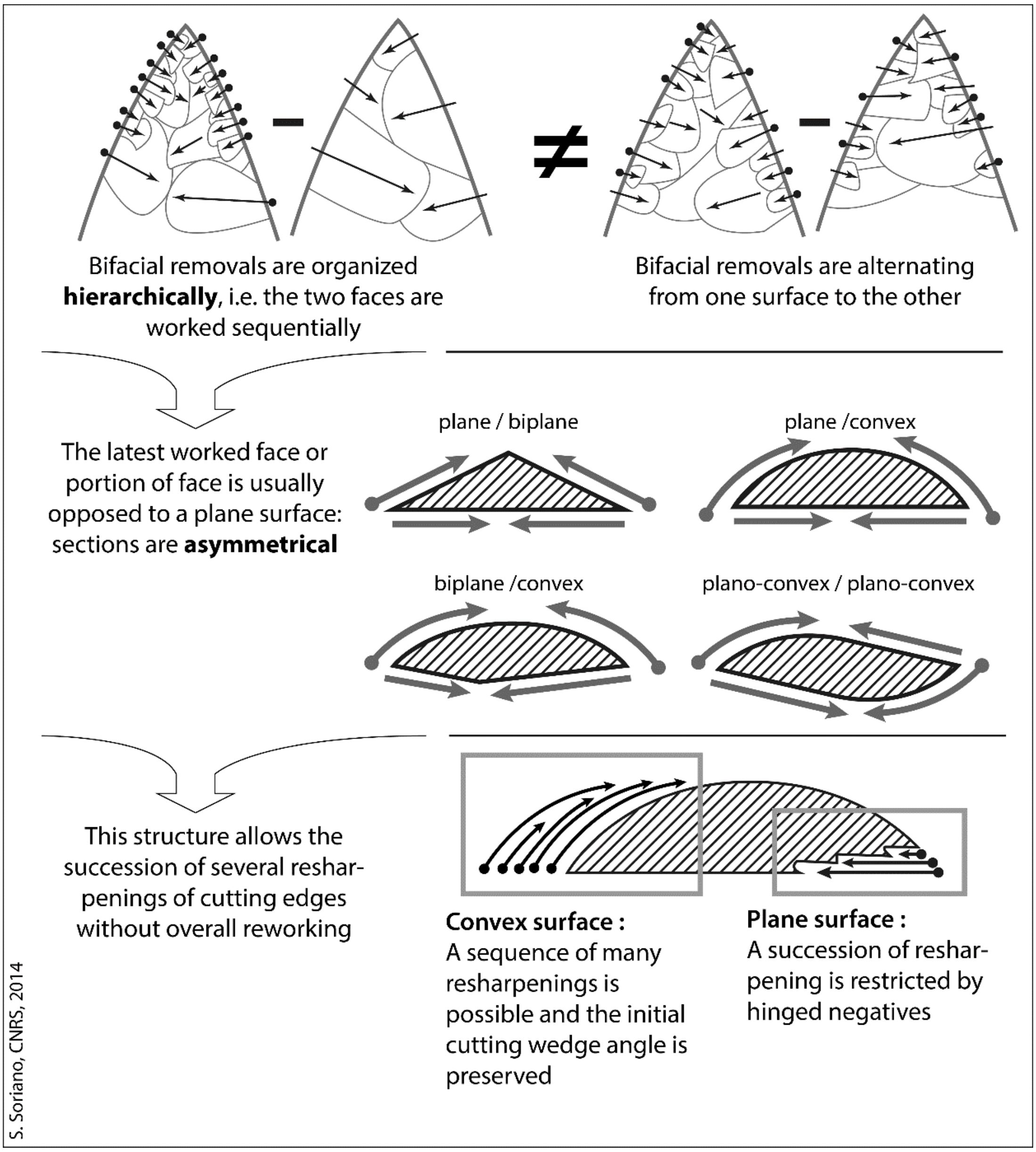

Figure K. Sibudu Still Bay bifacial points are characterized by removals organized hierarchically (ranked). Such a structure allows succession of many resharpenings with minimal reworking (modified from [2: fig. 2, 4]).

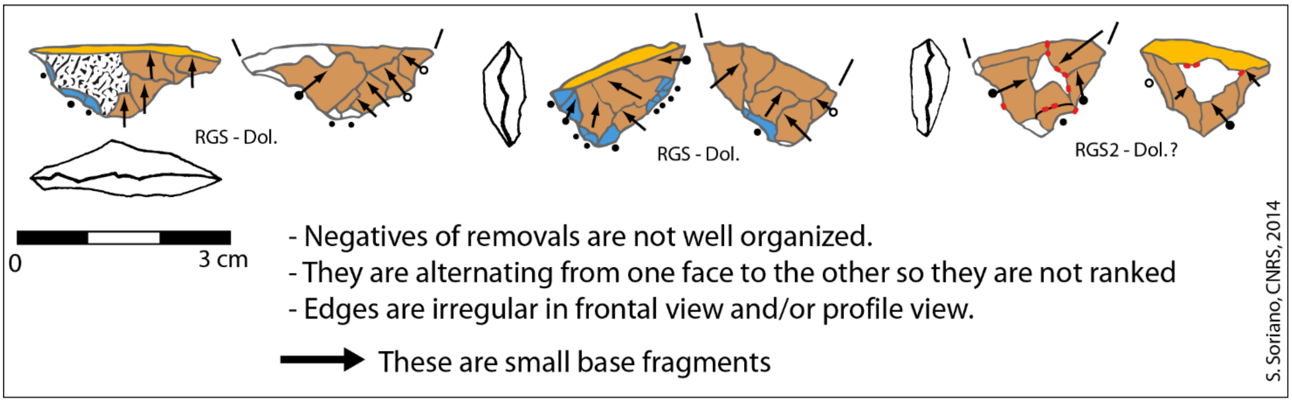

Figure L. Sibudu, Still Bay. On these small base fragments of bifacial pieces, removals are not well organized and faces are not ranked.

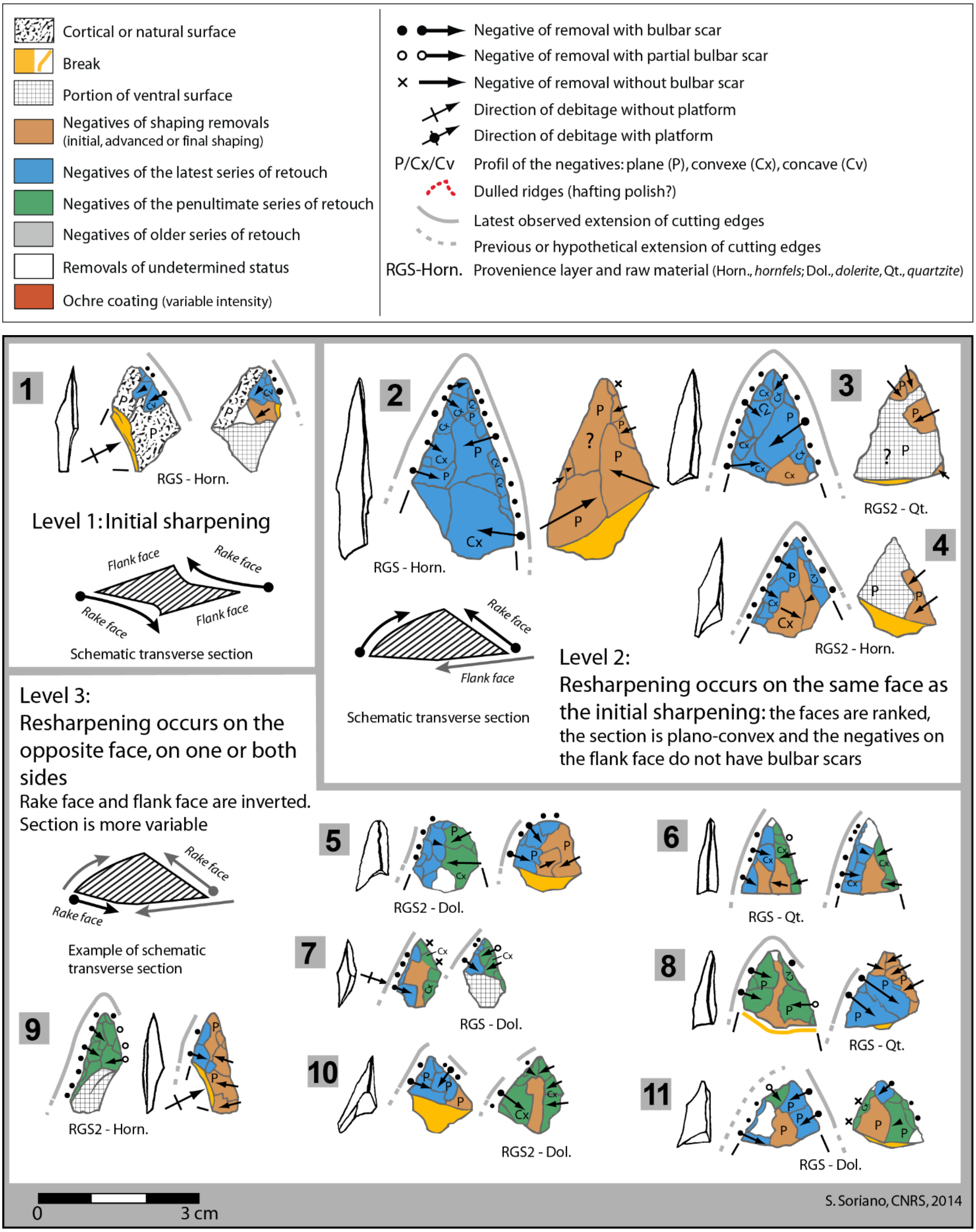

Figure M. Sibudu, Still Bay. Variations in complexity of removal organization on the distal part of bifacial pieces from Sibudu Still Bay are resulting from different degrees of sharpening or resharpening of bifacial points. Photos of (1, 3, 4, 5 7, 9, 10, 11) are provided in S1 File Figure B: 4, 9, 14, 3, 2, 1, 7, 8, photo of (2) is in S1 File Figure A: 4, and photos of (6, 8) are in Fig. 4: 11, 10.

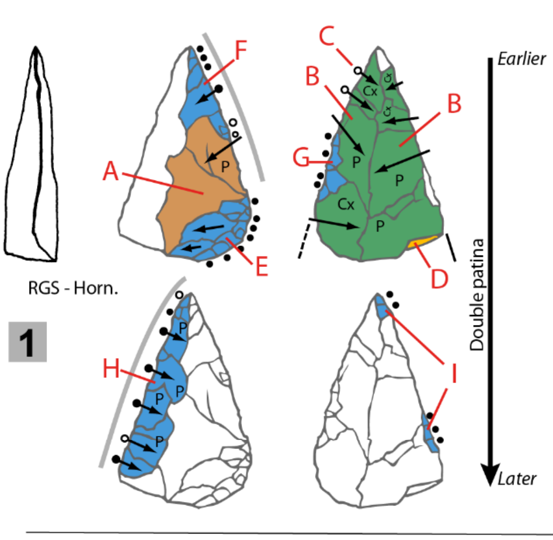

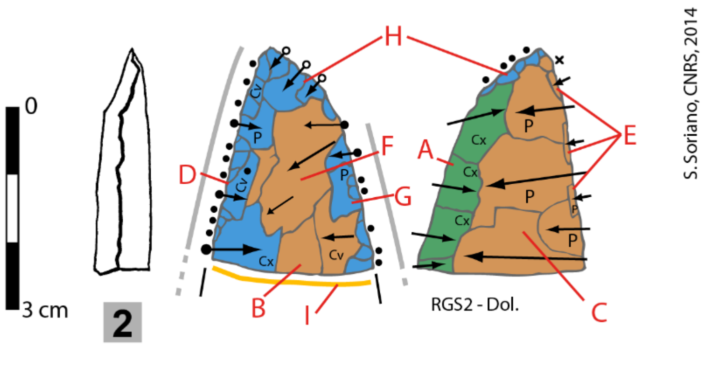

N° 1 - A small area of initial shaping scars is remaining (A) and the whole opposed surface is covered by sharpening removals (B). Negatives of bulbar scars from these removals are only partially and locally preserved (C). The cause of the fracture (D) of this originally longer bifacial foliate piece is unknown. The distal part is then reworked by trimming (E) the fracture edge while the cutting edge is resharpened by alternate removals (F then G). The ranking of surfaces is partly inverted. The piece is discarded (after use ?) and patina developed. After an undetermined gap of time, the tool is once again sharpened partly bifacially (H then I).

N°2 - (B) is the oldest surface with (A), a serie of negatives with a convex profile. These last are remnants of an initial sharpening phase (convex retouches on rake face). A new flank face is then built (C) with large flat removals. This new cutting edge was probably resharpened many times (D). In fact on the flank face, close to the edge, only few millimeters of the distal part of three negatives are preserved (E). This means that the bulbar scar negatives were at least 5 mm far from present edge. They have been truncated by several sharpening removals on the opposed rake face. Subsequently another flank face is shaped (F) and sharpening (G) follows. The old rake face (A) is used as flank face (inversion of hierarchy). After that the tip is reworked (bifacial truncation, H) suggesting that a fracture occured. The point is finally broken (I) and this fragment is discarded.

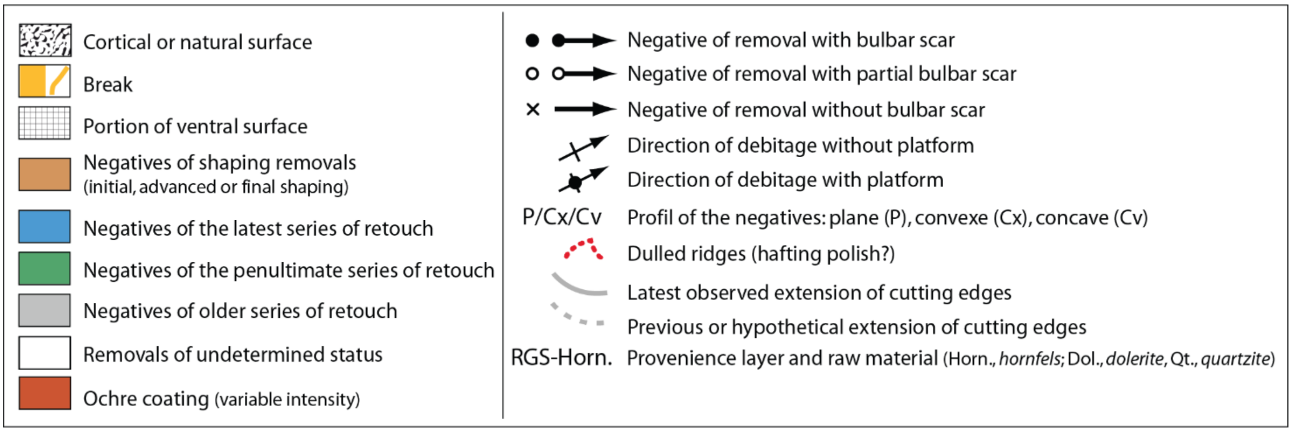

Figure N. Sibudu, Still Bay. Broken bifacial points bearing evidences of the sequence of resharpening and reworking. Double patina on (1) reveals the time gap between two phases of resharpening. (2) documents a major reduction through sequence of resharpening. Photos of (1) and (2) are respectively provided in S1 File Figure B: 10 and in S1 File Figure D: 6. (2) is described as a basal fragment of bifacial point in [3].

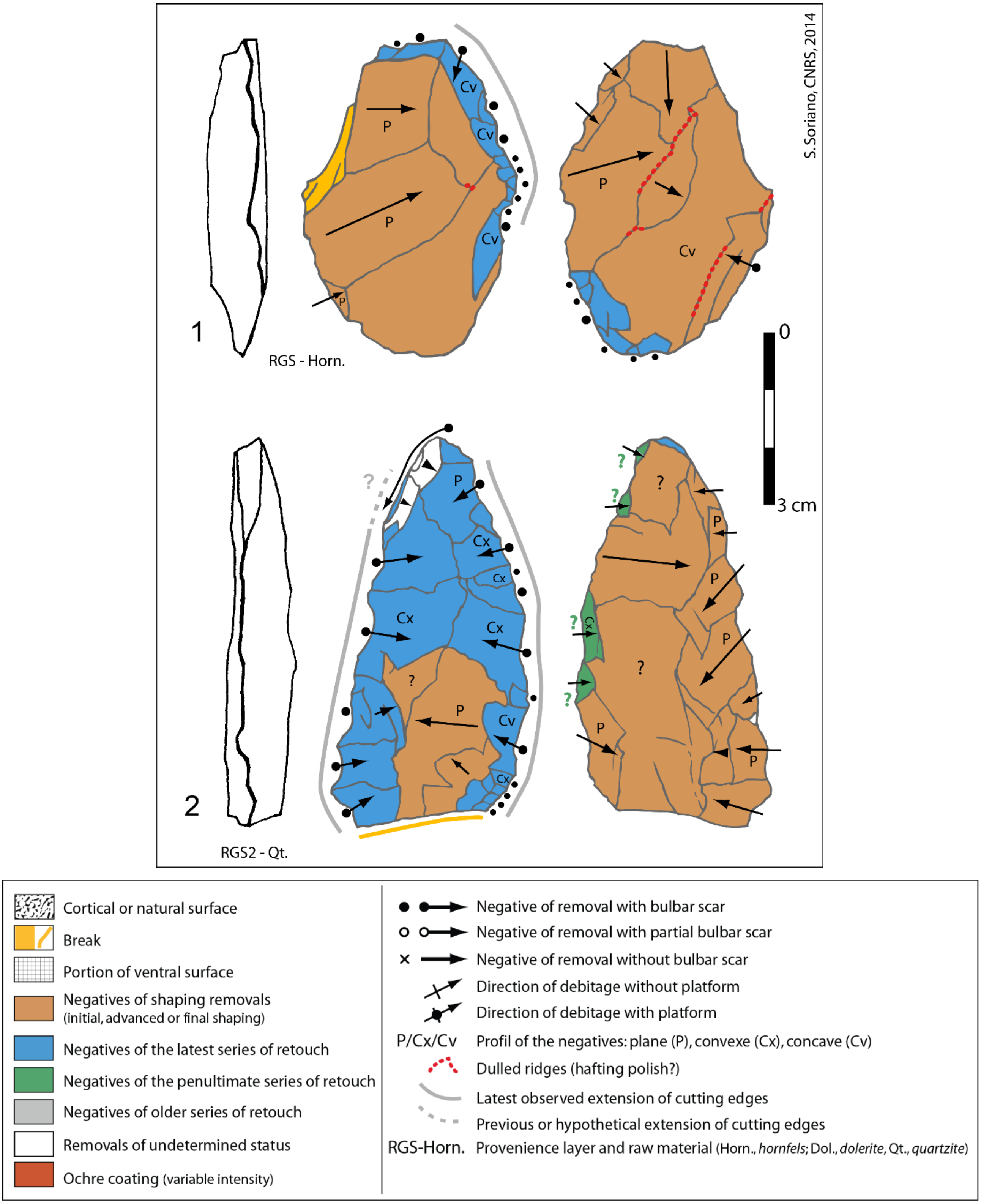

Figure O. Sibudu, Still Bay. Notching and denticulation is the ultimate level of resharpening of cutting edges from bifacial foliate pieces. Photos of (1) and (2) are respectively provided in S1 File Figure C: 2 and S1 File Figure A: 3.

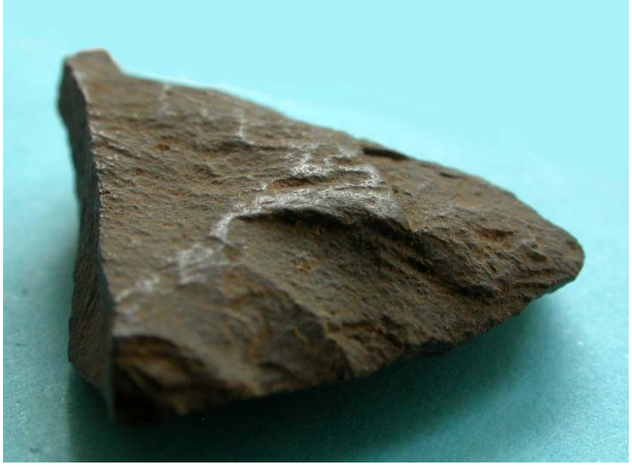

Figure P. Sibudu, Still Bay. Possible hafting macro-wear on a base from a dolerite broken bifacial piece from layer PGS. Oblique light reveals intensive smoothing of prominent ridges subsequently to the shaping but prior to fracture. Such feature is usually interpreted as the result of repeated but limited movements of the stone tool within his haft [4]. The strong development of this feature may be linked to the low hardness of dolerite.

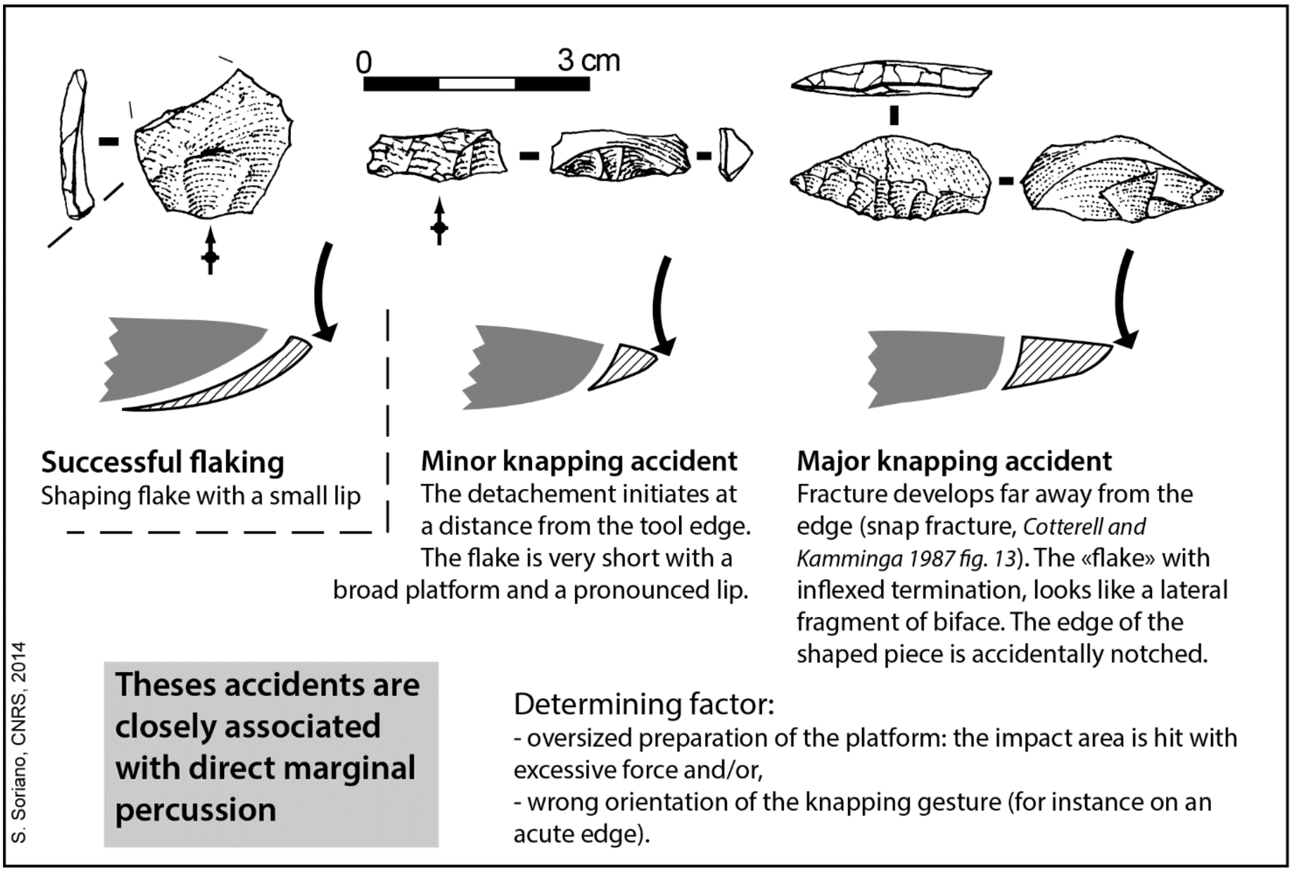

Figure Q. Sibudu, Still Bay. Some knapping accidents, even though uncommon, are quite diagnostic of the technique used for bifacial shaping, i.e. direct marginal percussion.

Features of fractures due to the knapping technique (pressure retouch *vs* percussion)

Among all the fractures (N=66) observed on the fragments of bifacial pieces, 20 have a definite direction (bending with feather termination, bending or flat lipped, Table N in S2 File): the more or less pronounced lip termination indicates the face towards which the constraint was oriented. The location of these fractures is polarized because 18 of these 20 occur on distal fragments (Figure M: 2, 4, 5, 10 in S1 File); more importantly their orientation is not random. In fact 16 are oriented towards the convex face of the bifacial point which in 11 cases was the last one to be retouched (Table N in S2 File). This indicates that these fractures occurred accidentally during the last attempt of sharpening. Moreover, this pattern of fracture is in agreement with the mechanical constraints associated with the percussion technique rather than the pressure technique (Figure R in S1 File).

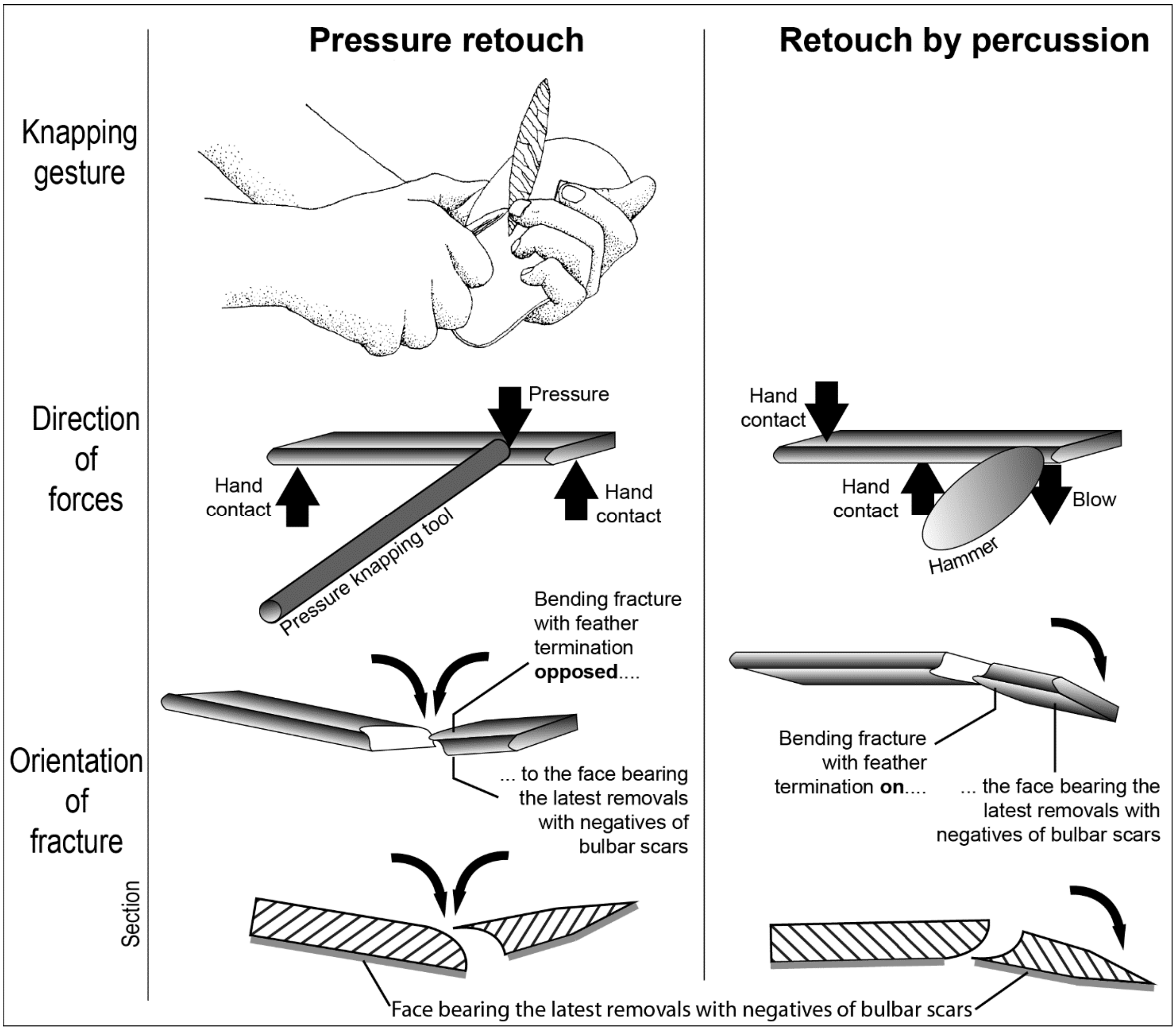

Figure R. Using pressure or percussion technique for final shaping and sharpening involve different knapping gesture, holding position and direction of forces. As a result, orientation of the bending fracture relative to the latest worked face (face bearing the latest retouchs with negative of bulbar scars) will depend on the knapping technique. Top left picture from [5].

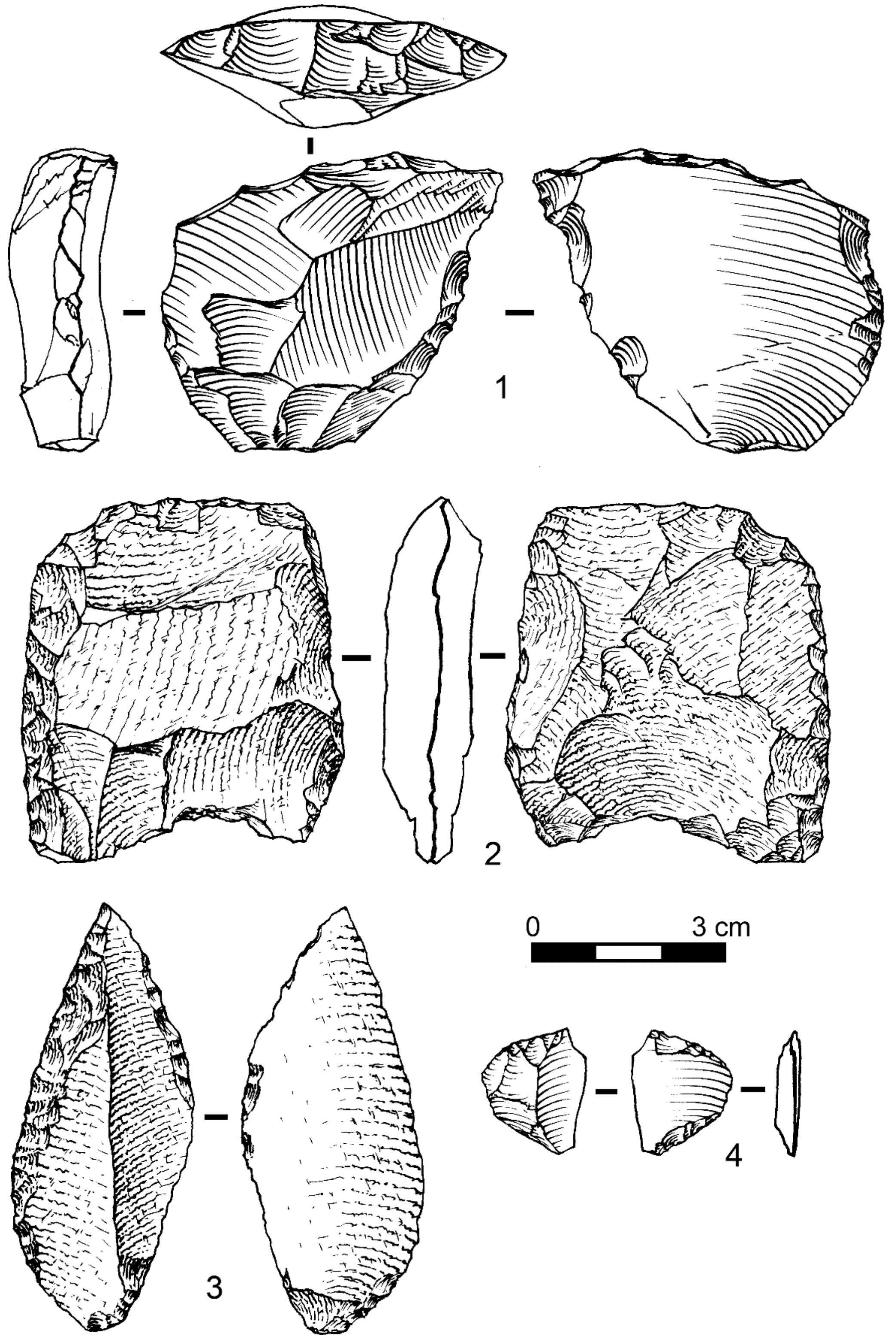

Figure S. Sibudu, Still Bay. Retouched tools. (1) *déjeté* scraper, RGS, B5b, hornfels, (2) scaled piece, RGS, B5c, dolerite, (3) unifacial point, RGS2, B5a, dolerite, (4) scaled piece, RGS, B5c, hornfels.

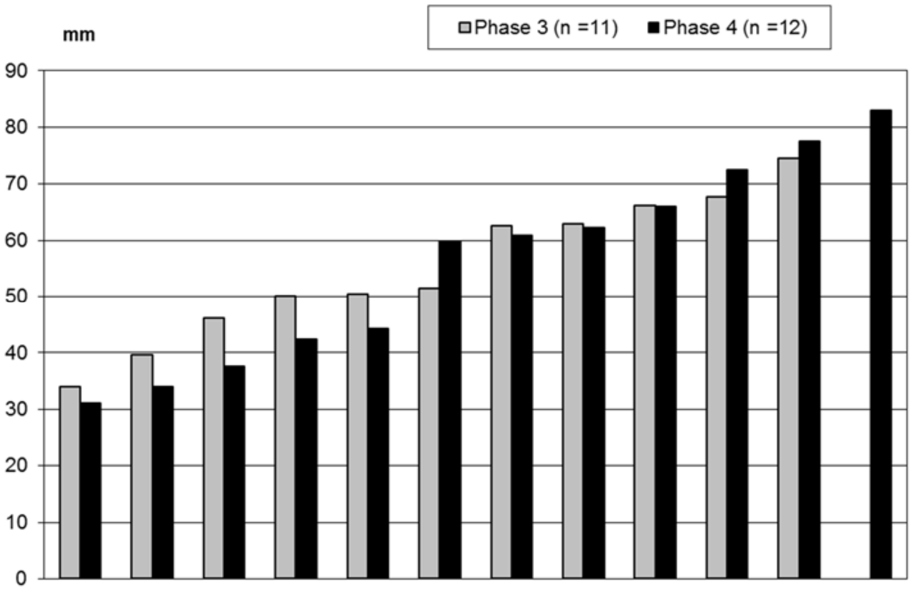

Figure T. Blombos Still Bay. Length of points phase 3 and 4.

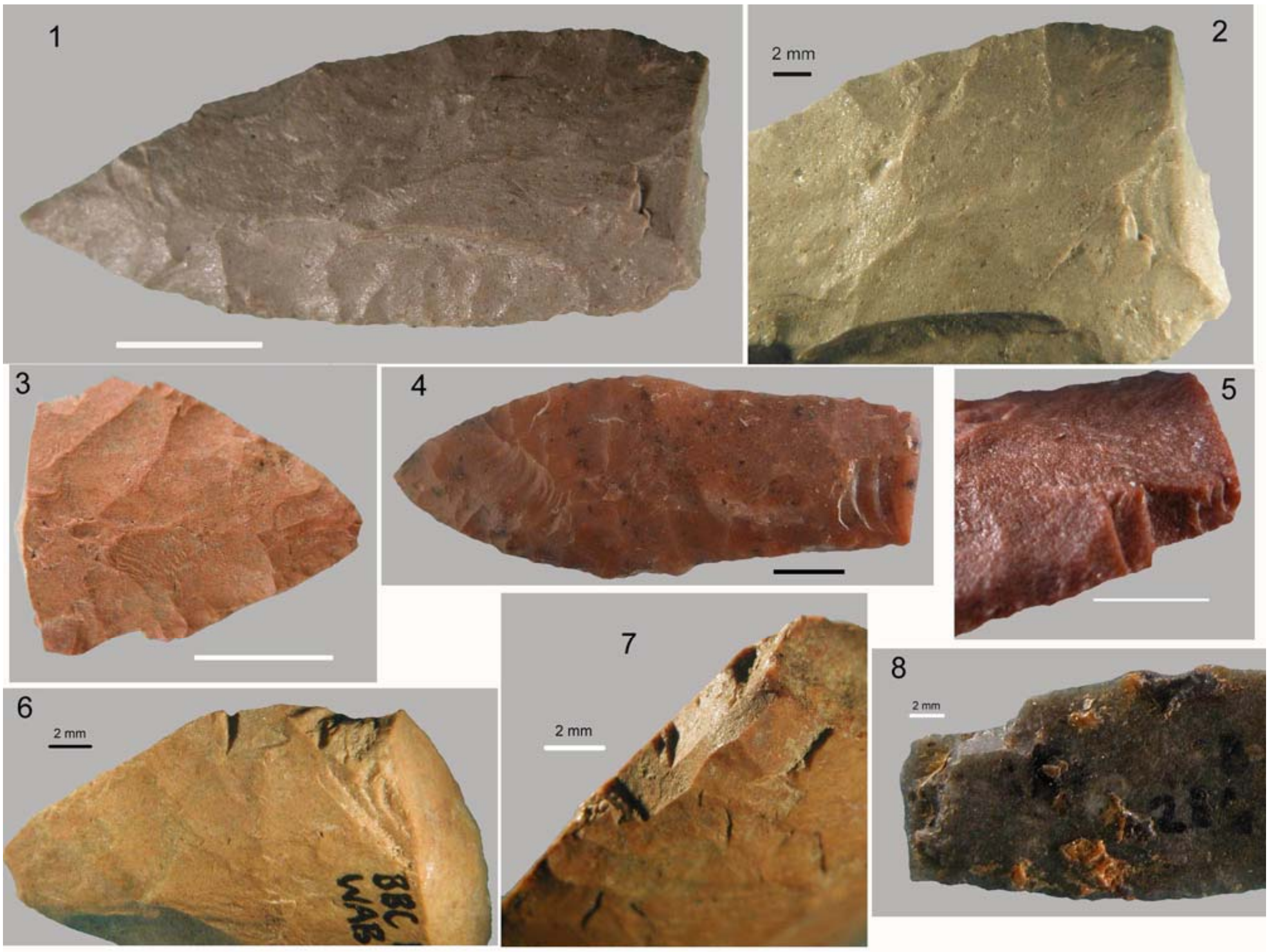

Figure U. Blombos Still Bay. Impact scars on the bases of two phase 3 Still Bay points, two Paleoindian and one experimental point. (1, 2) PVN 45 CF, step fracture; (3, 6, 7) two burin-like fracture at the base of PVN 97 WAB; (4) step fracture at the base of a Paleoindian point from the bison kill-site of Casper, Wyoming; (5) Burin-like fracture at the base of another Paleoindian point from the site of Casper; (8) Burin-like fracture on a point from the experimental series of [6].

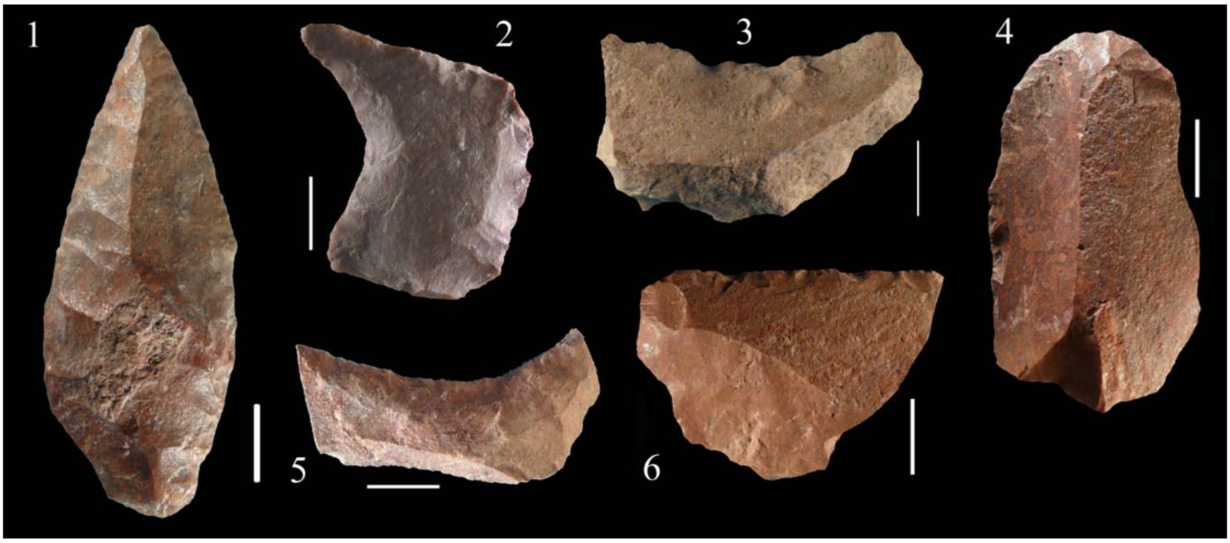

Figure V. Blombos Still Bay. (1) Unifacial point CF-CG no. 10; (2) Retouched notch/concave scraper CB no. 2262; (3) Retouched notch CB no. 32; (4) End scraper on laminar flake CD H6c; (5) Concave scraper CF no. 5; (6) Retouched flake CF no. 17. Scale bars = 1 cm.

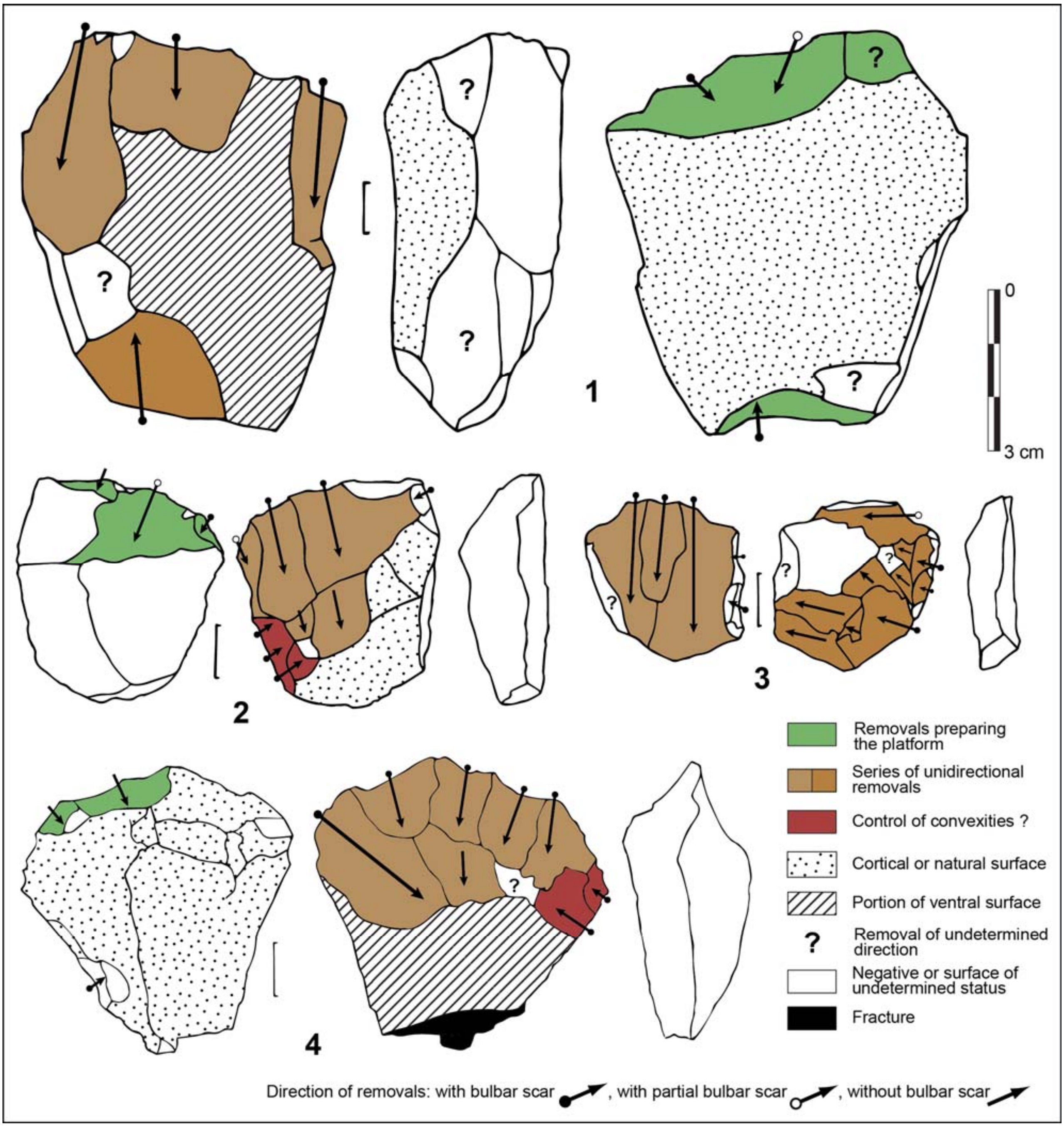

Figure W. Blombos Still Bay cores. (1) CD G6d L17; (2) CA 2283; (3) CCC H6c L 28; (4) CC 2131. All silcrete except 1 (quartzite).

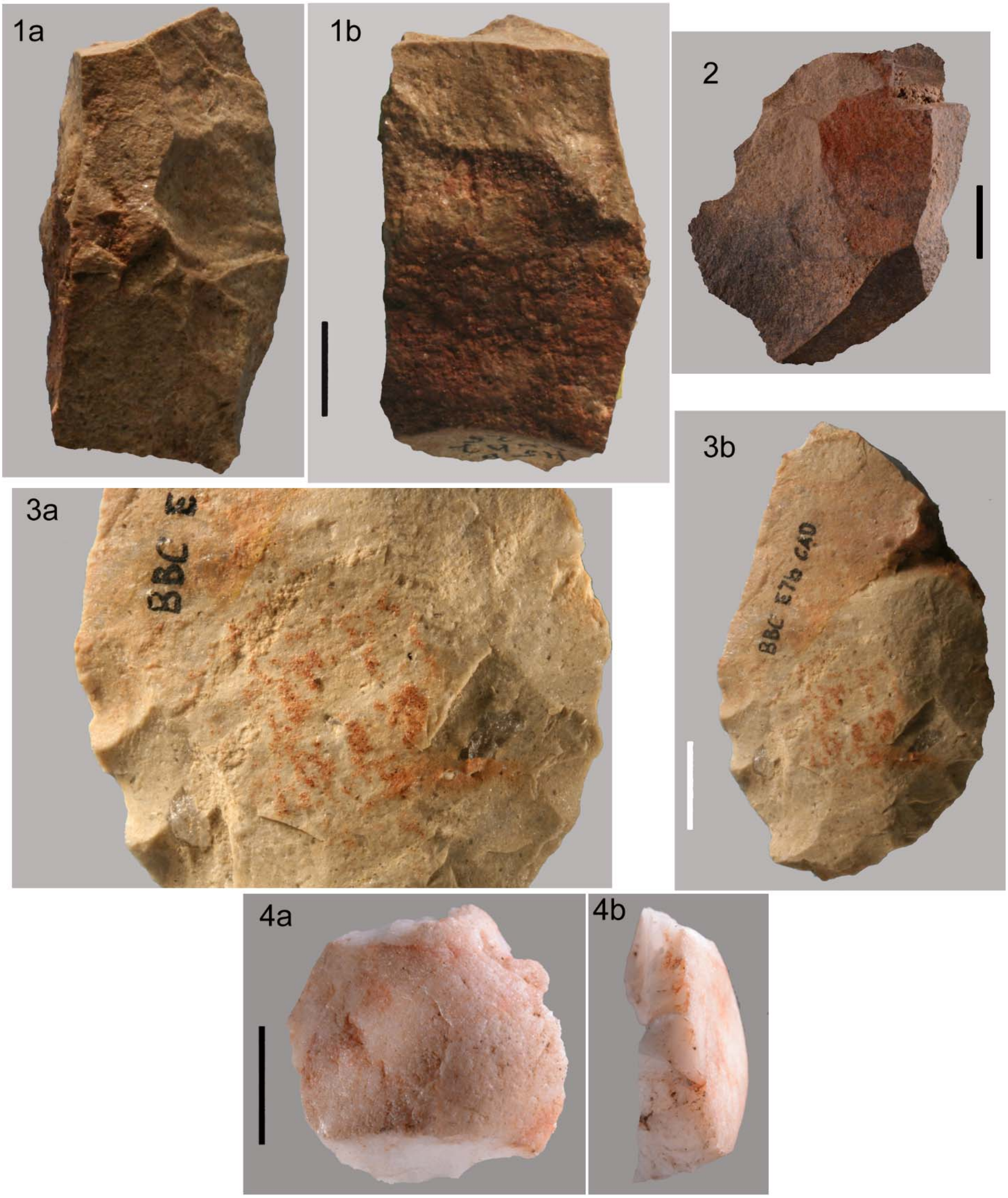

Figure X. Bombos Still Bay. Ochred pieces. (1a,b) Core fragment CD H2 H5b L25, ochre on 1b. (2) Hornfels flake CC 2223, ochre on the central scar of the dorsal face; (3a,b) Traces of ochre on the older surface of a phase 1 point, CAD E5b 25bis; (4a,b) Ochre on the cortical surface of a quartz core, CFD L16.

References

1. Soriano S, Villa P, Wadley L. Ochre for the toolmaker: Shaping the Still Bay points at Sibudu (KwaZulu-Natal, South Africa). J Afr Archaeol. 2009;7: 41–54. doi:10.3213/1612-1651-10121
2. Boëda E. Caractéristiques techniques des chaînes opératoires lithiques des niveaux micoquiens de Külna (Tchécoslovaquie). Les industries à pointes foliacées d'Europe centrale. Les Eyzies: Ed. Société des Amis du Musée National de Préhistoire et de la Recherche Archéologique; 1995. pp. 57–72.
3. Lombard M. First impressions of the functions and hafting technology of Still Bay pointed artefacts from Sibudu Cave. South Afr Humanit. 2006;18: 27–41.
4. Rots V. Wear Traces and the Interpretation of Stone Tools. J Field Archaeol. 2005;30: 61–73. doi:10.1179/009346905791072404
5. Inizan M-L, Reduron M, Roche H, Tixier J. Technologie de la pierre taillée. Meudon: CREP; 1995.
6. Geneste J-M, Plisson H. Technologie fonctionnelle des pointes à cran solutréennes : l'apport des nouvelles données de la Grotte de Combe-Saunières (Dordogne). In: Kozlowski JK, editor. Feuilles de pierre. Les industries à pointes foliacées du Paléolithique supérieur européen. Liège: Université de Liège; 1990. pp. 293–320.
