# Supplementary material for: The Still Bay and Howiesons Poort at Sibudu and Blombos: Understanding Middle Stone Age Technologies
Source: PLoS One. 2015 Jul 10;10(7):e0131127. doi: 10.1371/journal.pone.0131127 (PMC4498762; doi:10.1371/journal.pone.0131127)
Supplement: S2 File — (PDF) [file pone.0131127.s002.pdf]

The Still Bay and Howiesons Poort at Sibudu and Blombos: Understanding Middle Stone Age technologies

Sylvain Soriano, Paola Villa, Anne Delagnes, Ilaria Degano, Luca Pollarolo, Jeannette J. Lucejko, Christopher Henshilwood, Lyn Wadley

Supporting Information

S2 File

(Tables A-N)

Table A. Sibudu, Still Bay. Count of blades and elongated flakes.

Blade type	*N*
Crested blade with one or two prepared versants	2
Blade direcly underlying a crested blade	1
Totally cortical blade	1
Blade with a lateral cortical edge or cortical back	15
Blade with lateral and distal cortical edges	1
Blade with distal cortical edge	1
Blade from the optimal phase of debitage, without cortex	15
Other type of blade and undetermined type	5
Total	41

Table B. Sibudu, Still Bay. Frequencies of shaping flakes with cortex in a sample of layers RGS, HinRGS. Frequencies are not provided for less abundant materials.

% of shaping flakes with cortex			
Sample of layers RGS, HinRGS (N=584)	Dolerite	Hornfels	All raw materials
	8,9	3,9	8,6

Table C. Sibudu, Still Bay. Base shape of bifacial points (phases of manufacture 2 and 3 only). Types as in [1].

Base shape	*N*
Narrow V-shaped	1
Truncated elliptical	0
Elliptical	1
Wide arched	4
Broad	5
Irregular	2
Indeterminate	3
Total	16

Table D. Sibudu, Still Bay. Attributes of the shaping flake types. See Figures H-J in S1 File for illustrations of each type. (1). See [2] for description of platform type.

**Technical attributes**	**Shaping flake types**		
	Type 1	Type 2	Type 3
Production stage	Initial shaping	Advanced shaping	Final shaping (thinning, regularizing and maintenance)
Thickness	Medium to thick	Thin	Thin
Morphology	Variable	Variable	Parallel or divergent sides
Dominant platform type (1)	Wide quadrangular or trapezoidal	Narrow linear	Narrow linear
Platform thickness	Frequently thick	Thick or thin	Thin
Bulb morphology (1)	Prominent bulb generally without a lip	Generally lipped without a bulb	Always lipped without a bulb
Profile	Sometimes curved	Frequently curved	Frequently curved
Cortex on dorsal surface	Frequent	Residual	Absent or residual
Exterior platform angle	High	Variable	Low
Number of removals on dorsal surface	Rare	Variable numbers	Generally a high number of removals

Table E. Sibudu, Still Bay. Counts of shaping flake types by raw material.

Layers RGS, HinRGS, RGSunderRock, RGS2	Dolerite	Hornfels	Quartzite	Sandstone	Quartz	Crypto crystalline silica	Undetermined	Total	%
Initial shaping flakes (type 1)	136	26	9	22	17	0	0	210	9,7
Advanced shaping flakes (type 2)	909	331	47	23	16	4	9	1339	61,8
Final shaping flakes (type 3)	336	234	32	3	4	0	10	619	28,6
Total	1381	591	88	48	37	4	19	2168	100

Table F. Sibudu, Still Bay. Frequency of dorsal cortex (or natural surface) on shaping flake (Sample of layer RGS). Undetermined cases excluded.

	With cortex	Without cortex	Total
Initial shaping flakes (type 1, N= 75)	38,7%	61,3%	100%
Advanced shaping flakes (type 2, N=336)	5,7%	94,3%	100%
Final shaping flakes (type 3, N=172)	1,2%	98,8%	100%
Total	8,6%	91,4%	100%

Table G. Sibudu, Still Bay. Frequency distribution of platform types on shaping flakes (Sample of layer RGS,). Undetermined platforms excluded. Platform types as in [2].

Platform type	Cortical or natural	Plain	Facetted + dihedral	Ridge	Undet.	Total
Initial shaping flakes (type 1, N=73)	31,5%	35,6%	9,6%	9,6%	13,7%	100%
Advanced shaping flakes (type 2, N=334)	8,1%	51,2%	20,1%	10,5%	10,2%	100%
Final shaping flakes (type 3, N=174)	1,7%	39,1%	47,7%	2,3%	9,2%	100%

Table H. Sibudu, Still Bay. Mean and standard deviation of length and width for complete or almost complete shaping flakes (Sample of layer RGS).

	Shaping flake type	Initial	Advanced	Final
N *(complete or almost complete)*		32	91	59
Length (mm)	Mean	24,3	16,9	16,4
	SD	11,9	6,3	5,3
Width (mm)	Mean	24,2	16,0	16,2
	SD	11,2	5,7	5,9

Table I. Sibudu, Still Bay. Frequency distribution of platform morphology of shaping flakes (Sample of layer RGS). Flakes with undetermined, broken or splited platforms are excluded. Platform morphological types as in [2].

Platform morphology	Wide	Winged	Oval, triangular	Narrow linear	Puncti-form	Shattered	Total
Initial shaping flakes (type 1, N=74)	62,2%	2,7%	9,5%	10,8%	4,1%	10,8%	100%
Advanced shaping flakes (type 2, N=333)	12,0%	7,8%	20,4%	51,1%	2,4%	6,3%	100%
Final shaping flakes (type 3, N=173)	2,3%	5,2%	5,8%	81,5%	2,9%	2,3%	100%

Table J. Sibudu, Still Bay. Frequency distribution of platform edge abrasion on shaping flakes (Sample of layer RGS). Flakes with undetermined, broken or split platforms are excluded.

	Abrasion of platform edge	No abrasion of platform edge	Total
Initial shaping flakes (type 1)	0%	100%	100%
Advanced shaping flakes (type 2)	7,8%	92,2%	100%
Final shaping flakes (type 3)	39,8%	60,2%	100%

Table K. Sibudu, Still Bay. Frequency distribution of profile curvature of shaping flakes. (Sample of layer RGS). Flakes with undetermined profile curvature are excluded.

	Curved profile	Not curved	Total
Initial shaping flakes (type 1)	34,2%	65,8%	100%
Advanced shaping flakes (type 2)	38,5%	61,5%	100%
Final shaping flakes (type 3)	67,8%	32,2%	100%

Table L. Sibudu, Still Bay. Presence of hierarchized faces according to bifacial fragment type (48 fragments, 5 tip flakes). Complete or almost complete pieces are excluded.

Hierarchy of face	Distal fragment	Proximal or lateral fragment
Faces are hierarchised: last shaping removals and/or retouches are on the same convex face or are on alternates surfaces	33	2
Faces are not hierarchised: last shaping removals and/or retouches are alternating from one face to another	1	8
Hierarchy of faces is undetermined	2	7

Table M. Sibudu, Still Bay. Degree of resharpening of cutting edges from bifacial pieces. Levels are defined in Figure 6.

**Degree of resharpening**		*N*
Level 1	Cutting edge exhibits initial sharpening features but was not resharpened	3
Level 2	Cutting edge was resharpened (negatives of removals on the flank face are deeply truncated by sharpening removals -*ie* retouchs - on the rake face)	21
Level 3	Cutting edge was reworked (sharpening moved to the opposite surface; rake and flank faces are inverted)	8
Level 4	A final sharpening is applied through denticulation of the cutting edge	3
	Other	1
	Undetermined	13
	Non applicable (absence of cutting edges)	9
	Total	58

Table N. Sibudu, Still Bay. Orientation of bifacial piece fractures relative to their morphology.

	**Orientation of the fracture**					
**Type of fracture**	Towards a convex face bearing the latest retouchs/ removals	Towards a convex face bearing retouchs (1)	Towards a flat face	Undeter - mined	Not applicable (non oriented fracture)	Total
Bending with feather termination	**4**	**2**		2		8
Bending	**1**	**3**				4
Flat lipped	**7**		1			8
Irregular					15	15
Flat orthogonal					20	20
Other					2	2
Fire					4	4
Undetermined					3	3
Cone fracture				1	1	2
No fracture					11	11
Total	11	5	1	3	56	77

(1) but there are also retouchs on the opposed face

References

1. Villa P, Soressi M, Henshilwood CS, Mourre V. The Still Bay points of Blombos Cave (South Africa). J Archaeol Sci. 2009;36: 441–460.
2. Soriano S, Villa P, Wadley L. Blade technology and tool forms in the Middle Stone Age of South Africa: the Howiesons Poort and post-Howiesons Poort at Rose Cottage Cave. J Archaeol Sci. 2007;34: 681–703.
