# Supplementary material for: The Still Bay and Howiesons Poort at Sibudu and Blombos: Understanding Middle Stone Age Technologies
Source: PLoS One. 2015 Jul 10;10(7):e0131127. doi: 10.1371/journal.pone.0131127 (PMC4498762; doi:10.1371/journal.pone.0131127)
Supplement: S3 File — (PDF) [file pone.0131127.s003.pdf]

The Still Bay and Howiesons Poort at Sibudu and Blombos: Understanding Middle Stone Age technologies

Sylvain Soriano, Paola Villa, Anne Delagnes, Ilaria Degano, Luca Pollarolo, Jeannette J. Lucejko, Christopher Henshilwood, Lyn Wadley

Supporting Information

S3 File

Materials and Methods

The present study of the HP and SB lithic assemblages from Sibudu Cave was designed in the same way as our previous studies [1–6]. The aim is to build an overview of lithic production, use and discard and their changes through time.

The Still Bay at Sibudu
Sorting procedures. All the shaping flakes (N=2169) coming from the excavated seven quadrants within squares B5, B6 and C5 from layers RGS (including HinRGS, RGSunderRock) and RGS2 were sorted by type and raw material. Debitage other than shaping byproducts (blades and flakes, N=55) and retouched tools (N=53) from the same seven quadrants were analysed. All the shaping flakes from two quadrants (B5a, B5c) were selected (N=583) to be analysed with further details (measurement, profile curvature, platform type, platform preparation). The sample of bifacial pieces from theses seven quadrants was enlarged with pieces excavated in 2009 from square B4, C4, C5 and C6 from the same layers, and with pieces from layer PGS (squares B5, B6, C5, C6) to reach a total of 77.

Methods. We develop a technological approach to reconstruct the reduction sequences from raw material procurement to tool manufacture [7,8]. Flakes produced through debitage were first extracted with reference to flake productions usually encountered in South-African MSA [3,9–11]. The remaining flakes were considered resulting from bifacial shaping according to descriptions in [8,12,13]. The manufacturing phases of points were identified as in [4]. The shaping flakes were classified according to the experimental pattern of shaping bifacial foliate pieces from [14]. Bifacial shaping is a continuous process (this is a major difference with debitage), meaning that any classification of shaping flakes introduces somewhat artificial breaks. Our three types of shaping flakes (1, 2 and 3) are designed to correspond to the shaping by-products resulting from the successive stages of Callahan: initial blank shaping (= initial edging in [14]), advanced shaping (= primary thinning in [14]) and final shaping (thinning,

regularizing and maintenance = secondary thinning plus shaping in [14]) (Table D in S2 File, Figures H-J in S1 File). There is no sharp separation between these three phases, as is true for the whole bifacial shaping process: we observe a progressive passage from one flake type to another. Each trait alone is insufficient for the classification of a flake; it is the combination of attributes that is pertinent.

Reduction and recycling of Still Bay bifacial points may have masked previous shape and function, as McCall and Thomas [15] suggested. Thus we paid particular attention to the organization and the chronology of the shaping (final shaping, retooling, reworking) to reconstruct the manufacturing stages. For each bifacial piece, complete or broken, the chronology of flaking was analyzed as in [16] and illustrated, as in [8,17]. We used a techno-functional method of analysis developed for stone tools of the Early and Middle Palaeolithic in Europe [18,19] and recently applied to the post-HP of Sibudu [20]. This method is derived from the geometric design of modern cutting tools [21,22] and supported by theories of technical evolution [23,24].

Terms used to describe geometry of cutting tools, especially industrial metal working tools, have been in use for quite some time. According to [21,22] the designed components of the cutting tool are defined as follows:

- *Rake face (or top surface)* is the surface over which the chip, formed in the cutting process, slides,
- *Flank face (or relief surface)* is the surface which face the machined surface of the workpiece,
- Rake and flank faces are the *working surfaces*,
- The intersection of the working surfaces form the *cutting edge*,
- *Cutting wedge* is the tool body enclosed between the rake and the flank surfaces.

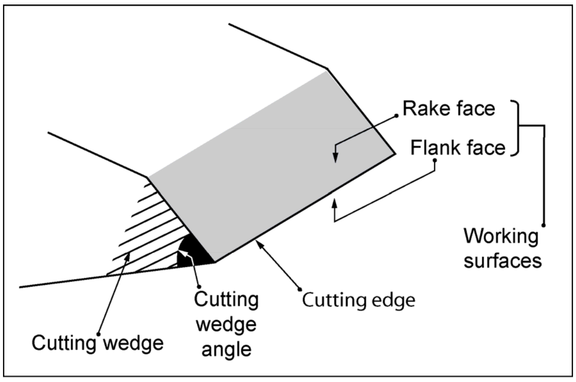

Terms used to describe the geometry of the cutting edge of a tool

The working surfaces of a tool are usually hierarchized and this hierarchy is structurally determined both by mechanical constraints the tool is exposed to when working, and by the technical constraints of the sharpening. Most often sharpening occurs only on the rake face (ie. on a wood chisel, resharpening is applied on the bevelled edge).The angle of the cutting edge is determined both by the hardness of the workpiece and tool motion. The

mechanical constraints associated with modern metal cutting tools are also valuable in the analysis of Palaeolithic stone tools even if the worked materials and the materials of tools themselves are different. Describing and analyzing the cutting edges of Palaeolithic stone tools with respect of this geometry and structure help to understand patterns of knapping scars (chronology, hierarchy, extension, orientation) and to interpret them in terms of sharpening and resharpening procedures. See text figure 6 for an application of this method of description and analyse.

References

1. Soriano S, Villa P, Wadley L. Blade technology and tool forms in the Middle Stone Age of South Africa: the Howiesons Poort and post-Howiesons Poort at Rose Cottage Cave. J Archaeol Sci. 2007;34: 681–703.

2. Soriano S, Villa P, Wadley L. Ochre for the toolmaker: Shaping the Still Bay points at Sibudu (KwaZulu-Natal, South Africa). J Afr Archaeol. 2009;7: 41–54.

3. Villa P, Delagnes A, Wadley L. A late Middle Stone Age artifact assemblage from Sibudu (KwaZulu-Natal) : comparisons with the European Middle Paleolithic. J Archaeol Sci. 2005;32: 399–422.

4. Villa P, Soressi M, Henshilwood CS, Mourre V. The Still Bay points of Blombos Cave (South Africa). J Archaeol Sci. 2009;36: 441–460.

5. Villa P, Soriano S, Teyssandier N, Wurz S. The Howiesons Poort and MSA III at Klasies River main site, cave 1A. J Archaeol Sci. 2010;37: 630–655.

6. Villa P, Soriano S, Tsanova T, Degano I, Higham TF, d' Errico F, et al. Border Cave and the beginning of the Later Stone Age in South Africa. Proc Natl Acad Sci U S A. 2012;109: 13208–13213.

7. Boëda E, Geneste J-M, Meignen L. Identification des chaînes opératoires lithiques du Paléolithique ancien et moyen. Paléo. 1990;2: 43–80.

8. Inizan M-L, Reduron-Ballinger M, Roche H, Tixier J. Technology and Terminology of Knapped Stone. Nanterre: Cercle de Recherches et d'Etudes Préhistoriques; 1999.

9. Volman TP. Early prehistory of southern Africa. In: Klein RG, editor. Southern African prehistory and palaeoenvironments. Rotterdam: Balkema; 1984. pp. 169–220.

10. Conard NJ, Soressi M, Parkington JE, Wurz S, Yates R. A unified lithic taxonomy based on patterns of core reduction. South Afr Archaeol Bull. 2004;59: 12–16.

11. Wurz S. The Middle Stone Age at Klasies River, South Africa. PhD, University of Stellenbosch. 2000.

12. Bradley B, Sampson CG. Analysis by replication of two Acheulian artifact assemblages. In: Bailey G, Callow P, editors. Stone Age Prehistory: Studies in Memory of Charles McBurney. Cambridge: Cambridge University Press; 1986. pp. 29–45.

13. Newcomer MH. Some Quantitative Experiments in Handaxe Manufacture. World Archaeol. 1971;3: 85–104.

14. Callahan E. The basics of biface knapping in the Eastern fluted point tradition: a manual for flintknappers and lithic analysts. Archaeol East N Am. 1979;7: 1–180.

15. McCall GS, Thomas JT. Still Bay and Howiesons Poort Foraging Strategies: Recent Research and Models of Culture Change. Afr Archaeol Rev. 2012;29: 7–50.

16. Richter J. Copies of flakes: operationnal sequences of foliate pieces from Buran Kaya III level B1. In: Chabai VP, Monigal K, Marks AE, editors. The Paleolithic of Crimea, III. The Middle Paleolithic and Early Upper Paleolithic of Eastern Crimea. Liège: Université de Liège; 2004. pp. 233–247.

17. Dauvois M. Précis de dessin dynamique et structural des industries lithiques préhistoriques. Périgueux: Fanlac; 1976.

18. Boëda E. Détermination des unités techno-fonctionnelles de pièces bifaciales provenant de la couche acheuléenne C'3 base du site de Barbas I. In: Cliquet D, editor. Les industries à outils bifaciaux du Paléolithique moyen d'Europe occidentale. Liège: Université de Liège; 2001. pp. 51–75.

19. Soriano S. Statut fonctionnel de l'outillage bifacial dans les industries du Paléolithique moyen: propositions méthodologiques. In: Cliquet D, editor. Industries à outils bifaciaux du Paléolithique moyen d'Europe occidentale. Liège: Université de Liège; 2001. pp. 77–83.

20. Conard N, Porraz G, Wadley L. What is in a name? Characterising the "Post-Howieson"s Poort' at Sibudu. South Afr Archaeol Bull. 2012;67: 180–199.

21. Astakhov VP. Geometry of Single-point Turning Tools and Drills: Fundamentals and Practical Applications. Springer; 2010.

22. Astakhov VP. Tribology of Metal Cutting. Elsevier; 2006.

23. Simondon G. Du mode d'existence des objets techniques. Paris: Ed. Aubier; 1958.

24. Rabardel P. Les hommes et les technologies. Approche cognitive des instruments contemporains. Paris: Armand Colin; 1995.
