# Supplementary material for: The Still Bay and Howiesons Poort at Sibudu and Blombos: Understanding Middle Stone Age Technologies
Source: PLoS One. 2015 Jul 10;10(7):e0131127. doi: 10.1371/journal.pone.0131127 (PMC4498762; doi:10.1371/journal.pone.0131127)
Supplement: S4 File — (PDF) [file pone.0131127.s004.pdf]

The Still Bay and Howiesons Poort at Sibudu and Blombos: Understanding Middle Stone Age technologies

Sylvain Soriano, Paola Villa, Anne Delagnes, Ilaria Degano, Luca Pollarolo, Jeannette J. Lucejko, Christopher Henshilwood, Lyn Wadley

Supporting Information

S4 File

(Figures A-O)

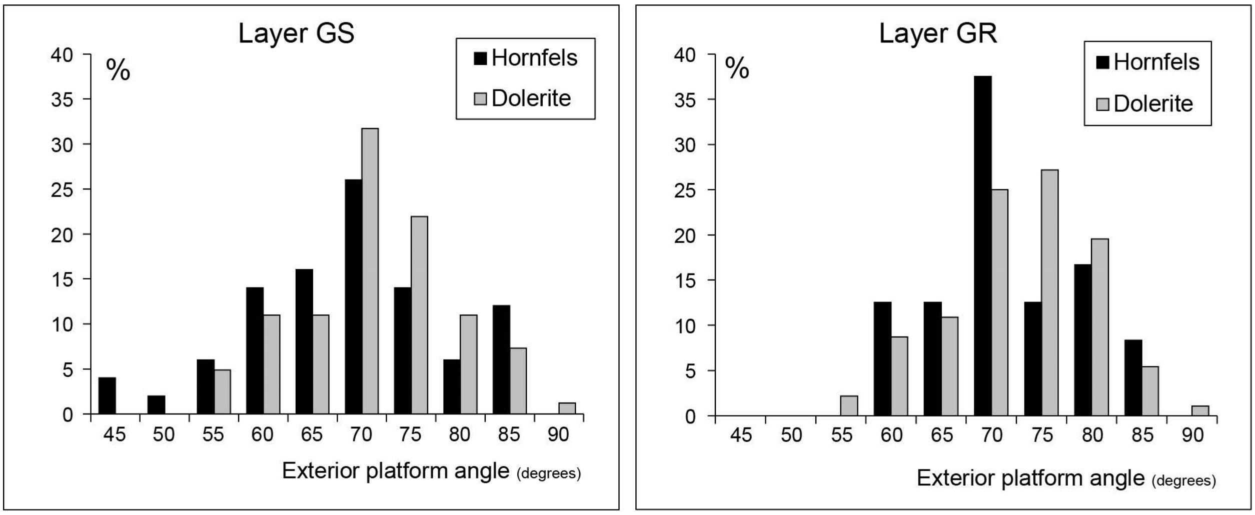

Figure A. Sibudu. Frequency distribution of the exterior platform angle of blades in layers GS and GR.

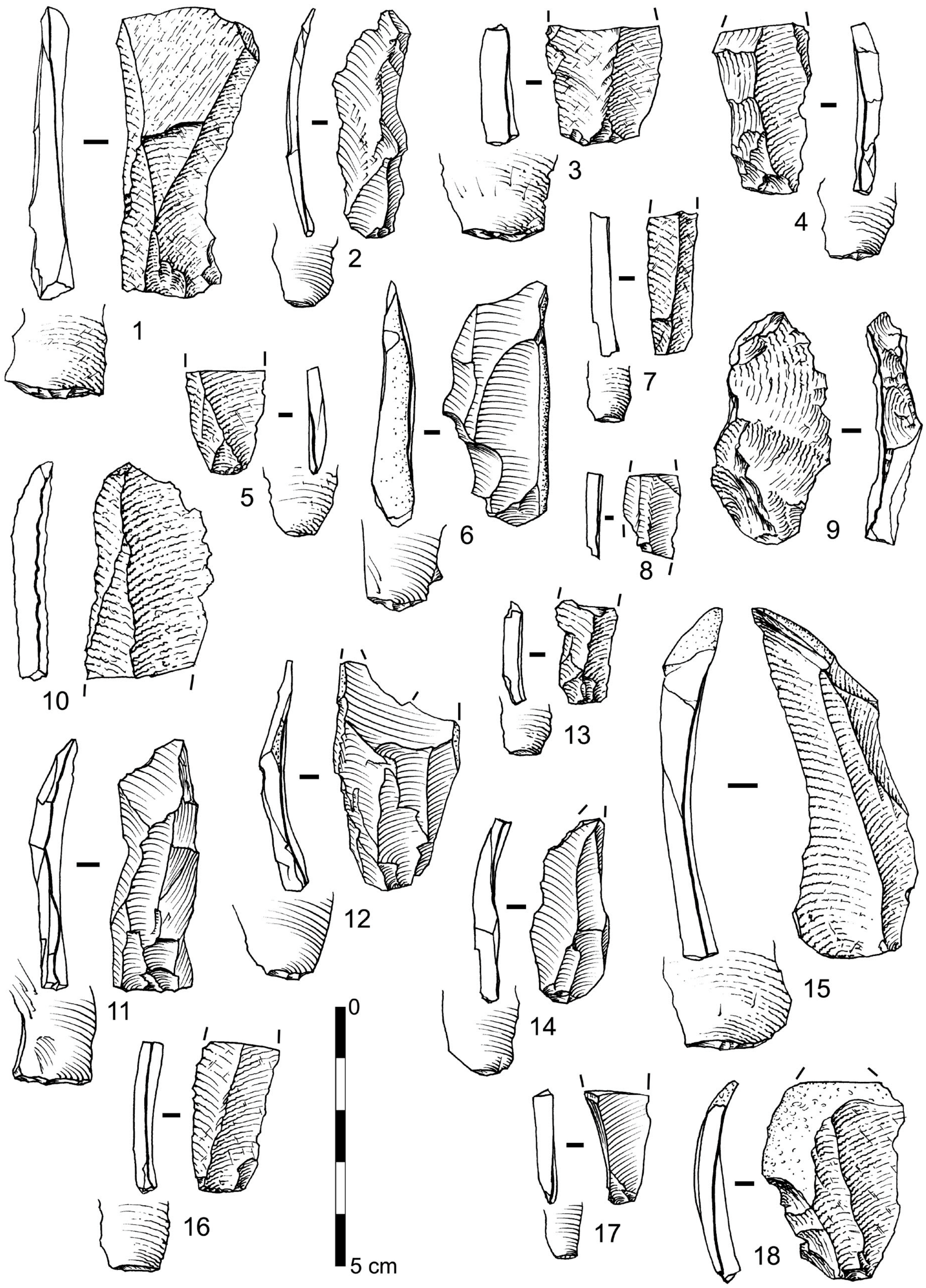

Figure B. Sibudu, Howiesons Poort. Blades and blade fragments from layer GR. 2, 6, 8, 11-12, 14, 17: hornfels; 1, 3-5, 7, 9-10, 13, 15-16, 18: dolerite.

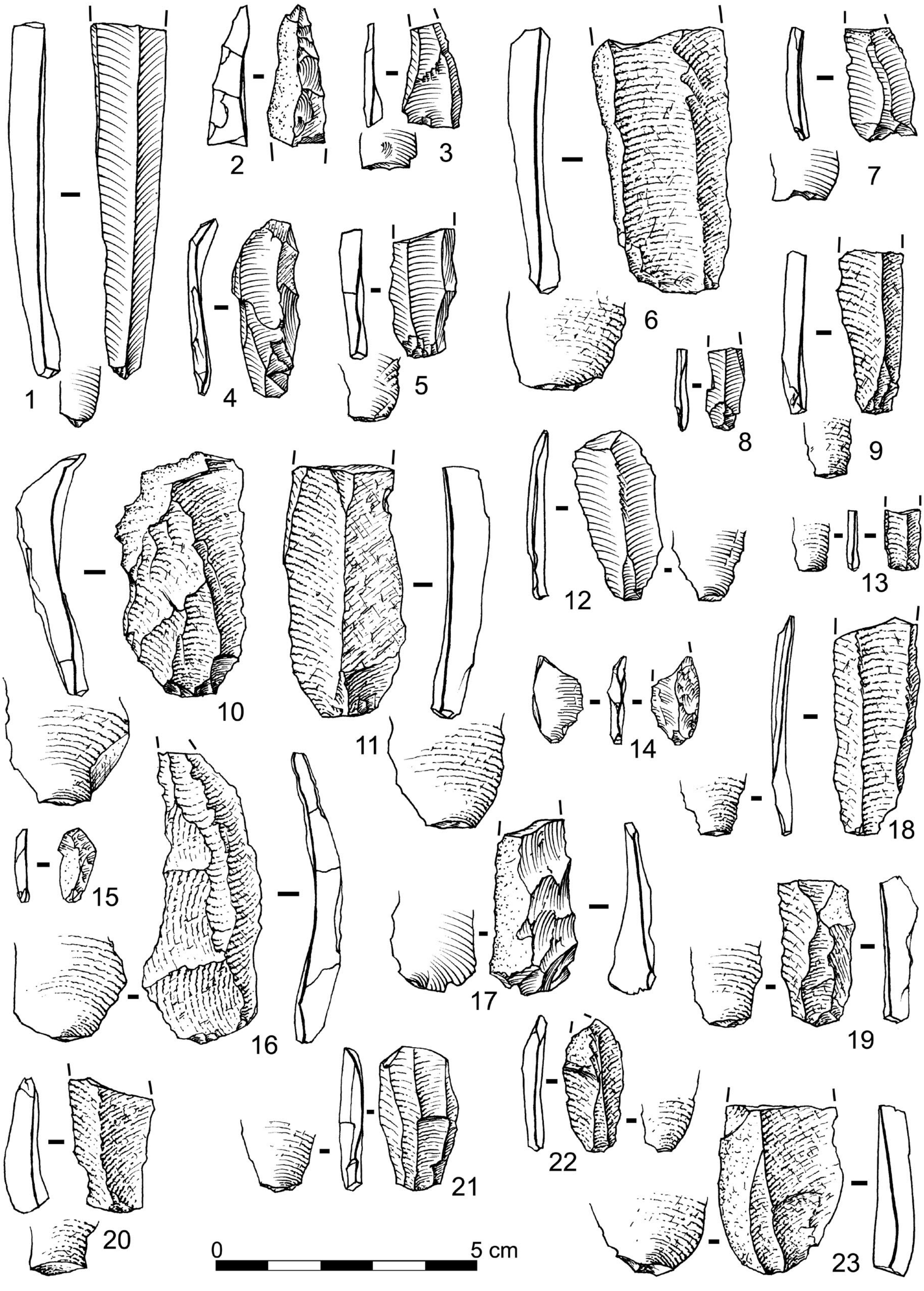

Figure C. Sibudu, Howiesons Poort. Blades and blade fragments from layer GS. 1-5, 7-8, 12, 17: hornfels; 6, 9-11, 13, 16, 18-23: dolerite; 14-15: quartz.

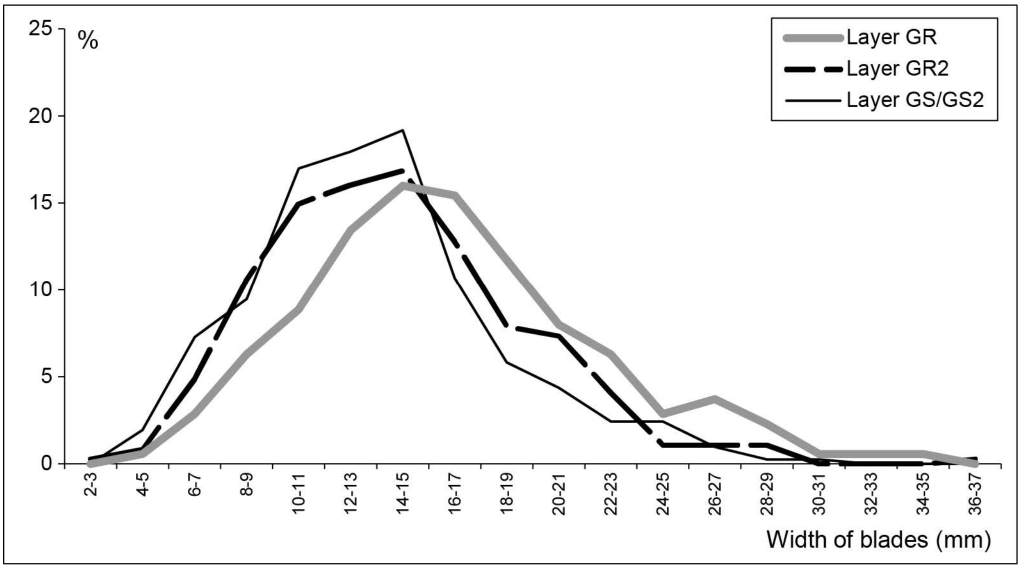

Figure D. Width of blades from Sibudu HP layers GS/GS2 (N=412), GR2 (N=350) and GR (N=368). Two blades from layer GS/GS2 wider than 37 mm are excluded for clarity.

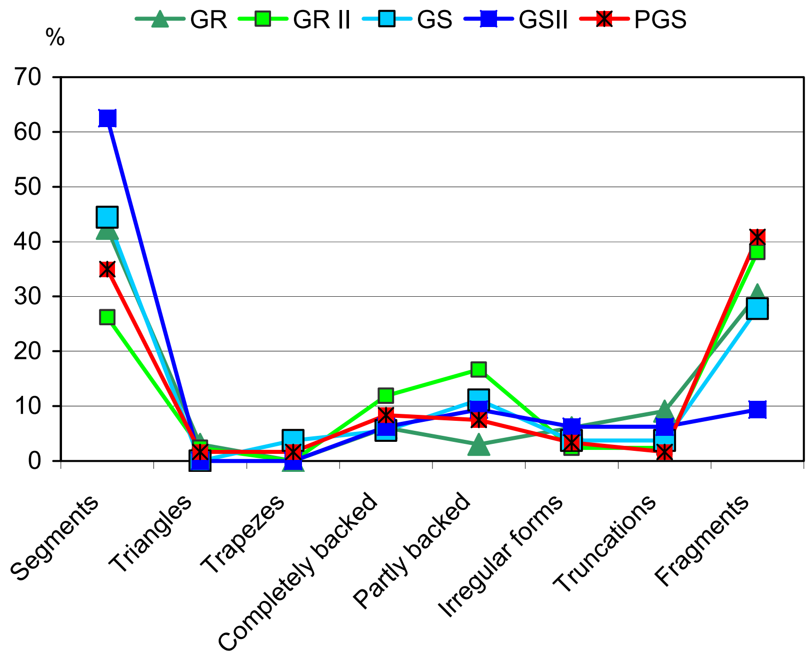

Figure E. Sibudu. Frequencies of classes of backed pieces by levels and sublevels.

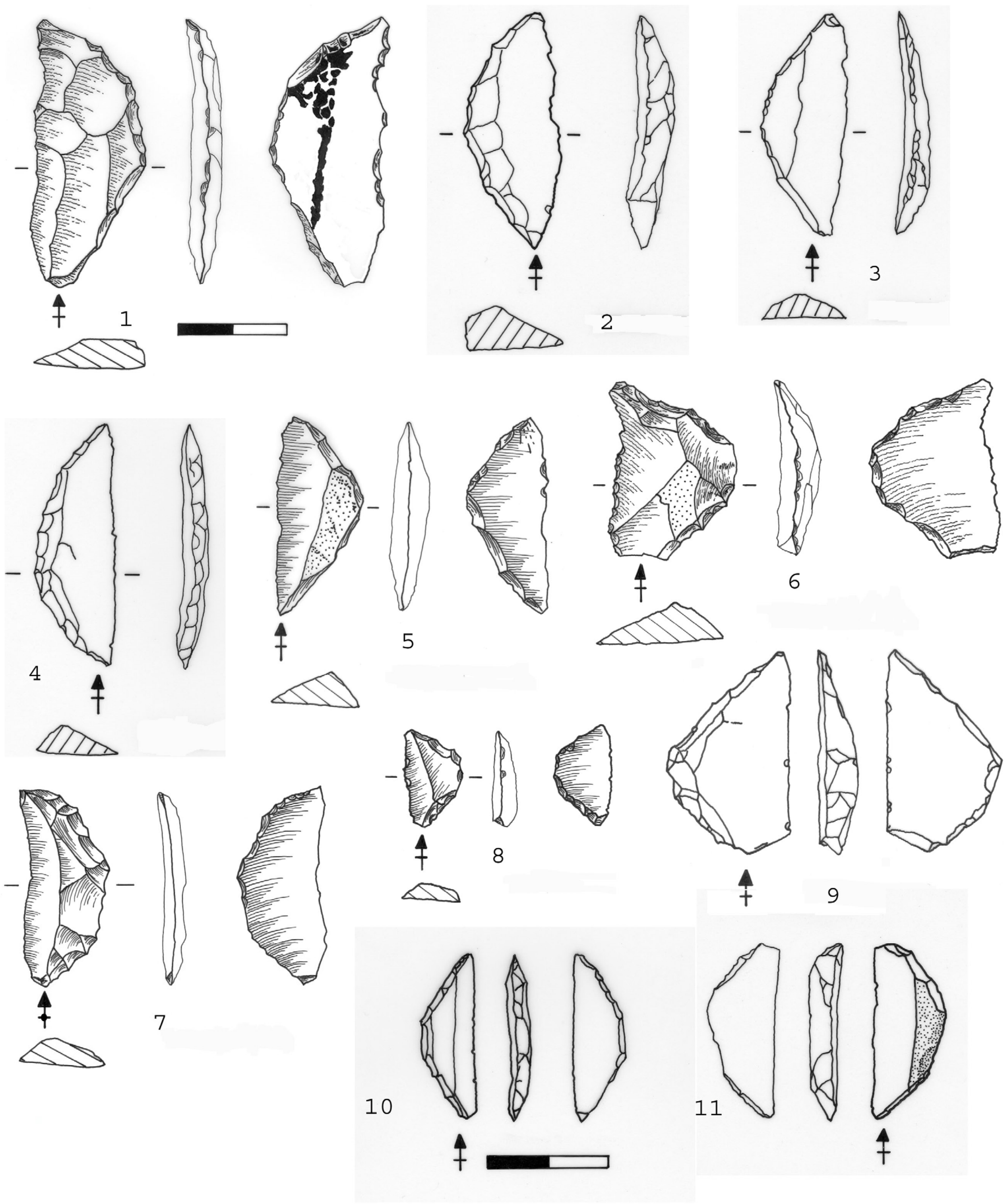

Figure F. Sibudu. Backed pieces from layers GR and GRII, oriented according to the debitage axis, platform missing on all pieces except no.7. In (6) and (9) the platform was removed by a snap fracture. Layer, square, catalogue number, raw material. (1) GR B5a M1 dolerite, segment with an ochre hafting line on the ventral face. This is fig.2 in [1]. (2) GR B6a PM8 dolerite, segment. (3) GR B6b PM10 dolerite, segment. (4) GRII B6a PM7 dolerite, segment. (5) GR B5b M5 hornfels, passing to triangle. (6) GR B5b M4 dolerite, irregular shape. (7) GR B5c M3 hornfels, partly backed. (8) GRII B5c M7 hornfels, passing to triangle. (9) GRII B5b M9 hornfels, triangle. (10) Hearth b in GRII B5a M8 hornfels, segment. (11) Hearth c in GRII B5d M10, hornfels, partly backed. Scale bars = 2 cm.

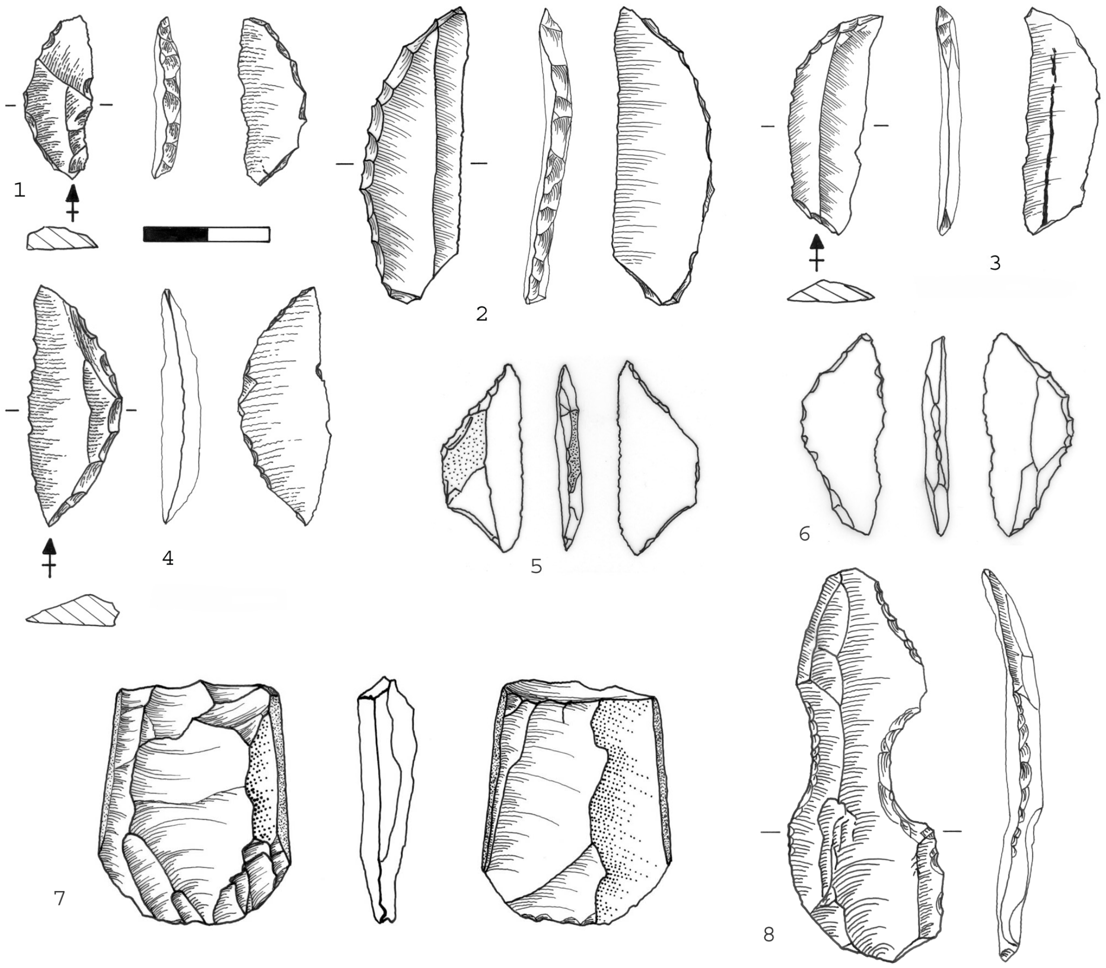

Figure G. Sibudu. (1-6) Backed pieces (1-6) and other tools (7-8) from layers GS and GS II, oriented according to the debitage axis, platform missing on all backed pieces. (1) GS B6a PM13, dolerite segment. (2) GS B6a PM14, hornfels segment. (3) GS B6a 17, hornfels partly backed piece, ochre line on ventral face. (4) GS B6a PM12, dolerite segment. (5) GS B5b M12, hornfels trapeze. (6) GS II B5b M11, dolerite segment. (7) White Ash under GR II (=GS) B6a 10, scaled piece on a hornfel slab. (8) GS B6a 12, hornfels, double notched (strangulated) blade, dihedral platform. Scale bar = 2 cm.

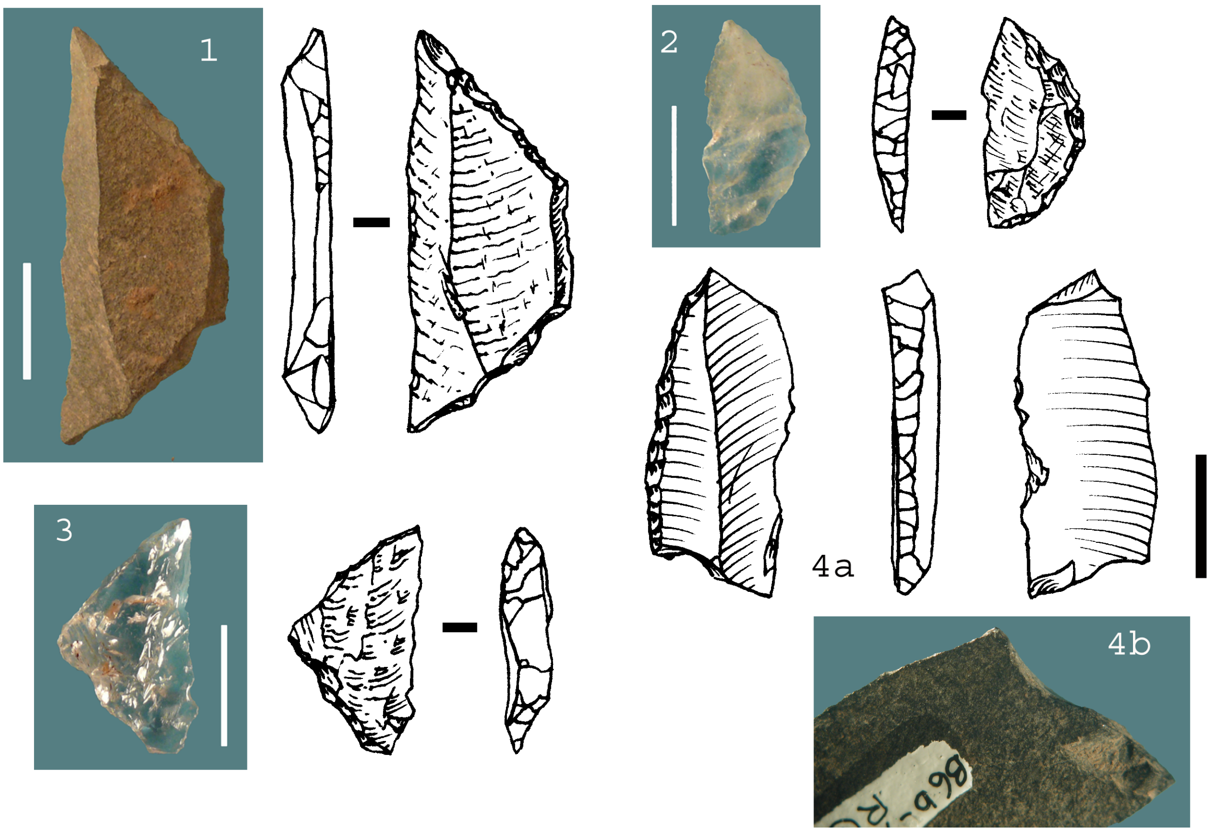

Figure H. Sibudu backed pieces. (1) PGS C6a 47, fine dolerite, trapeze; (2) Hearth in PGS B5a 4, crystal quartz, segment; (3) Hearth in PGS B5d 5, crystal quartz, triangle; (4a, b) Ash in DRGII 6, hornfels, broken piece with a transverse fracture and spin-off of 2.6 mm. Scale bars = 1 cm.

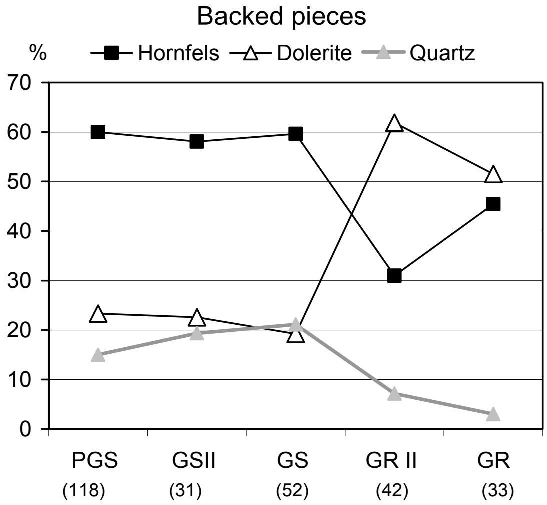

Figure I. Sibudu. Temporal changes in raw material frequencies of backed pieces.

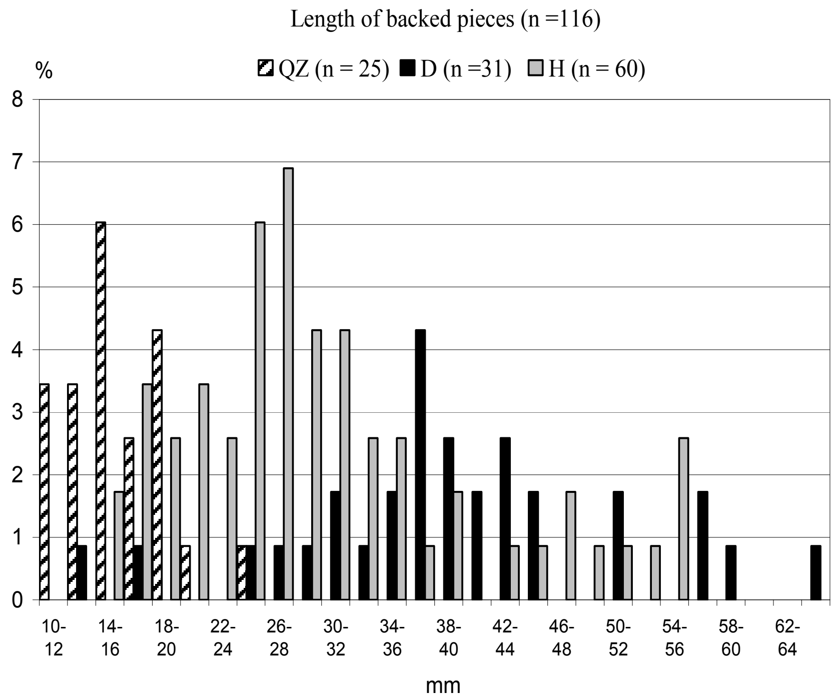

Figure J. Sibudu. Length distribution of backed pieces by raw materials. The quartz pieces are well to the left of the distribution, sharing that position with some dolerite and hornfels pieces. The distribution does not show clear modalities. Truncations and irregular forms are excluded.

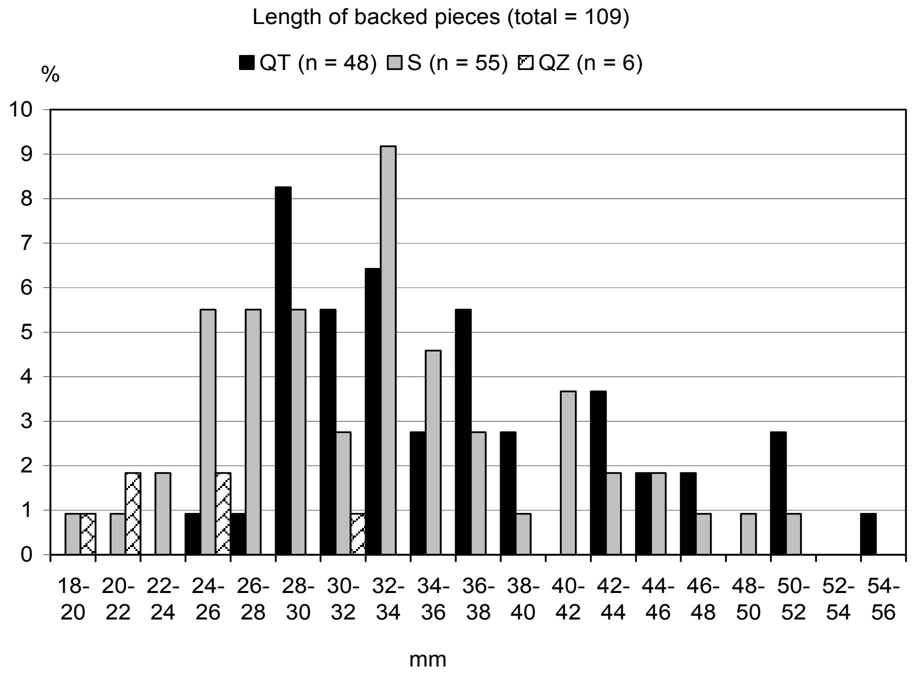

Figure K. Klasies River Cave 1A, layer 20. Length distribution of backed pieces by raw material. As at Sibudu the quartz backed tools are at the left of the distribution but share their position with some silcrete pieces.

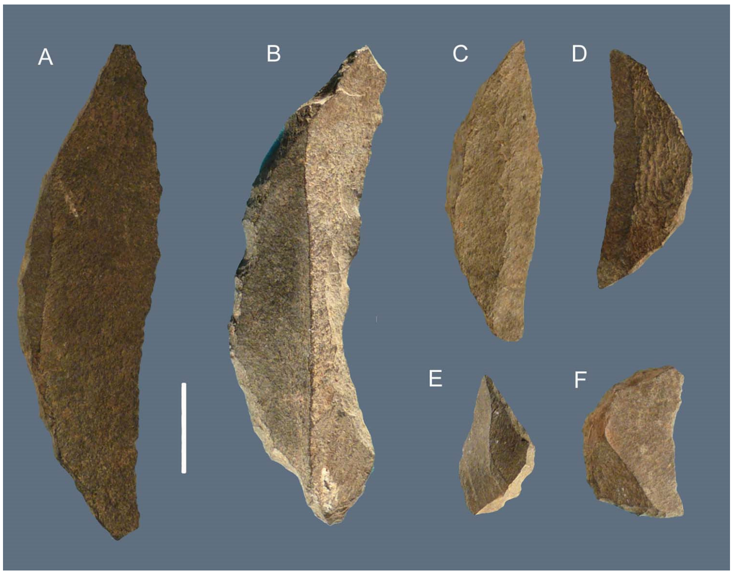

Figure L. Backed pieces of the same raw material (hornfels) but very different sizes. All from layer PGS: nos. 48, 59, 46, 51, 78, 65. Scale bar = 1 cm.

GC/MS analysis of samples of Howiesons Poort segments

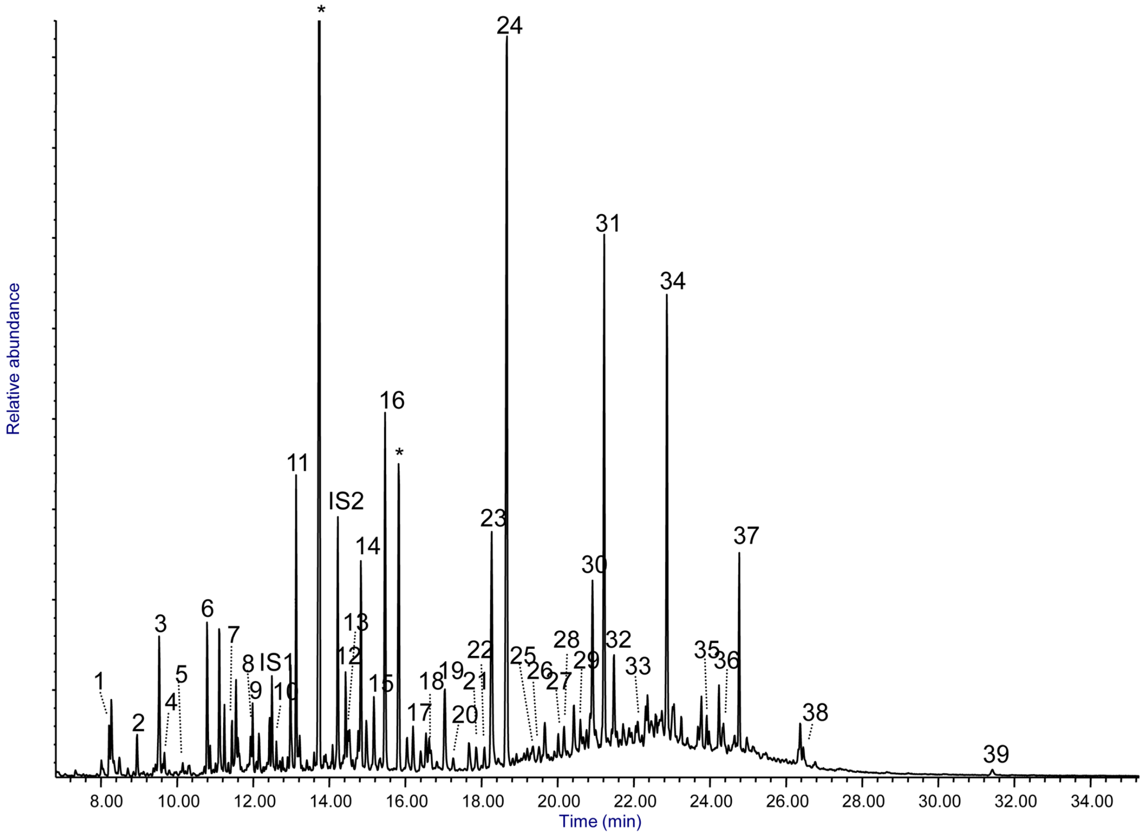

Figure M. Gas chromatogram of the total lipid-resinous fraction of sample B5a PGS (IS1 = hexadecane, IS2 = tridecanoic acid). Peak assignment is reported below.

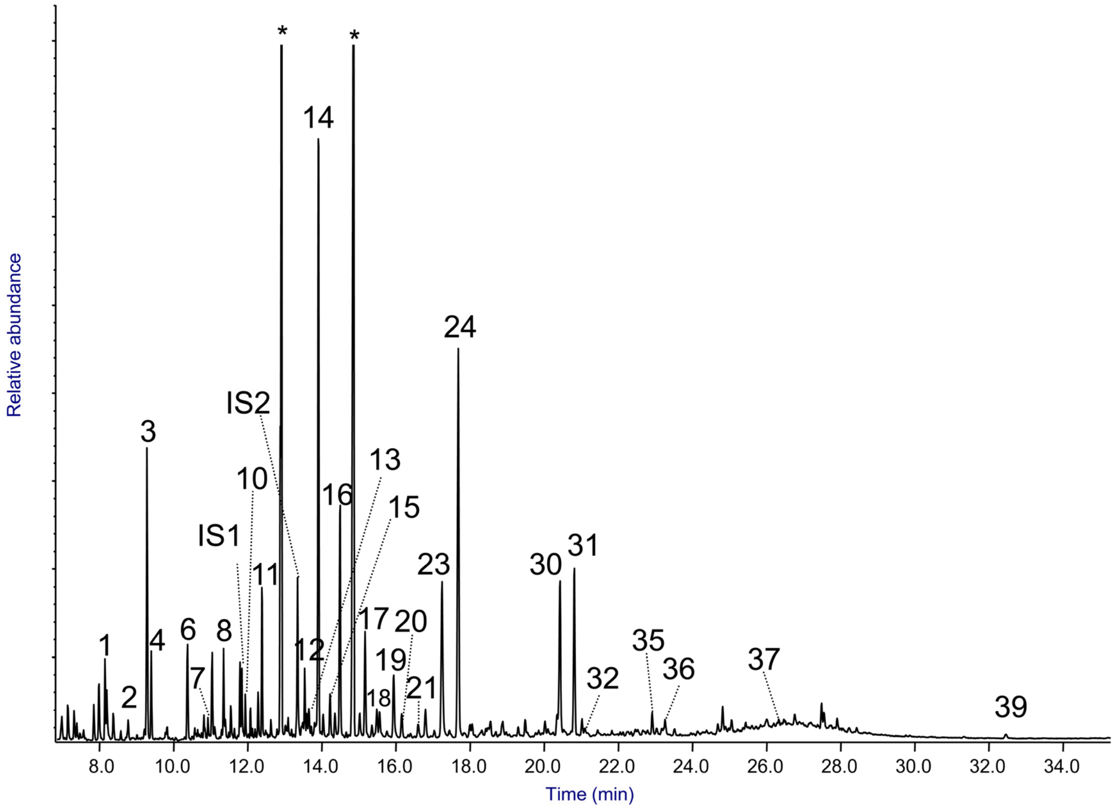

Figure N. Gas chromatogram of the total lipid-resinous fraction of sample C5c GR (IS1 = hexadecane, IS2 = tridecanoic acid). Peak assignment is reported below.

Peak assignment for Figures M and N in S4 File.

Peak #	Tr (min)	Compound	Peak #	Tr (min)	Compound
1	8.29	octanoic acid	20	17.26	hexadecanol
2	8.95	succinic acid	21	17.85	undecandioic acid
3	9.53	nonanoic acid	22	18.08	12-hydroxydodecanoic acid
4	9.68	2-hydroxy heptanoic acid	23	18.27	cis-9-hexadecenoic acid (palmitoleic)
5	10.15	pentanedioic acid (glutaric acid)	24	18.66	hexadecanoic acid (palmitic)
6	10.80	decanoic acid	25	19.35	dodecandioic acid
7	11.44	hexanedioic acid (adipic acid)	26	19.51	13-hydroxytridecanoic acid
8	11.93	2-hydroxy nonanoic acid	27	20.00	heptadecanoic acid (also branched)
9	11.98	undecanoic acid	28	20.17	octadecanol (stearyl alcohol)
IS1	12.49	hexadecane	29	20.61	tridecanedioic acid
10	12.62	heptanedioic acid	30	20.91	cis-9-octadecenoic acid (oleic)
11	13.13	dodecanoic acid (lauric)	31	21.20	octadecanoic acid (stearic)
IS2	14.22	tridecanoic acid	32	21.48	isopimaric acid
12	14.43	p-hydroxy hydrocinnamic acid	33	22.10	16-hydroxyhexadecanoic acid
13	14.48	vanillic acid	34	22.86	dehydroabietic acid
14	14.83	nonandienoic acid (azelaic acid)	35	23.92	didehydroabietic acid
15	15.17	myristoleic acid (9-tetradecenoic acid)	36	24.34	9,10-dihydroxyoctadecanoic acid
16	15.46	tetradecanoic acid (myristic)	37	24.76	7-oxo-dehydroabietic acid
17	16.20	decanedioic acid (sebacic acid)	38	26.45	15-hydroxy-7-oxodehydroabietic acid
18	16.62	pentadecenoic acid	39	31.43	Cholesterol
19	16.51	pentadecanoic acid (also branched)			

The most abundant compounds contained in samples B5a PGS and C6c GR are linear monocarboxylic fatty acids (from C8 to C18), the most abundant being palmitic (hexadecanoic acid, C16:0) and stearic acids (octadecanoic, C18:0). The samples also contain dicarboxylic acids (in particular azelaic acid – nonandioic acid). These oxidation products of unsaturated fatty acids indicate that the lipids originally present in the samples contained a relevant amount of oleic acid (9-octadecanoic), which has undergone a degradative oxidation process, which is also revealed by the presence of 9,10-hydroxy-octadecanoic acids. The presence of odd chain length fatty acids and cholesterol indicates animal origin of fats. Odd-branched (C15 and C17) fatty acids suggest animal fats, or that the lipids have undergone bacterial degradation. On the basis of the observed lipid profile, it is not possible to say whether a vegetal-origin lipid material had been added to the mixture or not. In both samples, a diterpenic fraction was present, whose main components are abietadienic acids: dehydroabietic (#34) , didehydroabietic (#35), 7-oxo-dehydroabietic (#37) and 15-hydroxy-7-oxodehydroabietic (#38). Pimaradienic acids such peak #32 (isopimaric acid) were also detected (pimaric, sandaracopimaric, palustric, levopimaric acids were detected as minor components). The diterpenic profile of the sample suggests that the hafting material contained a resin extracted from a plant belonging to the conifer family. In particular, *Pinaceae*, *Podocarpaceae* or *Araucariaceae* may produce a resin containing abietadienic and pimaradienic components. Amongst our database of South African plants, the wood of *Afrocarpus* (syn.*Podocarpus) falcatus* contains relevant amount of these compounds and is thus a probable candidate as the source of the resin used as hafting material.

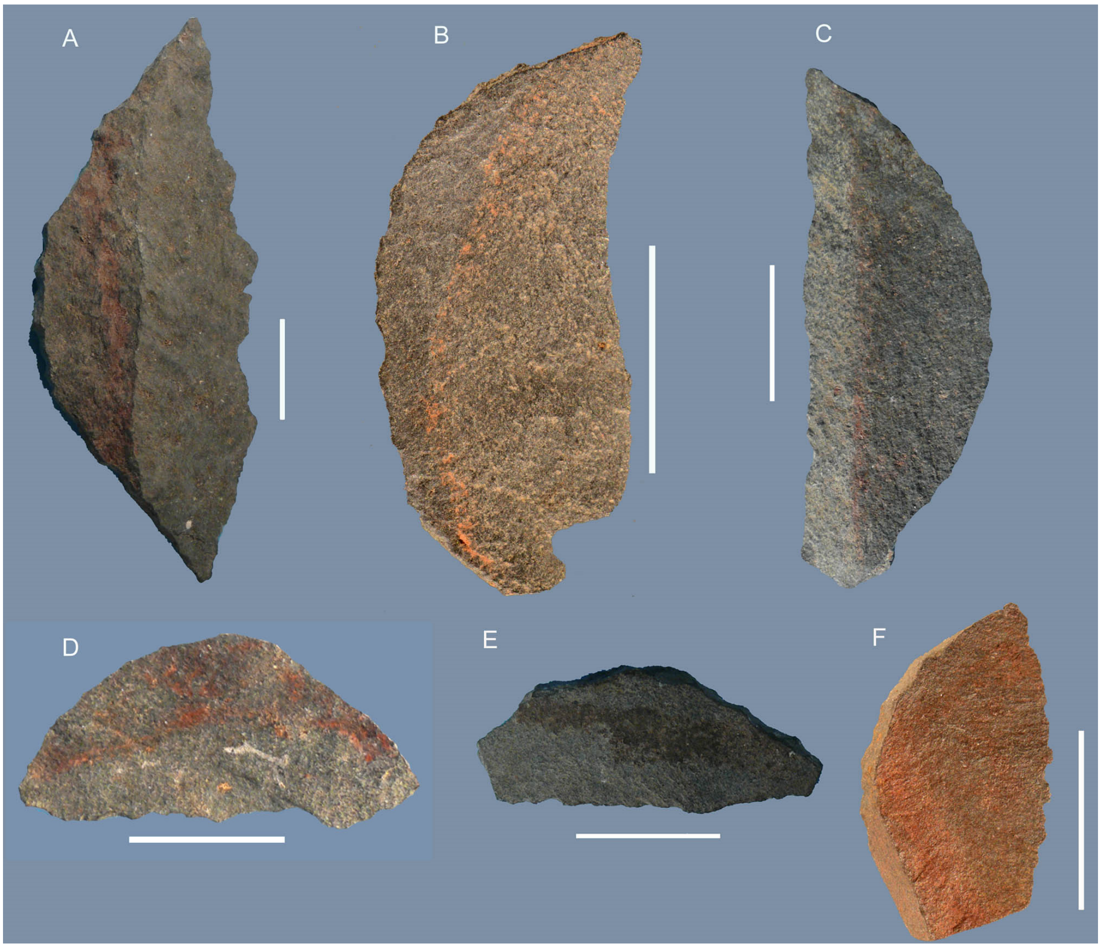

Figure O. Hafting lines on the Sibudu backed pieces can be parallel (A-D) or diagonal (E, F) to the working edge. (A) GRII C5c 97 dolerite; (B) GSII B6a no number; hornfels; (C) PGS C5c 167, hornfels; (D) PGS B5a 13, hornfels; (E) GSII C6c 63, hornfels; (F) GR C6c no number, hornfels. Scale bars = 1 cm.
