# Supplementary material for: The Still Bay and Howiesons Poort at Sibudu and Blombos: Understanding Middle Stone Age Technologies
Source: PLoS One. 2015 Jul 10;10(7):e0131127. doi: 10.1371/journal.pone.0131127 (PMC4498762; doi:10.1371/journal.pone.0131127)
Supplement: S5 File — (PDF) [file pone.0131127.s005.pdf]

The Still Bay and Howiesons Poort at Sibudu and Blombos: Understanding Middle Stone Age technologies

Sylvain Soriano, Paola Villa, Anne Delagnes, Ilaria Degano, Luca Pollarolo, Jeannette J. Lucejko, Christopher Henshilwood, Lyn Wadley

Supporting Information

S5 File

(Tables A-J)

Table A. Frequencies of some attributes observed on the blade platforms in layers GS and GR. Attributes as in [1]. Statistically different frequencies (after a test of comparisons) are indicated in bold[1].

Layers	GS %	GR %
Platform morphology		
Oval or narrow triangular	50.6	48.3
Narrow linear	**14.5**	**23.8**
Curved	7.2	3.3
Quadrangular or wide trapezoidal	27.7	23.8
Platform preparation		
Plain	58.2	59.2
Faceted platforms	23.0	21.1
Spur	2.4	0.7
Trimming of the edge on the exterior core surface	**51.9**	**40.7**
Abrasion of the edge of the platform	18.2	16.6
Bulb morphology		
Lipped, without a bulb	32.3	34.0
Prominent bulb with or without lipping	23.8	20.0
Weakly developed bulb with or without lipping	30.5	28.0
Internal platform delineation		
Regular straight or curved	**64.0**	**52.6**
Overhanging curved platform	12.8	16.4
Overhanging with bulb in clear relief	7.9	11.2
Double curve with two impact points	0.6	1.3
Irregular	14.0	18.4

[1] Preparation of platforms show minimal changes between layers GS and GR. There is a reduction of the trimming of the platform edge (GS: 51.9%, GR: 40.7%; test of equality of two proportions z = 1.98: ∉ $IP_{0.95}$, H_0 rejected, proportions differ) and an increase of narrow linear platforms (GS: 14.5%, GR: 2.8%; test of equality of two proportions, z = 2.11: ∉ $IP_{0.95}$, H_0 rejected, proportions differ). However frequencies for other morphologies are statistically equivalent. Frequency of edge abrasion and of facetted platforms are identical. The morphology of blade platforms is also stable. Changes in the internal platform delination, considered as a proxy of the position of the hammer impact (marginal i.e. on margin of the striking platform or internal i.e. away from the margin), reflect minor modifications of knapping techniques. Frequency of straight or curved delineation of the internal edge is decreasing a little from GS to GR suggesting that bending initiation of the fracture [2] decrease as the percussion became less marginal. Nevertheless, the frequency of lipped platform without a bulb of percussion does not decrease as expected. The

exterior platform angle (Figure A in S4 File) records this minor change in the knapping gesture with differences between GS and GR. Higher angle values are better represented in the GR blades in agreement with a percussion set back from the edge of the striking platform, resulting in more open exterior platform angles. These changes are similar but minor compared to those observed in the late HP of Rose Cottage and Klasies.

Table B. Blanks used for backed pieces.

HP backed pieces	Blades %	Flakes %
Sibudu (n = 199)	97.5	2.5
Rose Cottage (n = 53)	96.2	3.8
Klasies Cave 1A, Singer and Wymer sample (n = 342)	98.8	1.2
Klasies Cave 1A, Deacon's sample (n = 61)	95.1	4.9

Table C. Frequencies of blades from the optimal phase of debitage (i.e. without cortex and from the central part of the debitage surface) used for backed pieces.

Blades from the optimal phase of debitage	N	%
Sibudu (n = 148)	136	91.9
Rose Cottage (n = 42)	24	57.1
Klasies Cave 1A, Singer and Wymer sample (n = 309)	279	90.3
Klasies Cave 1A, Deacon's sample (n = 52)	36	69.2

Table D. Blanks used for all retouched pieces[1]

HP assemblages	Blades %	Flakes %
Sibudu (n = 286)	87.8	12.2
Rose Cottage (n = 94)	90.4	9.6
Klasies Cave 1A, Singer and Wymer sample (n = 436)	88.5	11.5
Klasies Cave 1A, Deacon's sample (n = 155)	81.3	8.7

[1]Indeterminate cases and very rare blanks such as chunks or slabs are excluded. Rose Cottage data is from layers EMD, MAS, ETH and SUZ only. Data for Klasies after [3]. Data for Sibudu include DRG.

Table E. Sibudu. Total of quartz backed pieces, including fragments, in the HP layers.

**Layers**	**Backed pieces, all raw materials**	**Quartz backed pieces**	
	N	N	%
GR	75	4	5.3
GS	86	17	19.8
PGS	120	18	15.0

Table F. Proportions of "hunting" tools and other tools in the HP and post-HP of Sibudu and Rose Cottage.

	**Backed pieces**		**Other tools**	
**Howiesons Poort**	N	%	N	%
Sibudu GS+GR (n = 233)	161	69.1	72	30.9
Rose Cottage EMD+MAS (n = 69)	43	62.3	26	37.7

	**Unifacial points**		**Other tools**	
**Post-HP**	N	%	N	%
Sibudu layer RSP (n = 344)	138	40.1	206	59.9
Rose Cottage layer THO (n = 167)	19	11.4	148	88.6

Table G. Sibudu HP. Mean width of blade, quartz excluded

**Layer**	**N**	**Mean width (mm)** [1]	**SD**
GR	350	16.33	5.51
GR2	368	14.22	4.90
GS/GS2	414	13.57	5.06

[1]Blades are of similar width in layers GS/GS2 (mean=13.57mm) and GR2 (mean=14.22 mm; Z-test: $Z_0 = 1.83 < Z_\alpha$, 5%, means are statistically similar) but comparison of mean width between layer GR2 and GR (mean=16.33 mm; Z-test: $Z_0 = 5.41 > Z_\alpha$, 5%, means are statistically different) argue for an increase of blade width.

Table H. Sibudu. Mean thickness and width of blade platforms for layer GS and GR, quartz excluded[1].

Blade platform		Layer GS			Layer GR		
		Dolerite	Hornfels	All raw mat.	Dolerite	Hornfels	All raw mat.
Thickness	Mean (mm)	3.71	3.04	3.45	4.15	3.02	3.9
	SD	1.48	1.53	1.52	2.03	1.99	2.05
Width	Mean (mm)	9.85	8.25	9.28	11.06	9.02	10.65
	SD	3.63	3.6	3.76	4.34	4	4.26

[1]Platforms of blades from GS and GR are similar in thickness (Z-test: $Z_0 = 1.34 < Z_\alpha$, 5%, H_0 validated) but differ slightly in width (GS mean = 9.28, GR mean = 10.65; Z-test: $Z_0 = 3.70 > Z_\alpha$ 5%, means are statistically different).

Table I. Frequency of impact scars of points of known function in Europe (arrowheads) and North America Paleoindian spear tips from Casper, Wyoming)[1].

Site, age and kinds of points	Type of site	No. of points suitable for analysis	No. of impact scars	% of impact scars
Ommelshoved, about 13,000 BP, tanged points	Residential	88	11	12.5
Bromme, about 13,000 BP, tanged points	Residential	47	3	6.4
Muldbjerg, about 2800 bc, transverse arrowheads	Residential	30	9	30.0
Præstelyng, about 3200 bc, transverse arrowheads	Residential	56	8	14.3
Vejlebro, level 8, about 3500 bc, transverse arrowheads	Residential	24	5	20.8
Vejlebro, level 9, about 3500 bc, transverse arrowheads	Residential	42	2	4.8
Stellmoor, upper level, about 12,000 BP, tanged points	Reindeer kill site MNI = 302	45	19	42.2
Casper, 10 060 ±170 BP with Hell Gap bifacial points	Bison kill site MNI = 74	60	26	43

[1] Modified after [4], references therein.

Table J. Frequencies of backed pieces in post-HP, late and final MSA assemblages.

Site	Age[1] OSL dates ka (unless specified)	Retouched pieces (except backed) N	Backed pieces N	Backed pieces %	Main tool forms
Sibudu, BM-BSP[2]	58.5 ±1.4 weighted mean age	551 (excluding hammerstones)	4	1.0	Unifacial points, scrapers, retouched and utilized blades
Sibudu, late MSA, layer RSp[3]	46.0 ± 1.9	343	1	0.3	Unifacial points, scrapers, pièces esquillées
Sibudu, late and final MSA, MOD-CO	49.4 ± 2.1 to 38.0 ± 2.6	377	15	4.0	Unifacial and bifacial points, scrapers, retouched flakes
Klasies River Cave 1A, MSA III Deacon's sample	57.9 ± 2.3	103	0	0	Scrapers, burins, denticulates, notches, retouched and utilized blades and flakes
Rose Cottage, post-HP layers, Byr, Tho Ela, Lyn, Kar	56.0 ±2.3 (sample from layer Lin)	230	13	5.3	Scrapers, unifacial points, retouched blades and flakes
Border Cave, layers 2WA, 2BS LowerA+B and 2BS UP (2BS assemblages are transitional to ELSA)[5]	60 ± 3 (ESR) to 49-45 (^{14}C cal BP)	67	3	4.3	Unifacial points, scrapers

[1][5]. [2][6]. [3][7]. [4]The backed pieces of layers MOD to CO at Sibudu (include all final MSA layers LBMOD, Beach, Bu, Mou, Es, MC, Co) are all on flakes and have a very different morphology from the HP backed pieces. [5][8].

References

1. Soriano S, Villa P, Wadley L. Blade technology and tool forms in the Middle Stone Age of South Africa: the Howiesons Poort and post-Howiesons Poort at Rose Cottage Cave. J Archaeol Sci. 2007;34: 681–703.
2. Cotterell B, Kamminga J. The Formation of Flakes. Am Antiq. 1987;52: 675–708. doi:10.2307/281378
3. Villa P, Soriano S, Teyssandier N, Wurz S. The Howiesons Poort and MSA III at Klasies River main site, cave 1A. J Archaeol Sci. 2010;37: 630–655.
4. Villa P, Soressi M, Henshilwood CS, Mourre V. The Still Bay points of Blombos Cave (South Africa). J Archaeol Sci. 2009;36: 441–460.

5. Jacobs Z, Roberts RG, Galbraith RF, Deacon HJ, Grün R, Mackay A, et al. Ages for the Middle Stone Age of Southern Africa: Implications for Human Behavior and Dispersal. Science. 2008;322: 733–735.
6. Will M, Bader GD, Conard NJ. Characterizing the Late Pleistocene MSA Lithic Technology of Sibudu, KwaZulu-Natal, South Africa. PLoS ONE. 2014;9: e98359. doi:10.1371/journal.pone.0098359
7. Villa P, Delagnes A, Wadley L. A late Middle Stone Age artifact assemblage from Sibudu (KwaZulu-Natal) : comparisons with the European Middle Paleolithic. J Archaeol Sci. 2005;32: 399–422.
8. Villa P, Soriano S, Tsanova T, Degano I, Higham TF, d' Errico F, et al. Border Cave and the beginning of the Later Stone Age in South Africa. Proc Natl Acad Sci U S A. 2012;109: 13208–13213. doi:10.1073/pnas.1204213109
