# Supplementary material for: The Still Bay and Howiesons Poort at Sibudu and Blombos: Understanding Middle Stone Age Technologies
Source: PLoS One. 2015 Jul 10;10(7):e0131127. doi: 10.1371/journal.pone.0131127 (PMC4498762; doi:10.1371/journal.pone.0131127)
Supplement: S6 File — (PDF) [file pone.0131127.s006.pdf]

The Still Bay and Howiesons Poort at Sibudu and Blombos: Understanding Middle Stone Age technologies

Sylvain Soriano, Paola Villa, Anne Delagnes, Ilaria Degano, Luca Pollarolo, Jeannette J. Lucejko, Christopher Henshilwood, Lyn Wadley

Supporting Information

S6 File

Pressure flaking in the Howiesons Poort

Pressure flaking has been documented in the final retouch phases of the Still Bay bifacial points of Blombos to produce thin, V-shaped tips with straight edges using heat-treated silcrete [1]. However there is no evidence of pressure retouch on any of the Still Bay bifacial points from Sibudu. In a recent paper [2], three bone tools from Sibudu have been described as pressure flakers. Two of these tools come from layer GR and one from layer Yellow Ash 2 which is above layer GR and contains a post-HP assemblage dated to ca. 60-58 ka by OSL. Thus the use of pressure flaking for final shaping of Sibudu HP bifacial pieces of quartz was considered possible [3] and it was suggested that the pressure flakers could also have been used for the backing of segments and for retouch of other formal tools in the Howiesons Poort.

The blades and bladelets of the HP were made by direct marginal percussion, using a soft stone hammer at Rose Cottage, Klasies [4] and Sibudu. Could pressure have been used for making a back on blades as documented in the Upper Paleolithic?

The criteria for identification of pressure on backed blades have been described by Pelegrin [5] based on experimental series and observations of Magdalenian backed bladelets and recognized on Gravettian backed points by Klaric [6]. A specific feature are retouch scars which plunge abruptly on the adjacent face of the blade (Figure A). This feature does not seem to occur on the HP backed pieces from Sibudu. On the contrary, there is good evidence for the use of direct percussion for backing retouch. Retouch scars produced by direct percussion with a stone hammer tend to be deep and not so regularly spaced. More telling are knapping accidents which can occur if the hammer strikes the blade not on the margin but more internally, away from the margin; the resulting fracture produces an overshot flake which removes a portion of the back (Figure B). These knapping accidents (called Krukowski microburins, [7]) have been experimentally replicated by De Wilde and De Bie [8]. For an archaeological example refitted to a Châtelperronian point see Aubry et al. ([9] : fig. 7A). Overshot flakes of this kind clearly showing the use of a stone hammer for backing occur in all HP layers (6 in PGS, 1 in GS, 2 in GR, 1 in DRG).

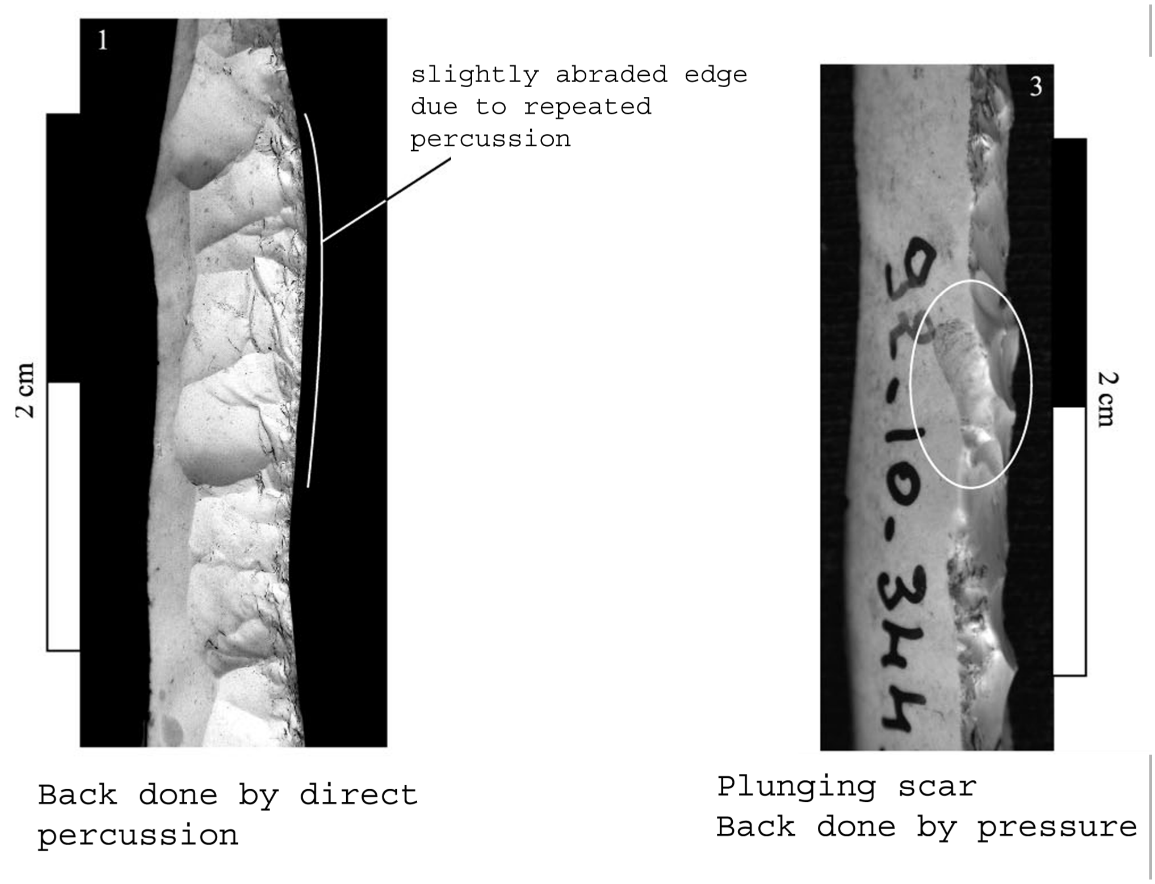

Figure A. Backing by direct percussion and by pressure on Gravettian pieces from the site of Cirque de la Patrie (Seine-et-Marne, France; [6]). Courtesy of Laurent Klaric.

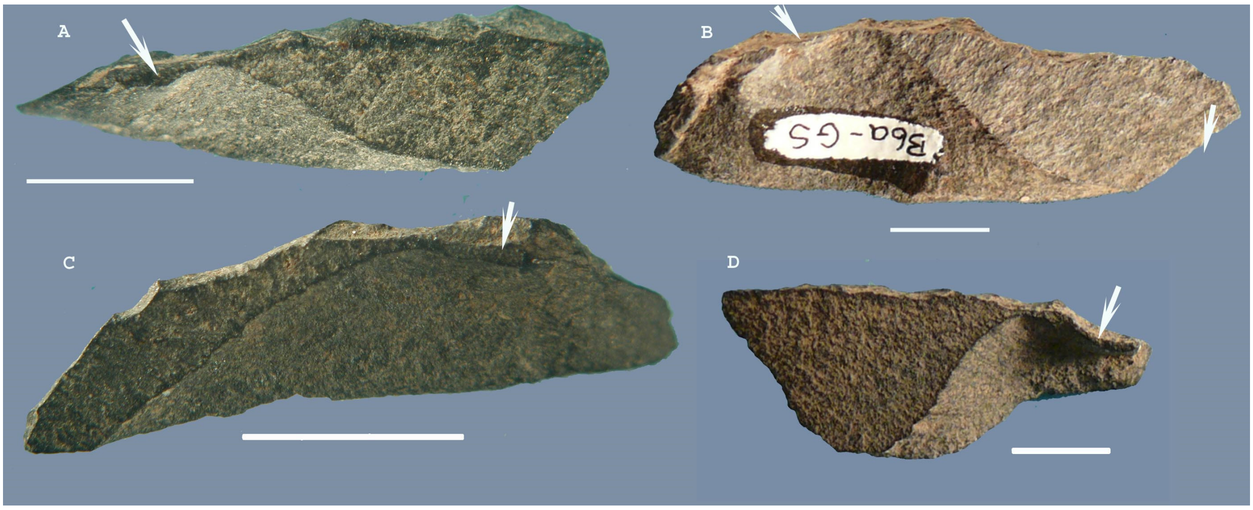

Figure B. Overshot flakes that have removed a portion of the back of a backed piece. These are knapping accidents proving the use of direct percussion for backing retouch; all hornfels. (A) PGS B5 31. (B) GS B6a 16. (C) GRIIB5b 41. (D) DRGII B6a BP1. The white arrow indicates the bulb of percussion. Scale bars = 5 mm.

References

1. Mourre V, Villa P, Henshilwood CS. Early Use of Pressure Flaking on Lithic Artifacts at Blombos Cave, South Africa. Science. 2010;330: 659–662.

2. D' Errico F, Backwell LR, Wadley L. Identifying regional variability in Middle Stone Age bone technology: The case of Sibudu Cave. J Archaeol Sci. 2012;39: 2479–2495.

3. De la Peña P, Wadley L, Lombard M. Quartz Bifacial Points in the Howiesons Poort of Sibudu. South Afr Archaeol Bull. 2013;68: 119–136.

4. Villa P, Soriano S, Teyssandier N, Wurz S. The Howiesons Poort and MSA III at Klasies River main site, cave 1A. J Archaeol Sci. 2010;37: 630–655.

5. Pelegrin J. Sur les techniques de retouche des armatures de projectile. In: Pigeot N, editor. Les derniers magdaléniens d'Étiolles Perspectives culturelles et paléohistoriques (l'unité d'habitation Q31). Paris: CNRS éditions; 2004. pp. 161–166.

6. Klaric L. L'unité technique des industries à burins du Raysse dans leur contexte diachronique: réflexions sur la diversité culturelle au Gravettien à partir des données de la Picardie, d'Arcy-sur-Cure, de Brassempouy et du Cirque de la Patrie. Thèse de Doctorat, Université Panthéon-Sorbonne. 2003. Available: http://www.theses.fr/2003PA010650

7. Brézillon M. La dénomination des objets de pierre taillée. Matériaux pour un vocabulaire des préhistoriens de langue française. Paris: Ed. CNRS; 1968.

8. De Wilde D, De Bie M. On the origin and significance of microburins: an experimental approach. Antiquity. 2011;85: 729–741.

9. Aubry T, Dimuccio LA, Almeida M, Buylaert J-P, Fontana L, Higham T, et al. Stratigraphic and technological evidence from the middle palaeolithic-Châtelperronian-Aurignacian record at the Bordes-Fitte rockshelter (Roches d'Abilly site, Central France). J Hum Evol. 2012;62: 116–137.
